# Characterization of structural variation in Tibetans reveals new evidence of high-altitude adaptation and introgression

**DOI:** 10.1186/s13059-021-02382-3

**Published:** 2021-05-25

**Authors:** Cheng Quan, Yuanfeng Li, Xinyi Liu, Yahui Wang, Jie Ping, Yiming Lu, Gangqiao Zhou

**Affiliations:** 1 Department of Genetics & Integrative Omics, State Key Laboratory of Proteomics, National Center for Protein Sciences, Beijing Institute of Radiation Medicine, 27 Taiping Road, Beijing, 100850 People’s Republic of China; 2 Hebei University, Baoding, Hebei Province 071002 People’s Republic of China; 3 Collaborative Innovation Center for Personalized Cancer Medicine, Center for Global Health, School of Public Health, Nanjing Medical University, Nanjing, Jiangsu Province 211166 People’s Republic of China; 4 Medical College of Guizhou University, Guiyang, Guizhou Province 550025 People’s Republic of China

**Keywords:** Structural variation, Long-read sequencing, Tibetan, High-altitude adaptation

## Abstract

**Background:**

Structural variation (SV) acts as an essential mutational force shaping the evolution and function of the human genome. However, few studies have examined the role of SVs in high-altitude adaptation and little is known of adaptive introgressed SVs in Tibetans so far.

**Results:**

Here, we generate a comprehensive catalog of SVs in a Chinese Tibetan (n = 15) and Han (n = 10) population using nanopore sequencing technology. Among a total of 38,216 unique SVs in the catalog, 27% are sequence-resolved for the first time. We systematically assess the distribution of these SVs across repeat sequences and functional genomic regions. Through genotyping in additional 276 genomes, we identify 69 Tibetan-Han stratified SVs and 80 candidate adaptive genes. We also discover a few adaptive introgressed SV candidates and provide evidence for a deletion of 335 base pairs at 1p36.32.

**Conclusions:**

Overall, our results highlight the important role of SVs in the evolutionary processes of Tibetans’ adaptation to the Qinghai-Tibet Plateau and provide a valuable resource for future high-altitude adaptation studies.

**Supplementary Information:**

The online version contains supplementary material available at 10.1186/s13059-021-02382-3.

## Background

Structural variation (SV) is usually defined as the intra- or inter-chromosomal genomic rearrangement acting as a significant mutational force shaping the evolution and function of the genome [1]. In the face of high gene flow caused by migration load, SVs can generate incompatible alleles allowing the beneficial variant to reduce the risk of recombination with maladapted genomic backgrounds, thereby maintaining allele frequency (AF) clines towards environmental gradients [2, 3].

The adaptation of Tibetan highlanders to the Qinghai-Tibet Plateau with an average elevation of over 4000 m is a representative case of anatomically modern human (AMH) conquering new environmental conditions [4]. Previous studies usually used single nucleotide variants (SNVs) to search for selection evidence in the Tibetan genome and successfully identified two essential genes related to the hypoxia-inducible transcription factor (HIF) pathway, endothelial PAS domain protein 1 (*EPAS1*) and egl-9 family hypoxia-inducible factor 1 (*EGLN1*) [4–6]. The adaptive alleles of these two genes could help maintain the hemoglobin concentration so that red cells would not be overproduced at high altitudes [7, 8]. However, few studies have examined the role of SVs underlying high-altitude adaptation (HAA). In an earlier study [9], a 3.4-kilobase (kb) Tibetan-enriched deletion (TED) located at 80 kb downstream of *EPAS1* had been identified using microarray data, indicating that SVs also play a role in the process of Tibetans’ adaptation to the plateau. Besides, the discovery of a particular Tibetan-Denisovan haplotype motif characterized by derived alleles from five SNVs in the *EPAS1* region indicated the signature of introgression from extinct non-AMH populations to Tibetans [4, 10, 11]. Although several recent studies have found that SVs originated from archaic hominins and introgressed into AMH substantially contribute to local population adaptation [12, 13], little is known of adaptive introgressed SVs in Tibetans so far.

In recent years, long-read sequencing (LRS) technologies, such as PacBio single-molecule real-time (SMRT) sequencing and nanopore sequencing, have been introduced to resolve SVs in the human genome [14, 15]. Long and contiguous reads generated from these emerging technologies can span SVs’ breakpoints, enabling more accurate alignment to repetitive sequences [16]. Thus, LRS makes it possible to detect previously unresolved SVs with high sensitivity [17–19], facilitating a better evaluation of SVs’ impact on HAA. A recent study sequenced one single adult male of native Tibetan ancestry using the PacBio SMRT sequencing platform and identified a deletion of 163 base pairs (bp) located in an intronic region of myocardin-related transcription factor A (*MRTFA*), which is associated with lower systolic pulmonary arterial pressure in Tibetans [20]. However, it is still necessary to use population-based detection methods to explore SVs related to HAA.

In this study, we sought to comprehensively characterize the complete spectrum of SVs in a Chinese Tibetan and Han population using nanopore sequencing technology. We performed a population-based SV detection in order to include as many common variants as possible. Among a total of 38,216 unique SVs in the catalog, we identified 69 Tibetan-Han stratified SVs and 80 candidate adaptive genes, providing valuable resources for future HAA studies. Furthermore, we scanned signatures of natural selection and archaic introgression around the population-stratified SVs to explore how SVs introgressed from hominins could facilitate HAA.

## Results

### Discovery of SVs in a Tibetan and Han population by long-read sequencing

**Fig. 1:**
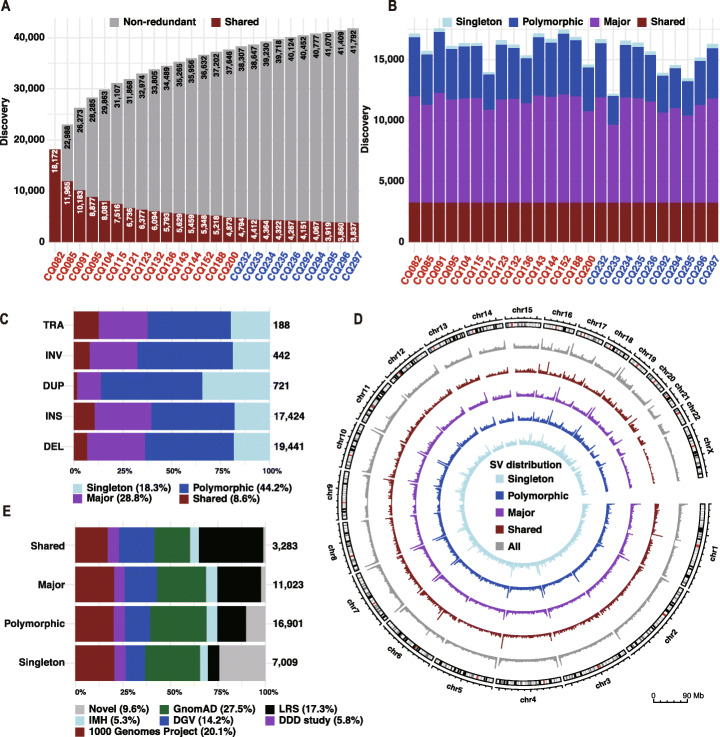
Discovery of structural variations in 15 Tibetan and 10 Han samples. **A** Structural variations (SVs) discovered from each sample were merged into a non-redundant set. Shared SVs are shown as red portions of each bar. Red are Tibetans and blue are Hans. **B** The number of SVs for each discovery category is shown per sample, including shared (identified in all samples), major (identified in ≥ 50% of samples), polymorphic (identified in > 1 sample), and singleton (identified in only one sample) SVs. **C** The frequencies for each SV type: translocation (TRA), inversion (INV), duplication (DUP), insertion (INS), and deletion (DEL). **D** The circular layout of SV distribution in 500-kb windows for each discovery category. **E** Proportions for SVs discovered in previously published SV calls for each discovery category. LRS represents SV calls identified from long-sequence data including HX1, ZF1, and a multi-population study published by Audano et al. IMH represents a common disease trait mapping study published by Ira M. Hall’s lab. DDD study represents the Deciphering Developmental Disorders Study

Long-read sequencing data were generated from 15 Tibetan and 10 Han genomes using nanopore sequencing (Additional file 1: Table S1). For each genome, we detected SVs relative to GRCh37 human reference assembly using a multi-platform pipeline (“Methods” section). On average, 15,813 SVs per sample were identified and were then merged into a non-redundant set of 41,792 SVs (Fig. 1A, B). Consistent with a previous study [14], the size of the non-redundant set grew rapidly at the beginning and gradually slowed down with the increase of samples, suggesting that a considerable proportion of common variations were detected in this Chinese Tibetan and Han population. Then, we filtered out 3576 unreliable genotypes with a hard threshold and obtained a total of 38,216 unique SVs (“Methods” section). Nearly 74% (n = 27,994) of these SVs have breakpoints within an interval of 100 bp across different samples (Additional file 1: Fig. S1A). All subsequent analyses were then performed on this final set of SVs, which comprise 19,441 deletions, 17,424 insertions, 721 duplications, 442 inversions, and 188 translocations (Fig. 1C; Additional file 1: Fig. S1B and S1C; Additional file 2: Table S2). Contrary to the results observed using PacBio SMRT-SV [14], we identified more deletions than insertions using nanopore sequencing. This phenomenon might result from base-calling errors of homopolymer regions, which is still the main drawback of nanopore sequencing [15, 21–23]. Meanwhile, we identified a relatively small number of duplications, mainly because the majority of common duplications should be classified as multi-allelic SVs [24], which is beyond the scope of this study.

As suggested in a previous report [14], we classified these SVs into four categories: shared (identified in all samples), major (identified in ≥ 50% of samples), polymorphic (identified in > 1 sample), and singleton (identified in only one sample) SVs. Consistent with the results observed using PacBio SMRT-SV [14], nearly 40% (n = 14,306) of the SVs were identified as major or shared variants, and the proportion of shared insertions was higher than that of deletions (Fig. 1C; Additional file 1: Fig. S1B and S1C). The frequencies of SVs in all categories decreased markedly with length, and > 88% (n = 33,680) of these SVs have a length ranging from 50 bp to 1 kb (Additional file 1: Fig. S1D and S1E). As reported in previous studies [14, 25], we estimated a fourfold increase in SV density within the last five megabase (Mb) of chromosome arms (p-value < 0.001, permutation test), with the largest increase for major variants (fold change [FC] = 5.48) and the lowest for shared variants (FC = 4.18) (Fig. 1D and Additional file 1: Fig. S2).

We compared our non-redundant set with previously published SV calls identified from thousands of genomes and calculated the maximum variant frequency in these datasets (“Methods” section). We found that 27.0% (n = 10,308) of the SVs discovered in our data are novel ones that have not been sequence-resolved by short-read sequencing (Fig. 1E; Additional file 1: Fig. S1F and S1G). Moreover, there were 842 shared variants and 2310 major variants defined as rare mutations (maximum AF < 0.01) in previous studies (Additional file 1: Fig. S1H), suggesting an increased sensitivity for SV detection using the nanopore sequencing technology [17–19]. Besides, we found that 33.5% (n = 12,797) of our calls is novel compared to a multi-population SV callset identified from PacBio long-read sequencing data (Additional file 1: Fig. S1I). Notably, this amount of novelty is much higher than those identified from the single assembly of Tibetan (ZF1, n = 4005) or Han (HX1, n = 3908), suggesting that it is necessary to generate a comprehensive catalog of SVs through population-based methods. If we take all these long-read and short-read findings as a reference, then 10% (n = 3669) of our SVs remains unresolved before, and 278 of them are shared or major variants in our study (Fig. 1E; Additional file 1: Fig. S1F and S1G).

### Distribution of SVs across repeat sequences and functional genomic regions

**Fig. 2:**
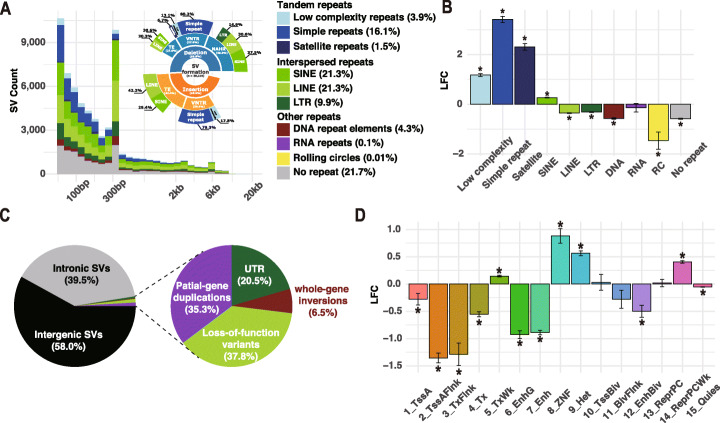
Distribution of structural variations across repeat sequences and functional genomic regions. **A** Distribution of insertions and deletions classified by intersected repeat elements. The formation mechanism was classified into three categories, including nonallelic homologous recombination (NAHR), variable number tandem repeats (VNTRs), and transposable elements (TE). **B** Log2 fold change (LFC) of enrichment analysis for repeat elements intersected with breakpoint junction sequences. Asterisk denotes significant enrichment with Bonferroni-corrected p-value < 0.05. **C** Proportions for potential effects on coding sequences. **D** LFC of enrichment analysis for structural variations intersected with 15-core chromatin states from RoadMap

We found that nearly 80% of the breakpoints of SVs overlapped with repetitive elements, of which most are interspersed repeats (56.8%) and tandem repeats (21.3%) (Fig. 2A). Compared to the random background, we found significant positive enrichment of short interspersed nuclear elements (SINEs) (FC = 1.20, p-value < 0.001) and clear depletion of other interspersed repeats (Fig. 2B), such as long interspersed nuclear elements (LINEs) (FC = 0.80, p-value < 0.001) and long terminal repeats (LTRs) (FC = 0.67, p-value < 0.001). These results indicated that the relatively small size of SINEs makes them more neutral than other interspersed repeats, so they manage to act as an important source of genetic diversity associated with the formation of SVs [26, 27]. Consistent with previous studies [16, 25], the SV length distribution profiles showed noticeable peaks at 300 bp, corresponding to SINEs, as well as 6 kb, corresponding to LINEs (Fig. 2A). To our surprise, we also found a considerable number of SV events around 300 bp overlapped with LINEs, especially for insertions (Additional file 1: Fig. S3A and S3B). Previous studies have reported that the Alu element is a non-autonomous retrotransposon belonging to SINE and has a length of ~300 bp. Therefore, the Alu element acquires trans-acting factors from LINE-1 [28], which could result in overlaps of SINEs and LINEs in modern human genomes.

Then, we used a simplified pipeline to infer the SV formation mechanisms according to the profile of breakpoint junction sequences (“Methods” section; Fig. 2A). Nonallelic homologous recombination (NAHR) was the major formation mechanism (38.3%, n = 7433) among all deletion events (< 1 Mb, n = 19,416), consistent with previous observations of SV hotspots [29]. Further classification of deletions showed that 37.5% (n = 2787) of the NAHR events overlapped with SINE on both sides, again suggesting that SINE plays an essential role in the formation of SVs. Variable number tandem repeats (VNTRs) were another contributor to SV formation, accounting for 27.6% (n = 5350) of deletions and 30.4% (n = 5296) of insertions (< 1 Mb, n = 17,424). Additionally, 41.4% (n = 7221) of insertions were mediated by transposable elements (TE), and approximately 71.7% (n = 5177) out of them were annotated as LINE- or SINE-mediated ones.

As SVs usually have deleterious effects when occurring in functional sequences [30, 31], we systemically evaluated possible functional impacts of major and shared SVs on both the coding and non-coding regions. First, we found that most SVs are located in introns or intergenic regions (Fig. 2C). Compared to the genomic background (Additional file 1: Table S3), SVs which were predicted to cause genic loss of function (LoF) showed a significant depletion (p-value < 0.001), while the intergenic SVs showed significant enrichment (p-value < 0.001). Further analysis of the nearest transcription start site (TSS) revealed that SVs rarely locate near the genes that are intolerant to LoF variations (p-value < 0.001). Besides, we found significant depletion of SVs overlapped with binding clusters of transcriptional repressor CTCF (p-value < 0.001) and boundaries of topologically associating domain (TAD) (p-value < 0.001). In addition, SVs are also strongly depleted in active chromatin regions and enriched in inactive regions (Fig. 2D). An exception is that a significant enrichment of SVs was found in weakly transcripted regions (TxWK, p-value < 0.001; Fig. 2D).

### Population structure and demographic inference

**Fig. 3:**
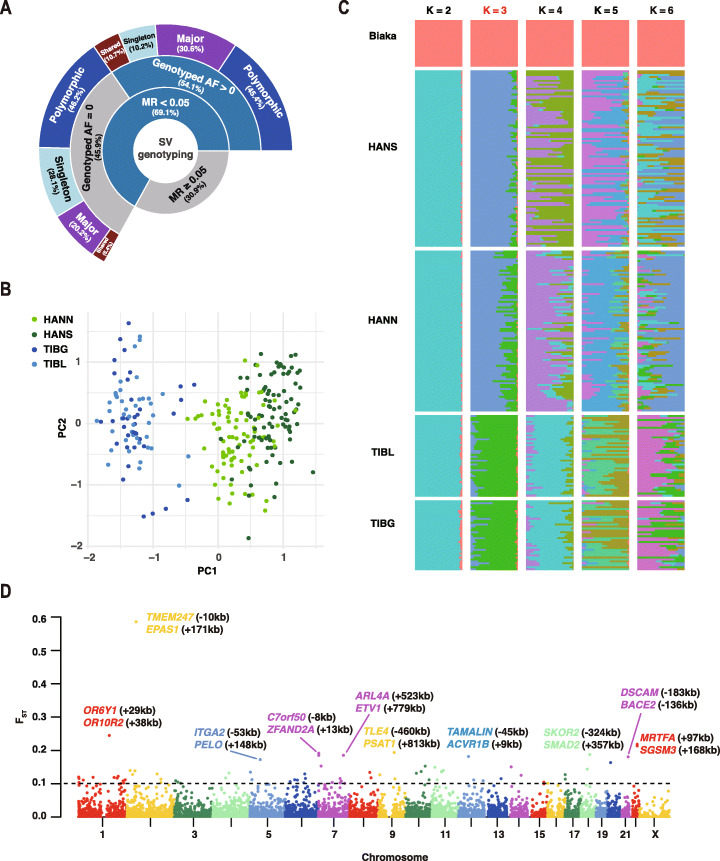
Population structure analysis using short-read data. **A** Genotyping results for short-read sequencing data. Discovery categories for original LRS calls were shown for structural variations (SVs) with the genotyped missing rate (MR) < 0.05, including shared (identified in all samples), major (identified in ≥ 50% of samples), polymorphic (identified in > 1 sample), and singleton (identified in only one sample) SVs. **B** Principle component analysis (PCA) of SV genotypes for Hans in North China (HANN) or South China (HANS), and Tibetans living above (TIBG) or below (TIBL) 4000 m. **C** Admixture analysis of Tibetans and Hans, using the Biaka populations as an outgroup. The minimum cross-validation error occurs when K = 3. **D** Manhattan plot for the window-based fixation index (F_ST_) statistics (Tibetans vs. Hans). Linear nearest genes to the top ten candidate regions are marked in the corresponding colors. Distances from the middle of the regions to the transcription start site of the genes are listed in brackets

To further study the population distribution of SVs discovered by LRS, we aggregated a human genome diversity panel constructed from 276 genomes using next-generation sequencing (NGS) data, including 78 Tibetans, 174 Hans, and 24 Biaka samples in the Central African Republic (“Methods” section; Additional file 3: Table S4). Targeted genotyping of SVs using NGS data is still a nontrivial challenging task [19, 32]. In this study, we applied a graph-based approach [32], and 69.1% (26,279/38,028) of the SVs were successfully genotyped in at least 95% of samples (Fig. 3A), which means the missing rate (MR) of genotyping is less than 5%. However, only 54.1% (14,204/26,279) of these successfully genotyped SVs were supported by at least one sample (Fig. 3A), indicating that nearly half of the SVs discovered by LRS cannot be recalled by NGS (genotyped AF = 0). Although the graph-based method reduced the MR of genotyping, it still resulted in an unsatisfying recall rate, similar to previous studies [14, 25]. Further analysis showed that 25.3% (3053/12,075) of these SVs, which failed to be recalled by NGS (12,075/26,279), were classified as major or shared SVs in the original LRS calls (Fig. 3A). This result indicated that the low recall rates are unlikely to be solely caused by rare mutations or somatic artifacts, at least for these common SVs discovered by LRS. Besides, 57.3% (6925/12,075) of these SVs overlapped with repeated sequences or segmental duplications, and another 19.2% (2315/12,075) interacted with repetitive breakpoint junction sequences (Additional file 1: Fig. S4A). These results suggested that the relatively low resolution in the repeated regions may be a significant flaw in SV genotyping with NGS data.

Before investigating the population structure, we observed that only 1308 biallelic SVs localized to autosomes violate Hardy-Weinberg assumptions for Tibetan, Hans, or Africans (“Methods” section; Additional file 1: Fig. S4B-E). Besides, we assessed the concordance of SV calls between all pairs of the three short-read WGS datasets used for genotyping and filtered out 2509 SVs failed in more than one comparison (“Methods” section; Additional file 1: Fig. S4F). After filtering, we obtained 10,387 successfully recalled SVs. Then, we divided Chinese samples into four subgroups according to genetic distance reported in previous studies, including Hans in North China (HANN) or South China (HANS), and Tibetans living above (TIBG) or below (TIBL) 4000 m. Meanwhile, Biaka samples were introduced as an outgroup (Additional file 3: Table S4). While large proportions of rare SVs were specific to individual groups, almost all SVs were shared across these groups at AF > 2.5% (Additional file 1: Fig. S4G). Principle component analysis (PCA) revealed that Biaka and Chinese samples were clustered in separated groups (Additional file 1: Fig. S4H). Restricting the analysis to Chinese populations unveiled two major clusters (Fig. 3B), including Tibetans and Hans, which is consistent with the results for whole-genome SNVs (Additional file 1: Fig. S4I). These two major clusters were also confirmed by the best-fitting admixture model with the minimum cross-validation error (K = 3, Fig. 3C). Meanwhile, we observed that HANS exhibited significant genetic distance from HANN, which is consistent with the main Chinese population clusters reported by a recent study [33].

After the population structure (TIB-HANN-HANS) was confirmed, we used whole-genome SNVs to estimate the demographic history of the three populations (“Methods” section). We tested three models with symmetric migrations to describe simplified evolution paths (Additional file 1: Table S5). After rounds of optimization, the second model with no size changes yielded the highest likelihood, describing a more recent divergence between HANN and HANS than between HAN and TIB. Specifically, the best-fit demographic model (Additional file 1: Fig. S5A and S5B; Table S7) indicated that the ancestors of Tibetans and Hans remained isolated until ~17,000 years ago (95% confidence interval [CI] 14,776 to 20,502 years), while HANN and HANS diverged much more recently, ~770 years ago (95% CI 727 to 810 years). These results confirmed the recent migration to the Qinghai-Tibet Plateau after the intense cold period during the Last Glacial Maximum (LGM, ~19,000 to 25,600 years before present) [11]. If we ignored the internal genetic divergence in Han populations and included Yoruba samples in Ibadan (YRI) to construct another population model YRI-TIB-HAN (“Methods” section; Additional file 1: Table S6), the best-fit demographic model (Additional file 1: Fig. S5C and S5D; Table S7) indicated that the ancestors of Africans and Chinese diverged ~88,000 years ago (95% CI 82,872 to 94,398 years), followed by TIB-HAN divergence ~22,000 years ago (95% CI 19,763 to 26,184 years). In this case, it takes a longer time for neutral variants to reach the level of variation within the Han populations. As a result, the divergence between Tibetans and Hans is much closer to the first major prehistoric migration into the plateau in the Upper Paleolithic (~30,000 years before present), supported by both archaeological and genetic studies [34, 35].

### Population-stratified SVs and candidate adaptive genes

In order to discover candidate adaptive variants, we calculated the fixation index (F_ST_) between Tibetans and Hans using the NGS data (“Methods” section). A total of 69 Tibetan-Han stratified SVs (F_ST_ > 0.1) were identified, including 63 deletions and 6 insertions (Fig. 3D; Table 1 and Additional file 4: Table S8). Nine SVs have a length > 1 kb, and the largest event is a ~6-kb deletion at chromosome 12q24. Almost all the population-stratified SVs were located in introns or intergenic regions, except for two deletions leading to the ablation of pseudogene transcripts (Additional file 4: Table S8). Consistent with previous reports [9, 20], the top candidate variant with the maximum F_ST_ is the 3.4-kb TED located in the intergenic region between *EPAS1* and *TMEM247* (transmembrane protein 247). We also confirmed the 163-bp Tibetan-Han stratified deletion in the intronic region of *MRTFA* [20]. To verify these population-stratified SVs, we performed polymerase chain reaction (PCR) and Sanger sequencing. Firstly, among the 69 stratified SVs, eight ones showed no intersection with repeat elements and were selected for validation. Then, we selected the top 8 stratified SVs (all these SVs were in repeat regions) for validation. Thus, a total of 16 stratified SVs were selected and we found that 87.5% of these candidates (14/16) successfully passed the validation (Additional file 1: Table S9). Besides, we found that not all Tibetan-Han stratified SVs can be tagged with SNVs in linkage disequilibrium (LD, r^2^ > 0.9). Among 69 Tibetan-Han stratified SVs, eight candidates have no linked SNVs in LD with r^2^ > 0.8, while eighteen ones have no linked SNVs in LD with r^2^ > 0.9 (Additional file 4: Table S8), suggesting that these SVs represent independent variations related to adaptation.

**Table 1:** Summary of top 10 population-stratified structural variations

No.	Location (GRCh37)	Type	Length (bp)	F_**ST**_	Genotyped AF			Candidate affected genes
					Tibetan	Han	Biaka	
1	chr2:46,694,272–46,697,680	Deletion	3408	0.54	0.58	0.02	0.00	*TMEM247* and other 8 genes
2	chr1:158,488,405–158,488,672	Deletion	267	0.22	0.84	0.51	0.56	*OR10Z1*
3	chr22:40,935,457–40,935,620	Deletion	163	0.22	0.51	0.17	0.00	*MRTFA*, *EP300*
4	chr9:81,725,770–81,726,090	Deletion	320	0.20	0.63	0.30	0.56	*TLE4*
5	chr7:1,185,068–1,187,658	Deletion	2590	0.19	0.77	0.45	0.92	*ZFAND2A*, *GPER1*
6	chr18:45,099,900–45,100,212	Deletion	312	0.18	0.24	0.02	0.48	*SKOR2*
7	chr7:127,766,419–127,766,732	Deletion	313	0.18	0.62	0.30	1.00	*LRRC4*
8	chr12:52,354,758–52,354,891	Deletion	133	0.17	0.60	0.29	0.04	*ACVR1B*
9	chr21:42,402,890–42,403,212	Deletion	322	0.17	0.70	0.39	1.00	*BACE2*
10	chr5:52,231,784–52,232,117	Deletion	333	0.17	0.63	0.89	0.90	*ITGA1*

*F*_*ST*_, fixation index between Tibetans and Hans; *AF*, allele frequency; *bp*, base pairs

**Fig. 4:**
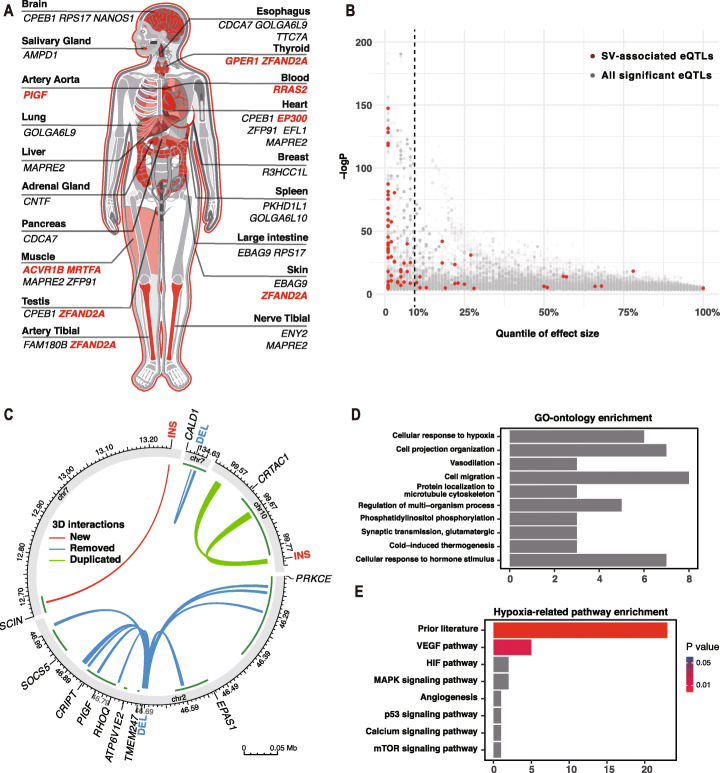
Candidate adaptive genes. **A** All linkage disequilibrium (LD)-linked genes across tissues. Known adaptive genes were marked in red color. **B** The rank of effect size and the p-value (-log10) for eQTLs across tissues. **C** All three-dimensional (3D) interactions and the corresponding genes affected by 69 population-stratified structural variations (SVs) (fixation index [F_ST_] > 0.1 between Tibetans and Hans). The outermost green layer represents the transcription regions of affected genes. Deletions (DEL) will remove overlapped interactions (blue), and insertions (INS) could create a new anchor (red) or duplicate nearby anchors and then construct new interactions with the closest anchors (green). **D** Gene-Ontology (GO) and **E** hypoxia-related pathway enrichment analysis for 80 candidate genes

SVs could alter the transcription of genes by affecting regulatory elements in non-coding regions [36–38], which is called position effects [39]. When occurring near genes, SVs exhibit remarkable regulatory effects and are more likely to act as expression quantitative trait locus (eQTL) than SNVs in distal regions [38]. We obtained 119 high causal LD-linked eQTLs (Additional file 5: Table S10; Additional file 6: Table S11) related to both known adaptive genes and novel candidates (Fig. 4A; Additional file 1: Fig. S6), and the majority of these eQTLs (n = 95) exhibited the top 10% effect size to the corresponding gene (p < 0.001; “Methods” section; Fig. 4B). Besides, SVs have a more direct impact on the three-dimensional (3D) genome than SNVs [40]. Using a recently published algorithm [40, 41], we found that four Tibetan-Han stratified SVs could lead to chromatin conformational changes (“Methods” section; Fig. 4C). For example, we found that the 3.4-kb TED, which has been reported to affect the function of EPAS1 [9], is able to modify the high-order chromatin structure by removing a nearby chromatin loop anchor that mediated interactions between regulatory elements and multiple distant genes (Fig. 4C and Additional file 1: Fig. S6D). Finally, we summarized three types of evidence-supported genes (n = 80) that tend to be affected by the Tibetan-Han stratified SVs, including linear overlapping/nearest genes, 3D affected genes, and LD-linked eQTL genes (eGenes) (Fig. 4A, C; Additional file 7: Table S12). Among these potentially affected genes, 28 genes have been reported in previous studies to be relevant to HAA, and another 33 genes have traits directly or indirectly related to hypoxia (Additional file 7: Table S12). Gene-Ontology enrichment analysis showed that cellular response to hypoxia, vasodilation, and cold-induced thermogenesis are among the top enriched functional terms of these candidate genes (Fig. 4D; Additional file 8: Table S13). Besides, enrichment analysis of hypoxia-related pathways indicated that candidate genes are most significantly enriched in the VEGF pathway (Fig. 4E; Additional file 9: Table S14), which could stimulate the proliferation of endothelial cells (ECs) and promote angiogenesis [42]. Among the novel candidates involved in the VEGF pathway, integrin subunit alpha 1 (*ITGA1*) encodes a positive regulator of blood vessel diameter; ITGA1 usually dimerizes with ITGB1 and forms a cell-surface receptor, which facilitates attachment of dermal microvascular ECs and collaborates with VEGF in promoting MAPK activation and angiogenesis [42].

### Signatures of positive selection and archaic introgression

**Fig. 5:**
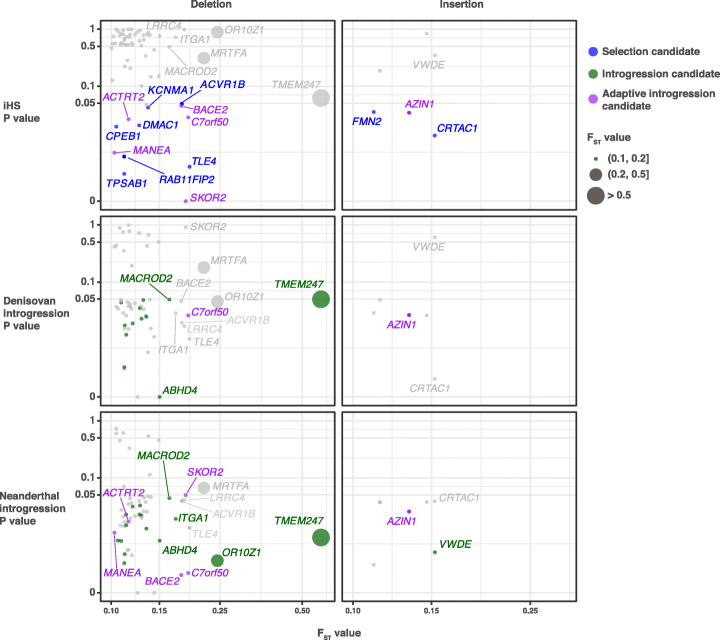
Candidate adaptive and archaic introgressed structural variations. Joint distributions of fixation index (F_ST_) statistics for the population-stratified structural variations (SVs) (x-axis), p-values for positive selection (integrated haplotype homozygosity score [iHS], top row), Denisovan introgression (f_d_-statistic, middle row), and Neanderthal introgression (f_d_-statistic, bottom row). SVs that show signatures of both selection and introgression (purple circles) are distinguished from the variants that show signatures of selection (blue circles) or introgression (green circles) only. The p-values point to the most significant and closest windows to SVs (**±**500 kb)

As described in previous studies [12, 13], the population-stratified SVs can originate from de novo mutations or standing variations that have been introgressed from other hominins and were then subject to natural selection or demographic processes. To determine whether there are introgressed SVs that contribute to the local adaptation of Tibetan highlanders, we searched for signatures of selection and archaic hominin introgression using population genetic statistics towards SNVs from the sequences surrounding the Tibetan-Han stratified SVs (“Methods” section). Neutral variations from parametric coalescent simulations based on the best-fit demographic model for Tibetans (Additional file 1: Fig. S5 and S7; Table S15) were considered as the null expectation to estimate the significance of selection and introgression at individual loci (“Methods” section). Using evaluation methods described in previous studies [12, 43], we found that our whole-genome coalescent simulations could recapitulate the mutation patterns of SNVs and exhibited the ability to identify non-neutral variants (“Methods” section; Additional file 1: Fig. S8 and S9). We determined the selection and introgression candidates by checking whether they are close (< 500 kb) to a significant signature. Among the 69 Tibetan-Han stratified SVs, we identified signatures of natural selection around 15 SV loci (p < 0.05, integrated haplotype homozygosity score [iHS]) and archaic introgression around 25 loci (p < 0.05 for D-statistic, f_d_-statistic, and S^*^-like statistic), including 23 and 14 loci using Neanderthal and Denisovan genomes as the archaic reference respectively. Six selection candidates also have introgression signals at the flanking sequences, which were marked as adaptive introgressed SVs (Fig. 5; Additional file 4: Table S8; Additional file 1: Table S16). After genotyping of these adaptive introgression candidates in archaic hominins, three deletions were supported by at least one ancient genome with short-read sequencing data (Additional file 1: Table S16), including a 335-bp deletion at 1p36.32 (chr1:2,919,030–2,919,365), a 2590-bp deletion at 7p22.3 (chr7:1,185,070–1,187,063), and a 322-bp deletion at 21q22.2 (chr21:42,402,890–42,403,212). Among these three candidates, the deletions at 7p22.3 and 21q22.2 caused copy number losses in both Biaka and ancient populations (Additional file 1: Fig. S10A and S11A). Meanwhile, both deletions exhibited high genotyped variant frequencies in the Biaka population (AF = 0.92 and 1, respectively; Additional file 1: Fig. S10B and S11B). Therefore, it is difficult to determine whether these two deletions are derived from ancient humans, because Neandertals and Denisovans should not have obvious genetic communications with African ancestors [12, 13].

**Fig. 6:**
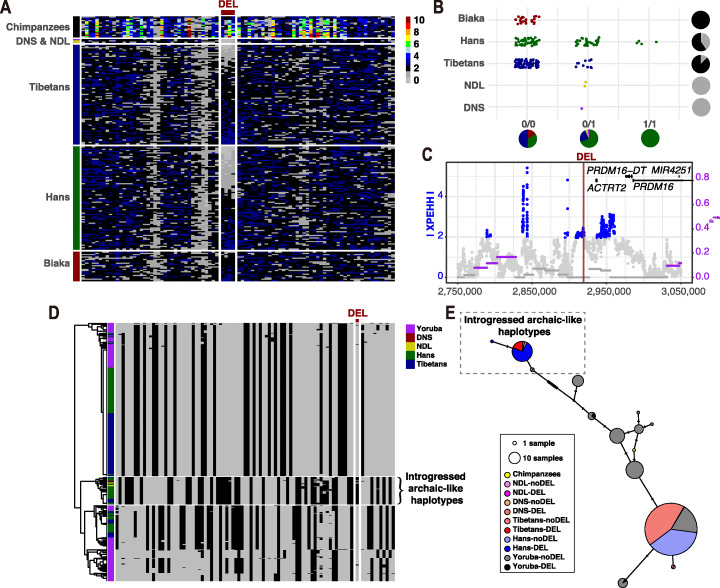
Signatures of selection and archaic introgression from Neandertals (NDL) and Denisovans (DNS) for the deletion of 335 base pairs at 1p36.32. **A** Absolute integer copy numbers for a sliding window of 100 base pairs (bp) and a step size of 50 bp around the deletion. Each row represents the copy numbers of a sample over the region. **B** Genotyping results for the deletion among different populations. Pie charts along the x-axis indicate the population distribution for different structural variation (SV) genotypes (colors are the same as populations), and pie charts along the y-axis illustrate the frequency distribution for a given population (colors are the same as copy number of 2/1/0). **C** Distributions of single nucleotide variants (SNVs) with significant f_d_-statistic (purple dots) and cross-population Extended Haplotype Homozygosity (XP-EHH, blue dots). **D** Hierarchical clustering for the region around the deletion. Rows illustrate the individual haplotypes. The column indicates the genotypes of 90 variants with selection signals (XP-EHH > 2). The color of gray and black represents the ancestral and derived alleles, respectively. **E** TCS haplotype network for 20 linkage disequilibrium (LD)-linked SNVs to the deletion with selection signals (XP-EHH > 2). All haplotypes with the deletion in Tibetans and Hans, and the haplotypes with or without the deletion in the ancient genomes are clustered together within the dotted box

The deletion at 1p36.32 (chr1:2,919,030–2,919,365) was found only in the Chinese and ancient genomes (Fig. 6B). No evident copy number losses around this deletion were found in either Biaka genomes or Chimpanzees (Fig. 6A), suggesting a high-confidence introgressed SV locus. Meanwhile, the flanking SNVs exhibited significant positive cross-population Extended Haplotype Homozygosity (XP-EHH), suggesting that this region might undergo positive selection in the Tibetans (Fig. 6C; Additional file 10: Table S17; Additional file 11: Table S18). It is worth noting that this deletion occurred less frequently in Tibetans (AF = 0.06) than in Hans (AF = 0.23) (Fig. 6B), suggesting that it is the ancestral allele subject to positive selection. Although the allele frequency was relatively lower than other Tibetan-Han stratified SVs, this deletion exhibited high rates of heterozygosity for carriers in both Tibetans (100%) and Hans (88%), indicating a considerable variant frequency in Chinese populations (Fig. 6B). We further found that this SV exists in populations from the 1000 Genomes Project and has the highest variant frequency in East Asians (AF = 0.27) and the lowest in Africans (AF = 0.06). Therefore, we extracted 90 flanking SNVs of this deletion with positive selection signals (XP-EHH > 2) and detected the differences between haplotypes of the Tibetan, Han, and African populations. Through hierarchical clustering and network analysis (Fig. 6D, E), we found that all haplotypes with the deletion in Tibetans and Hans were clustered together, as well as the haplotypes with or without the deletion in the ancient genomes. In contrast, all haplotypes with the deletion in YRI were clustered with other haplotypes without the deletion, indicating a distinct pattern from the Chinese population. As expected, the beneficial standing ancestral allele co-exists with complex genomic backgrounds, which resulted in remaining genetic variations in current Tibetan populations and no significant differences between haplotypes without the deletion.

To explore the associations between the 335-bp deletion at 1p36.32 and phenotypes, we used blood samples from 418 Tibetan highlanders previously collected by our lab and measured 51 quantitative traits. Using PCR and Sanger sequencing, we genotyped the 335-bp deletion in these Tibetans as well as 72 Hans. The genotyping frequency of the deletion is similar to the estimates from the NGS data (AF = 0.102 in Tibetans and AF = 0.236 in Hans). Consistent with the findings in the NGS data, deletion carriers exhibited high rates of heterozygosity in both Tibetans (94%) and Hans (87%). We then performed association analyses on these samples and found that the deletion tends to associate with some blood indices, including alanine aminotransferase (ALT; p = 0.006, FDR = 0.319) and albumin (ALB; p = 0.043, FDR = 0.478), which are biomarkers of liver function (Additional file 1: Fig. S12). However, these associations were not significant after multi-testing correction. Consistent with this finding, the association tests between 91 quantitative traits collected from 2849 Tibetan samples [8] and genotypes for 55 SNVs in strong LD (r^2^ > 0.8) with this deletion confirmed the connection with liver functions, as indicated by ALT (p = 0.003, FDR = 0.126), aspartate transaminase (AST; p = 0.001, FDR = 0.047), and alkaline phosphatase (ALP; p = 0.018, FDR = 0.417) (Additional file 1: Fig. S13).

To explore the function of the 335-bp deletion at 1p36.32, we further inspected LD-linked eQTLs for this deletion using QTLbase [44]. Among all the significant eQTLs (Additional file 12: Table S19), rs7546695 (r^2^ = 0.82 with this deletion) and rs6681331 (r^2^ = 0.82) are negatively correlated with the expression of the two closest genes to this deletion respectively, actin-related protein T2 (*ACTRT2*) and PR/SET domain 16 (*PRDM16*). Through luciferase reporter gene assays in HUVEC and K562 cell lines, we found that compared with the wild-type sequence, the 335-bp deletion could significantly reduce the luciferase activity of the reporter with the promoter region of *PRDM16* but not with *ACTRT2* (Additional file 1: Fig. S14). Furthermore, we designed guide RNAs using the CRISPR design tool to knockout (KO) the sequence of this deletion in the HUVEC cell line. After genotyping by Sanger sequencing, we acquired candidate KO clones and found that these KO clones exhibit a significantly lower expression of *PRDM16* compared to the control (Additional file 1: Fig. S14H). These results suggest that the 335-bp deletion at 1p36.32 could downregulate the expression of *PRDM16*. *PRDM16*, which is located at 66 kb downstream of this deletion, is a key regulator of the thermogenic program that controls the endogenous transformation of white to brown adipose tissues in response to cold [45, 46], and has been reported to exhibit upregulated expression in the Tibetan pig [47]. Therefore, the relatively low frequency of this deletion in Tibetans could contribute to a quick response to the cold environment in high-altitude regions.

In summary, the 335-bp deletion at 1p36.32, which is located in a region with weak positive selection and likely to be introgressed from the ancient humans, might benefit cold environment adaptation through regulation of *PRDM16*, accompanied by potential protective effect for liver functions.

## Discussion

In this study, we comprehensively analyzed the complete spectrum of SV in a Chinese Tibetan and Han population using nanopore sequencing technology. We explored novel adaptive genes affected by Tibetan-Han stratified SVs. Besides, we carried out the first study to investigate adaptive introgressed SVs in the whole genome of Tibetans. Overall, our results provide a valuable resource for future HAA studies.

Due to the limitations of the short-read sequencing technology, a substantial amount of SVs remains uninvestigated. Compared to a multi-population SV callset identified from PacBio long-read sequencing data [14], there are still a considerable number of SVs in our catalog that are sequence-resolved for the first time. Notably, this amount of novelty is much higher than those identified from the single assembly of Tibetan (ZF1) or Han (HX1), suggesting that it is necessary to generate a comprehensive catalog of SVs through population-based methods which have the advantage of detecting novel SV events of singletons or even shared ones. Then, we adopted a combined strategy that SVs discovered within a small population using LRS were genotyped with a relatively large amount of NGS data. It is noteworthy that approximately half of the SVs discovered in LRS data cannot be recalled in NGS data. Through analyzing the local sequence architecture around SVs, we speculated that the relatively low resolution in the repetitive regions is likely to be the primary defect of SV genotyping with NGS data. Further genotyping studies should focus on improving the unsatisfying recall rates in repetitive regions. To facilitate the use of these resources for the genomics community, we published our callset with high-coverage genotype calls from 276 samples.

Though SVs are known to impact the formation of population diversity and adaptation positively [2], few studies have examined SVs’ role in HAA. In this study, we identified 69 Tibetan-Han stratified SVs, five of them were also included in the candidate list of adaptive SVs from ZF1 [20]. Therefore, 64 population-stratified candidates in this study are novel compared to the previous report. Notably, the top four candidates in our study are enlisted in their reports (Additional file 4: Table S8), suggesting that the top stratified SVs are consistent across different studies. Considering three types of evidence-supported genes, we screened out a total of 80 protein-coding adaptive genes affected by these population-stratified SVs between Tibetans and Hans, providing valuable resources for future HAA studies. Moreover, the majority of these genes have not been reported in previous studies, which could improve our understanding of multi-variant adaptation. For example, we found that two known adaptive SVs could affect multiple adaptive genes far from their breakpoints. The 3.4-kb TED could remove a convergent interaction between 46.68 and 46.88 Mb in chromosome 2, which will disable the interactions between many strong enhancers/active promotors and five continuous protein-coding genes within this region (Additional file 1: Fig. S6D), including *TMEM247*, *ATP6V1E2*, *RHOQ*, *PIGF*, and *CRIPT*. Through the discovery of Tibetan-Han stratified SNVs in this region, it has been proved that all these candidate genes were relevant to HAA [4, 6]. Furthermore, LD-linked eQTL tests also confirmed the relationship between the TED and PIGF (Additional file 1: Fig. S6A and S6D; Additional file 5: Table S10; Additional file 6: Table S11). Phosphatidylinositol glycan anchor biosynthesis class F (PIGF) is an essential member of the vascular endothelial growth factor (*VEGF*) family and facilitates angiogenesis in Tibetans [6]. Therefore, the TED is likely to regulate many adaptive genes besides *EPAS1*. Similarly, we found that the 163-bp deletion in the intronic region of *MRTFA* was also involved in a high-confidence LD-linked eQTL relationship with E1A binding protein p300 (*EP300*) (Additional file 1: Fig. S6B; Additional file 5: Table S10; Additional file 6: Table S11), which encodes a co-activator of *HIF1A* and can stimulate other hypoxia-induced genes [6].

Although recent studies have found that genetic variants originated from archaic hominins contribute to local population adaptation [4, 12, 13], little is known of adaptive introgressed SVs in Tibetans. We searched for signatures of natural selection and ancient human introgression around Tibetan-Han stratified SVs using simulated neutral variations and finally identified a total of 6 adaptive introgressed candidates. To the best of our knowledge, this is the first study to detect adaptive introgressed SVs in the whole genome of Tibetans. Our results suggested that the introgressed SVs could contribute to the local adaptation of Tibetan highlanders. It should be noted that SVs which were not successfully recalled in any ancient genomes were not necessarily absent from ancient humans, especially considering the short read of ancient genomes. Each candidate needs more detailed investigations in the future. For instance, the 2590-bp deletion at 7p22.3 (chr7:1,185,070–1,187,063) is located nearby a region with reported selection signatures [6]. We found that many flanking SNVs highly linked to this deletion (r^2^ > 0.8) exhibited both signatures of natural selection (|XP-EHH|> 2) and archaic introgression (p < 0.05 for D-statistic, f_d_-statistic, and S^*^-like statistic) (Additional file 1: Fig. S10C; Additional file 13: Table S20), suggesting that the surrounding genomic region of the deletion could come from archaic introgression and contribute to adaptation. Besides, four of these flanking SNVs (rs7805591, rs2949174, rs2140578, rs2949172) were identified as eQTLs exhibiting negative associations with the expression levels of zinc finger AN1-type containing 2A (*ZFAND2A*) and G protein-coupled estrogen receptor 1 (*GPER1*) (Fig. 4A and Additional file 1: Fig. S6C; Additional file 5: Table S10; Additional file 6: Table S11). Both *ZFAND2A* and *GPER1* locate close to this deletion and are associated with HIF1A. *ZFAND2A* is predicted to be a target gene of the HIF1A transcription factor using known transcription factor binding site motifs from the TRANSFAC database [48, 49]. Moreover, *ZFAND2A* was recently reported to undergo positive selection and exhibit significantly downregulated expression in the Tibetan pig [50]. Meanwhile, activation of *GPER1* by 17β-estradiol (E2) and the specific agonist G-1 will trigger a GPER1-EGFR-MAPK1-FOS signaling pathway, leading to the increased expression level of VEGF through upregulation of HIF1A [51, 52].

## Conclusions

In conclusion, our study demonstrates that SVs play an important role in the evolutionary processes of Tibetans’ adaptation to the Qinghai-Tibet Plateau. Our comprehensive SV callset, which consists of 38,216 unique SVs, provides a considerable number of previously unresolved common variants in Chinese populations. Furthermore, we screened out a total of 80 protein-coding adaptive genes affected by 69 population-stratified SVs between Tibetans and Hans, which reveals new evidence of HAA and will improve our understanding of multi-variant adaptation. Besides, we carried out the first study to investigate adaptive introgressed SVs in the whole genome of Tibetans and discovered 6 candidates, suggesting that SVs originated from archaic hominins and introgressed into Chinese populations could contribute to the local adaptation of Tibetan highlanders.

## Methods

### Study participant recruitment

We recruited 15 unrelated ethnic Tibetan and 10 unrelated Han subjects for nanopore sequencing (Additional file 1: Table S1). These 25 subjects were recruited during a physical examination program at the community conducted in December 2018 in Chongqing city located in southern China. Among the 15 indigenous Tibetan subjects, seven came from Shigatse (4000 m above sea level), and the others came from areas of relatively lower altitudes (between 3000 and 4000 m). The mean age (s.d.) of these Tibetans is 20.13 (0.99) years old. All Hans came from plain areas (≤ 1500 m), and their mean age (s.d.) is 23.20 (4.49) years old. All subjects are males. Besides, we also aggregated several short-read whole-genome sequencing (WGS) datasets (Additional file 1: Supplementary Methods; Additional file 3: Table S4).

All the abovementioned studies were performed with the approval of the Medical Ethical Committee of the Beijing Institute of Radiation Medicine (Beijing, China). Written informed consent was obtained from each participant.

### Nanopore sequencing

The peripheral blood samples were collected from 25 subjects, and then the genomic DNA was extracted by QIAGEN® Genomic DNA extraction kit (Cat ID: 13323, QIAGEN) according to the standard operating procedure provided by QIAGEN. The extracted DNA was detected by NanoDrop™ One UV-Vis spectrophotometer (Thermo Fisher Scientific, USA) for DNA purity (OD260/280 ranging from 1.8 to 2.0 and OD 260/230 is between 2.0 and 2.2); then, Qubit® 3.0 Fluorometer (Invitrogen, USA) was used to quantify DNA accurately. Since the sample was qualified, the BluePippin system (Sage Science, USA) was used to size-select long DNA fragments. Then, we repaired the DNA and prepared the DNA ends for adapter attachment. The sequencing adapters supplied in the SQK-LSK109 kit were attached to the DNA ends. Finally, Qubit® 3.0 Fluorometer (Invitrogen, USA) was used to quantify the size of library fragments. In the end, we primed the Nanopore GridION X5 sequencer (Oxford Nanopore Technologies, UK) flow cell and loaded the DNA library into the flow cell. All samples were sequenced with 1D R9.4.1 nanopores. Each genome was sequenced to a 10~20× coverage depth.

### SV discovery and genotyping

Base-calling of the raw nanopore squiggles was performed using Guppy v2.3.1 for GridION, and a minimum quality score of 7 (Q7) was applied. Reads were mapped to GRCh37 human reference. Mapping was performed using NGMLR (v0.2.7) [21] with default parameters for the nanopore sequencing. SV calling was performed using Sniffles (v1.0.11) [21], NanoSV (v1.2.3) [15], and SVIM (v1.2.0) [53]. Then, we systemically assessed the distribution of these SVs across repeat sequences and functional genomic regions. Further information is described in the Supplementary Methods (Additional file 1).

SVs discovered using LRS were genotyped with a relatively large amount of NGS data accumulated in previous studies. Reads from all short-read datasets were mapped to GRCh37 human reference. Mapping was performed by BWA-MEM (v0.7.19) [54] with default parameters. Using the SVs discovered by the nanopore sequencing, we took Paragraph (v2.4a) [32] to genotype each genome generated using NGS data. All genotypes which failed to pass any filter by Paragraph were replaced with missing genotypes (./.). Then, we performed filtering of batch effects and Hardy-Weinberg equilibrium for the genotyping results. Further information is described in the Supplementary Methods (Additional file 1).

### Population structure analysis

We carried out PCA and admixture analysis to infer the covariance structure of allelic frequencies. Next, we employed the Weir and Cockerham estimator for F_ST_ based on VCFtools to identify the Tibetan-Han stratified SVs. We considered three types of evidence-supported genes that tend to be affected by these SVs. Then, we used SNVs to estimate the demographic history of Tibetans and Hans and performed whole-genome coalescent simulations with msprime [55]. In order to identify all candidate adaptive genes that tend to be affected by the Tibetan-Han stratified SVs, we considered three types of evidence-supported genes, including linear overlapping/nearest genes, 3D affected genes, and LD-linked eQTL genes. Further information is described in the Supplementary Methods (Additional file 1).

### Natural selection and archaic introgression

To detect evidence of natural selection in Tibetans, we calculated iHS [56] and XP-EHH [57] using an R package rehh [58]. Besides, we applied the D-statistic and f_d_-statistic using admixr [59] to distinguish excess genetic drift from the ancient introgression based on allele frequencies. In the meantime, we performed an S^*^-like method based on linkage information as an orthogonal method to validate the signals of introgression [60]. Further information is described in the Supplementary Methods (Additional file 1).

## Supplementary Information

**Additional file 1:** Supplementary Methods; Figures S1-S14; Tables S1, S3, S5-S7, S9, S15-S16, S21-S24.

**Additional file 2: Table S2**

**Additional file 3: Table S4**

**Additional file 4: Table S8**

**Additional file 5: Table S10**

**Additional file 6: Table S11**

**Additional file 7: Table S12**

**Additional file 8: Table S13**

**Additional file 9: Table S14**

**Additional file 10: Table S17**

**Additional file 11: Table S18**

**Additional file 12: Table S19**

**Additional file 13: Table S20**

**Additional file 14.** Review history.

## Data Availability

The basecalled nanopore sequencing data generated in this study have been submitted to the NCBI BioProject database (https://www.ncbi.nlm.nih.gov/bioproject/) under accession number PRJNA681146 [61]. SV calls are shared on dbVar (https://www.ncbi.nlm.nih.gov/dbvar) under accession dbVar: nstd199 [62]. All scripts used in this study are available at GitHub (https://github.com/quanc1989/SV-ONT-Tibetan) [63] under the MIT License. Short-read sequencing data from Lu et al. [11] are publically available at the Genome Sequence Archive (GSA: https://bigd.big.ac.cn/gsa) under the accession number PRJCA000246. Short-read data from Lan et al. [64] are publically available at the European Nucleotide Archive (ENA: https://www.ebi.ac.uk/ena) under the accession number PRJEB11005. Raw sequencing data for chimpanzees from the Great Ape Genome Project [65] are publically available at the SequenceRead Archive (SRA: https://www.ncbi.nlm.nih.gov/sra) under the accession number PRJNA189439. Alignments for three archaic hominin genomes [66–68] are publically available at the website of the Department of Genetics in Max Planck Institute for Evolutionary Anthropology (https://www.eva.mpg.de/genetics).
