# Supplementary material for: Characterization of structural variation in Tibetans reveals new evidence of high-altitude adaptation and introgression
Source: Genome Biol. 2021 May 25;22:159. doi: 10.1186/s13059-021-02382-3 (PMC8146648; doi:10.1186/s13059-021-02382-3)
Supplement: Supplementary file 1 — Additional file 1: Supplementary Methods; Figures S1-S14; Tables S1, S3, S5-S7, S9, S15-S16, S21-S24. [file 13059_2021_2382_MOESM1_ESM.doc]

**Supplementary Information for**

**Characterization of structural variation in Tibetans reveals new evidence of high-altitude adaptation and introgression**

Cheng Quan^†^, Yuanfeng Li^†^, Xinyi Liu^†^, Yahui Wang, Jie Ping, Yiming Lu^*^, Gangqiao Zhou^*^

^†^These authors contributed equally to this work as first authors.

^*^Corresponding author Email: zhougq114@126.com (G.Z.); ylu.phd@gmail.com (Y.L.)

This file includes:

Supplementary Methods

Supplementary Figures S1-S14

Supplementary Tables S1, S3, S5-S7, S9, S15-S16, S21-S24 (other tables are provided as Excels).

# Supplementary Methods

## Short-read data aggregation

We used a short-read whole-genome sequencing (WGS) dataset generated by our lab, including 43 unrelated ethnic Tibetans and 46 unrelated individuals of Hans (Additional file 3: Table S4). These 43 Tibetans were recruited during a physical examination program at the community conducted from June 2010 to August 2010, who live in five counties (including Batang, Dawu, Kangding, Dzongzhab, and Litang) in Sichuan province located in southwest China, at > 2,500 meters over sea level. The male/female ratio and the mean age (s.d.) of these Tibetans are 1.50 and 36.31 (12.31) years old respectively. These 46 Hans were recruited in a regular physical examination from July 2010 to August 2010 at the Guangxi Cancer Hospital (Nanning city, China), who lives in Nanning city, Guangxi province located in southern China, at < 100 meters over sea level. The male/female ratio and the mean age (s.d.) of these Hans are 1.50 and 42.22 (6.39) years old respectively. These samples were sequenced to 150 bp paired-end reads with an average coverage of 40x using the Illumina HiSeq X Ten.

In the meantime, we aggregated several short-read WGS datasets published in previous studies. Firstly, we collected a subset of the publicly available NGS dataset of 38 Tibetan highlanders and 39 Han lowlanders sequenced by the Illumina HiSeq X Ten [1]. Secondly, we collected another NGS dataset including 45 Northern Han Chinese and 45 Southern Han Chinese samples [2]. These samples were sequenced to 50 bp paired-end reads with an average coverage of 80x using the Illumina HiSeq 2000. Besides, 24 Biaka in the Central African Republic from the Human Genome Diversity Project (HGDP) was introduced as an outgroup. These samples were sequenced to 150 bp paired-end reads with an average coverage of 40x using the Illumina HiSeq X Ten. To discover archaic introgression of SVs, we also collected three publicly available archaic hominin genomes, including a Denisovan (Illumina’s Genome Analyzer IIx platform) [3], a Neanderthal (HiSeq 2500 platform) [4] from the Altai Mountains, and another Neanderthal from Croatia (HiSeq 2500 platform) [5]. These archaic samples were sequenced to 50 bp paired-end reads with an average coverage of 40x. Chimpanzee WGS data were obtained from Great Ape Genome Project [6], including 17 individuals which were sequenced to 50 bp paired-end reads with an average coverage of 25x using the Illumina HiSeq 2000.

## SV discovery

SV calling was performed using Sniffles (v1.0.11) [7], NanoSV (v1.2.3) [8], and SVIM (v1.2.0) [9]. These tools have been reported to be compatible with NGMLR and show better accuracy and sensitivity than others [10]. First, we used the tools described above to perform SV detection on each sample. Five minimum supporting reads with at least 50 bp length were required. The insertion sequence and read ID were required for each method, and the rest are all default parameters. SURVIVOR (v1.0.7) [11] was used to merge the SVs supported by at least two methods with a maximum allowed pairwise distance of 1,000 bp between breakpoints. Meanwhile, the SVs obtained from different tools are not necessary to agree on the SV-type or the strand, so that we could capture as many potential breakpoints as possible. Finally, we merged the SVs obtained from all the samples as long as one sample supports it.

Thus, we have obtained potential regions for all samples, and we need to get a fully genotyped multi-samples dataset. We re-ran Sniffles across all the samples with all these potential regions (--Ivcf) and finally combined the SVs with SURVIVOR. This time, we asked SURVIVOR only to report SVs supported by at least one sample, and they have to agree on the SV-type. Furthermore, we used a hard threshold with five minimum supporting reads, and all non-missing genotypes less than this threshold were modified to reference. To comprehensively understand the local context of SVs, we decided not to filter out centromeric or pericentromeric regions.

## Comparison of SVs to diverse databases

We compared our SVs to several previously published datasets using AnnotSV (v2.2) [12], including the Database of Genomic Variants (DGV), gnomAD [13], the Deciphering Developmental Disorders (DDD) Study, the 1000 Genomes Project [14], and a common disease trait mapping study [15]. Besides, we also compared our SVs with three SV callsets identified from long-read sequencing data, including ZF1 [16], HX1 [17], and a multi-population study [18]. Only the variants overlapping at least 50% of our SVs were reported. We calculated the maximum variant frequencies for SVs found in these databases and annotated each SV with the corresponding database.

## SV distribution

To measure the SV distribution in the human genome, we divided each chromosome into continuous 500 kb windows and summed the number of SVs overlapped with the window. Windows that had overlapped with centromeres were excluded. For insertions and translocations, we only considered the position of breakpoints. While for inversions, deletions, and duplications, we considered the entire SV region. We calculated the distance from each window’s edge to the nearest telomere, and log2 fold change (LFC) was used to compare the degree of enrichment inside and outside 5 Mb to the telomere. A 1000 round permutation test was performed by randomly exchanging the position of windows, and the empirical p-value was calculated by the distribution of LFC for each test and corrected by the Bonferroni method.

### Genomic background model

To perform overlap enrichment analysis of SVs versus genomic elements, we generated 1000 randomly shuffled SV sets in the non-gap region of the GRCh37 human genome using BEDTools (v2.28.0) [19], as described in the SV release of the 1000 Genomes Project (phase 3) [14]. Each SV in a single shuffled set has the same SV-type and length as the corresponding real SV within the same chromosome but no intersection. These shuffled sets made up a random background model to learn the enrichment of genomic elements overlapped with real SVs versus the null distribution. LFC was used to measure the enrichment, and the empirical p-value was calculated and reported as significant if p-value < 0.05 after the Bonferroni correction.

### Breakpoint analysis

AnnotSV was used to identify the overlapping repetitive sequences at the SV breakpoints (+/- 100bp) using RepeatMasker annotations from the University of California Santa Cruz (UCSC) Genome Browser. A breakpoint was annotated as the repeat class of a specific repetitive sequence which intersects with its surrounding sequence. RepeatMasker annotations from the UCSC Genome Browser Database used the RepBase library [20] from the Genetic Information Research Institute (GIRI) and summarized the repetitive sequences into ten different classes, including low complexity repeats, simple repeats, satellite repeats, SINE, LINE, LTR, DNA repeat elements, RNA repeats, other repeats, and unknown ones. We performed permutation tests for each repeat class using the random background model.

Breakpoint junction sequences can be used to infer formation mechanisms for SVs [21]. We used a simplified pipeline to classify the mechanisms, originally from BreakSeq [21] and 1000 Genomes Project (phase 3) [14]. First, SVs were examined for > 50% overlapped by tandem repeats to identify the expansion or contraction of VNTRs. Then, SVs were classified as NAHR if both breakpoints were annotated as the same repeat class and the sequence identity between the two breakpoints is more than 80%. Finally, the SVs overlapped with interspersed repetitive sequences were inferred into TE-mediated mechanisms. We considered only VNTR and TE for insertions.

### Gene annotations and chromatin features

Consistent with the method described in the GnomAD-SV [13], we used the protein-coding gene annotations from Gencode and generated a canonical transcript described in Ensembl Genomes Project [22]. Then we annotated SVs for a range of potential effects on coding sequences using svtk [23], including LoF variants, copy gains (CG), partial gene duplications, whole-gene inversions, intronic, and intergenic SVs. Then we detected the potential purifying selection against gene-altering SVs. Annotations from the Exome Aggregation Consortium (ExAC) [24] were used to indicate the probability of a gene intolerance to variations, including synZ (Z score > 0 indicates intolerance to synonymous variations), misZ (Z score > 0 indicates intolerance to missense variations), and pLI (pLI > 0.9 indicates intolerance to loss of function variations). In the meantime, predictions of haploinsufficiency (HI_DDDpercent < 10% indicates more likely to exhibit haploinsufficiency) from DECIPHER [25] and assessments of dosage-sensitive (HI_CGscore < 10 indicates more likely to exhibit dosage pathogenicity) from the Clinical Genome Resource (ClinGen) Consortium Rating System were also included. Permutation tests for annotations of all potentially affected genes were performed using the random background model.

Moreover, we used AnnotSV to detect the associations with chromatin boundaries at the borders of TAD and CTCF binding clusters. As described in a previous study [26], we used BEDTools-cluster on the narrowPeak files from the ENCODE [27] with a merge distance of 5 kb to generate the CTCF binding clusters. To comprehensively characterize the chromatin landscape, we also collected 15-core chromHMM chromatin states applied to 127 epigenomes from RoadMap [28]. Five states were annotated as inactive (8_ZNF/Rpts, 9_Het, 13_ReprPC, 14_PeprPCWk, 15_Quies), and the others were active. Likewise, permutation tests were performed using the random background model.

## SV genotyping and SNV discovery

As suggested in recent studies [18, 29], SVs discovered using LRS were genotyped with a relatively large amount of NGS data accumulated in previous studies. In this way, we could generate a comprehensive SV catalog and meanwhile reduce the average cost. Using the SVs discovered by the nanopore sequencing, we took Paragraph (v2.4a) [30] to genotype each genome generated using NGS data. We set the maximum allowed read count for SVs to 20 times the mean genome coverage for each dataset described above. BCFtools (v1.9) was used to merge all genotyped results. Coverage was assessed using mosdepth (v0.2.5) [31]. We also used CNVkit (v0.9.5) [32] to derive the depth-based absolute integer copy numbers for an average bin size of 100 bp around SVs with default thresholds.

In the meantime, we applied the HaplotypeCaller in GATK (v3.7) to call SNVs in each Chinese sample using NGS data. Only the variants that meet the following criteria were retained: (1) SNVs showed only two alleles; (2) the quality score > 20; and (3) SNVs have valid ancestral states derived from the human-chimp alignment published by the 1000 Genomes Project. Then, the genotypes from different panels were combined using BCFtools. SNVs were further phased and imputed by SHAPEIT (v4.1.2) without reference panels [33].

## Sample and variant quality control.

Firstly, we replaced all genotypes which failed to pass any filters by Paragraph with missing genotypes (./.). This method excludes variants having genotype conflicts or missing genotypes in one or more breakpoints. Then we counted the total number of non-reference SV per sample for each SV class (INS, DEL, INV, DUP, TRA). As suggested by a previous study [13], we removed three outlier samples whose SV count was either more than three times the inter-quartile range (IQR) beyond the third quartile or less than six times the IQR below the first quartile across all samples. Finally, we exclude SVs without any remaining non-reference genotypes after sample and variant quality control.

## Batch effects and Hardy–Weinberg equilibrium ﬁltering.

We assessed Hardy-Weinberg assumptions for each biallelic autosomal SV per each major population in our genotyping data, including Tibetans, Hans, and Africans. We calculated the p-value for each variant using the 'HardyWeinberg' package in R [34], then removed SVs which violate the Hardy-Weinberg assumption for any population after the Bonferroni correction (adjusted p-value < 0.05).

Then we assessed the concordance of SV calls between all pairs of the three short-read WGS collections used for SV genotyping. We firstly exclude SVs with a missing rate > 0.05 for any collections. As suggested by previous studies [13, 35], we counted the number of homozygous (1/1), heterozygous (1/0), reference (0/0) and missing sites (./.) for each SV, then performed a chi-squared test between all pairs of the three collections for Tibetans and Hans respectively. Because only two collections contain Tibetan samples, we could assess the potential batch effect by four pair comparisons. P-value was corrected by the Benjamini-Hochberg (BH) method. If SVs failed in only one comparison (p-value < 0.05), we marked the variant with a ‘VARIABLE_ACROSS_BATCHES’ tag in the VCF filter, and detailed results were recorded in Table S9. All SVs that failed in more than one comparison (p-value < 0.05) were defined as batch effect and were excluded from the following analysis.

## Population structure analysis

Using two NGS collections that contained both Tibetan and Han samples, we employed the Weir and Cockerham estimator for F_ST_ [36] based on VCFtools to identify the Tibetan-Han stratified SVs. We then performed permutation tests by shuffling population labels 1,000 times and calculated the empirical p-values. We determined a SV is population-stratified in Tibetans corresponding to Hans if (1) F_ST_ > 0.1,which screen out the top 1% of SVs, (2) empirical p-value < 0.05, and (3) missing rate < 0.05. To identify all candidate adaptive genes that tend to be regulated by these SVs, we considered three types of evidence-supported genes, including linear overlapping/nearest genes, 3D affected genes, and LD-linked eGenes.

### LD-linked eQTLs

To study the potential effects of SVs on gene expression, we extracted all significant variant-gene pairs of 49 tissues from the Genotype-Tissue Expression (GTEx) project (v8 release) [37]. Meanwhile, we used causal posterior probability (CPP) of fine-mapping results from CAVIAR to identify the causal variants[38, 39]. eQTLs with the most significant variants for genes or CPP > 0.1 were reserved for the following analysis. Then, we tested whether these casual variants and SVs were in LD ($\pm$1 Mb, r^2^ > 0.8). LD association tests were performed with VCFtools using both Tibetan and Han samples. Besides, we calculated the rank order for the effect size of these SV-associated eQTLs compared to other significant variants. Next, we identified the potentially regulated genes from LD-linked SV eQTLs and created a body map by Gene ORGANizer [40]. We also plotted the heatmaps of associated eQTLs across genes by LDheatmap [41].

### Chromatin conformational alterations

We applied a recently published algorithm to model the chromatin conformational changes caused by population-stratified SVs [42, 43]. Briefly, deletions can remove all overlapped anchors, and insertions will construct new CTCF anchors connected with the closest anchors if CTCF motifs were identified in the inserted sequences. We collected clusters of CTCF and RNA polymerase II derived from Chromatin Interaction Analysis by Paired-End Tag Sequencing (ChIA-PET) experiments and Hi-C maps of GM12878 from previous studies [43, 44]. Circular layouts were generated for the visualization of altered interactions by the ‘circlize’ package in R [45].

### Enrichment analysis

We predicted the effect of population-stratified SVs on transcripts and regulatory regions in the linear genome by the Ensembl Variant Effect Predictor [46]. Combined with the LD-linked genes and predicted affected genes in the 3D genome, we discovered a total of 80 protein-coding genes. Enriched biologically functional annotations of these candidate adaptive genes were analyzed by Metascape [47]. Besides, we collected the hypoxia-related pathways from a recent study [48] and expanded the list of known adaptive genes through literature analysis (Additional file 9: Table S14). Fisher’s exact test was performed to test the pathway enrichment and the p-value was corrected by the Bonferroni method.

**SV validation**

A polymerase chain reaction (PCR) assay was performed for validation of SVs. Firstly, a 500 bp genomic sequence surrounding each SV breakpoint was extracted from the GRCh37 assembly. Secondly, Primer3 (v.0.4.0) was used to compute a set of primer pairs flanking the breakpoint for these regions. Thirdly, PCR amplifications were performed in 25 μL reactions using the KOD-Plus-Neo high-fidelity thermostable DNA polymerase (Toyobo, Japan) on a BioRad c1000 Touch Thermal Cycler (Bio-Rad, USA). Finally, each PCR product of SV was genotyped by Sanger sequencing. The sequences of the primers are listed in Table S10.

## Demographic inference

As suggested in a recent study [49], we used SNVs to estimate the demographic history of Tibetans and Hans. SNVs were excluded if they overlapped with any low complexity regions in RepeatMasker annotations or self-chains (sequence identity > 0.9) in the UCSC Genome Browser Database. As suggested in previous reports [49-51], the coding sequences (Refseq genes from the UCSC Genome Browser Database) with 1,000 flanking base pairs were excluded to avoid potential effects of natural selection. All SNVs were pruned using PLINK (v1.9) in a window of 1,000 variants with a step size of 5 and a pairwise *r*^2^ threshold of 0.5. At last, an unfolded joint site frequency spectrum (SFS) of 876,639 SNVs from 160,656,471 non-genic autosomal bases was estimated using easySFS (https://github.com/isaacovercast/easySFS), polarized using ancestral alleles.

To explore the alternative demographic models for the population model TIB-HANN-HANS, we used the diffusion approximation method of ∂a∂i [52] to analyze the joint SFS. We tested three models with symmetric migrations to describe the simplified evolution paths. All three populations differentiated at the same time in the first model, while TIB branched out first in the other two models. Additional parameters were added to the third model to describe the possible changes in population size. To each model we tested, consecutive nine rounds of optimizations (100 replicates) were performed following the dadi_pipeline workflow [53]. For each round, we ran multiple replicates and used parameter estimates from the highest log-likelihood replicate to seed searches in the following round and optimized parameters using the derivative-based BFGS algorithm. To estimate the parameter uncertainties, we generated 100 nonparametric bootstrap replicates from the whole-genome data and estimated the confidence intervals for parameters using the Godambe information matrix [54]. All the scaled parameter estimates were transformed into real values using a mutation rate of 1.5 × 10^-8^ per site per generation [55] and a generation time of 29 years [49].

To discover the potential adaptive introgression from hominins into Tibetans, we grouped SNV genotypes of HANN and HANS into one cluster and then performed demographic inference for another population model YRI-TIB-HAN. We downloaded the SNV genotypes for YRI from the 1000 Genomes Project (phase 3). Using the same pipeline described above, we analyzed another unfolded joint SFS of 1,702,937 SNVs from 174,495,893 non-genic autosomal bases.

## Coalescent simulations

When the best-fit demographic model was recognized, we used msprime [56] to perform whole-genome coalescent simulations. To approximately account for mutational heterogeneity across the genome, we applied a three-step framework described in a previous study [50]. First, we divided the genome into windows of 50 kb and used ∂a∂i to estimate the population genetic mutation parameter θ given the best-fit demographic model. Second, we performed a whole-genome simulation with the θ_max_ estimated among all the windows. The HapMap recombination map [57] was also incorporated to model the recombination heterogeneity. Third, we changed the local mutation rate for each window by randomly dropping a proportion of $1-\frac{\theta_{j}}{\theta_{max}}$ of the simulated neutral variants. To account for the parameter uncertainties, we simulated 1,000 replicates of the best-fit demographic model by randomly sampling values from the confidence intervals of each parameter, assuming that they had a multivariate normal distribution. When simulating the best-fit demographic model for YRI-TIB-HAN, we included the Chimpanzee (n = 1) and archaic hominins (n = 3) branches as suggested in a recent study [49]. Genotypes for archaic genomes were collected via Neandertal and Denisovan Genome Projects. Confidence intervals for these relevant parameters were also drawn from this study.

At last, we estimated the per-base mutation rate using Watterson Estimator [58]. The significant correlation between the simulated and real data suggested that our whole-genome coalescent simulations could recapitulate the mutation patterns of SNVs. Furthermore, the genomic distribution of F_ST_ was remarkably different between the simulated and real data, indicating that the simulated data could identify non-neutral variants.

## Natural selection and archaic introgression

To detect evidence of natural selection in Tibetans, we calculated iHS [59] and XP-EHH [60] using an R package ‘rehh’ [61]. We detected selection signals for a sliding window of 100 SNVs and a step size of 50 SNVs, and then determined selection candidates if they were flanked by a significant window (p-value < 0.05, $\pm$500 kb) exclusively for Tibetans. The p-value of each window was defined as the proportion of simulations (TIB-HANN-HANS) with statistic values greater than those in the real data.

To detect evidence of archaic introgression, we applied the D-statistic and f_d_-statistic using an R package ‘admixr’ [62]. D-statistic and f_d_-statistic are designed to distinguish excess genetic drift from the ancient introgression based on allele frequencies of four populations. Following the instructions of a previous study [49], we defined the population relationships among three populations and an outgroup to be (((P1, P2), P3), O) = (((YRI, TIB), ARC), Chimpanzee), where ARC represents the Denisovan (DNS, n = 1) or Neanderthal (NDL, n = 2). Different from the relatively short window used for detecting natural selection, we applied the D-statistic and f_d_-statistic for a sliding window of 500 SNVs and a step size of 250 SNVs. At least 100 valid SNVs were required for each window. The simulation of the population model YRI-TIB-HAN was regarded as the null distribution in this step. Besides, we performed an S^*^-like method [63] as an orthogonal method to validate the signals of introgression. Similar to the original S^*^ statistic [64], this method utilizes linkage information and calculates a pairwise S^*^-like score *T* [63] between the target population (TIB) and the outgroup (YRI). We calculated the maximum score *T* for each sliding window defined by f_d_-statistic with a mutation rate of 1.5 × 10^-8^ per site per generation. The p-value of each window was defined as the proportion of simulations (YRI-TIB-HAN) with scores greater than those in the real data. We determined the candidate introgressed SVs if they were flanked by a significant window (p-value < 0.05 for D-statistic, f_d_-statistic, and S^*^-like statistic; $\pm$500 kb). We also required that the score *T* of an introgressed segment in the real data is larger than 15,000 as a global control of false positives across all population-stratified SVs (the 95% quantile of score *T* in simulation data is 7,289).

In the meantime, we performed hierarchical clustering for haplotypes by the R function ‘hclust’ with pairwise nucleotide differences as the input matrix. The TCS haplotype network was constructed using PopART [65].

## Functional explorations for the 335 bp deletion in 1p36.32

**Luciferase reporter gene assays**

The 1,335 bp genomic region surrounding the Chr1: 2,919,030-2,919,365 (GRCh37) was amplified and inserted into the upstream of SV40 promoter in the pGL3-promoter vector. Site-directed mutagenesis was employed to obtain either the deletion or the reference at this region. The SV40 promoter was subsequently replaced by the human *PRDM16* or *ACTRT2* promoter region (-2000 to +100 bp relative to the transcriptional start site). Primer sequences were listed in Table S21.

We seeded 0.5 × 10^6^ HUVEC or 1.5 × 10^6^ K562 cells per well in 12-well plates, and transfected them with luciferase reporter plasmids using X-tremegene HP DNA Transfection Reagent (Roche, Basel, Switzerland). The pRL-SV40 plasmid (Promega, USA) was co-transfected as a negative control. After 48 h of incubation, the cells were collected and analyzed for luciferase activity using the Dual-Luciferase Reporter Assay System (Promega, USA) on the GloMax platform (Promega, USA). All transfections were carried out in triplicate and were independently repeated at least three times.

**Generation of Knockout Cells by CRISPR/Cas9**

To delete the sequence of the 335 bp deletion in the HUVEC cell line, we designed two independent guide RNAs (gRNAs) using the CRISPR design tool (http://crispr.mit.edu). The gRNA sequences were as follows: #1: GGACCCTCTGTTAGTGAATC and #2: GTCCTCCAAACCTCACGCTG. The gRNA sequences (GTCCTCCAAACCTCACGCTG) were cloned into plasmid according to the standard protocol. The gRNA/Cas9 expression construct was co-transfected into HUVEC cells. After 24h transfection, the cells were transferred into 96-well plates at ~1 cell per well. After PCR screening, the candidate clones were analyzed by Sanger sequencing.

**qRT-PCR assays**

RNA samples were isolated using the RNeasy mini kit (Qiagen, Hilden, Germany) and were reversely transcribed into cDNAs with the high-capacity cDNA reverse transcription kit (Life Technologies, USA). Quantitative real-time PCR (qRT-PCR) assays were performed with iQ SYBR Green Supermix (#170-8862; Bio-Rad, USA) on an iQ5 real-time PCR detection system (Bio-Rad, USA). Each gene was assessed at least in triplicate using the ^∆∆^Ct method, and the β-actin (*ACTB*) was used as an internal control. The sequences of the primers were designed using Primer3 (v.0.4.0) and are listed in Table S22.

**Phenotype associations**

We used blood samples from 418 Tibetan highlanders and 72 Hans previously collected by our lab during a physical examination program at the community conducted in Shannan and measured 51 quantitative traits. A descriptive summary of the 51 quantitative traits in Tibetans is listed in Table S23. We genotyped the 335 bp deletion in these samples using PCR and Sanger sequencing. Besides, we performed association studies of 91 quantitative traits collected from 2,849 Tibetan samples [66].

# Supplementary Figures

**
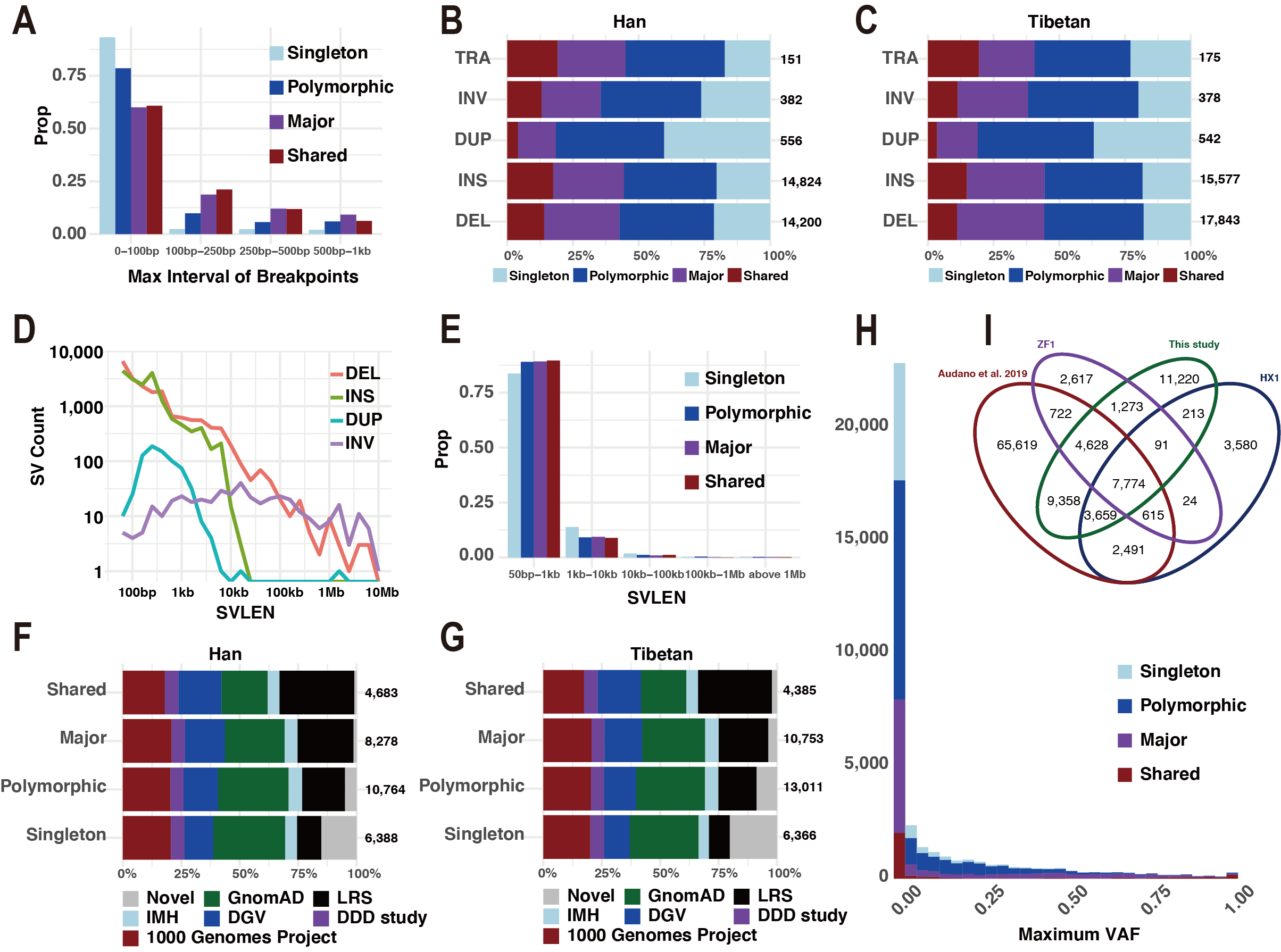
**

**Fig. S1 Discovery of structural variations in 25 samples using the nanopore sequencing technology. (**A) Maximum interval of breakpoints determined in different samples for each discovery category, including the shared (identified in all samples), major (identified in ≥ 50% of samples), polymorphic (identified in > 1 sample), and singleton (identified in only one sample) structural variations (SVs). (B) and (C) are the frequencies for each SV type in Hans and Tibetans: translocation (TRA), inversion (INV), duplication (DUP), insertion (INS), and deletion (DEL). (D) and (E) Distribution of SV length (SVLEN) for each discovery category. (F) and (G) Proportions for SVs for Hans and Tibetans discovered in previously published SV calls for each discovery category. LRS represents SV calls identified from long-sequence data including HX1, ZF1 and a multi-population study published by Audano et al. IMH represents a common disease trait mapping study published by Ira M. Hall’s lab. DDD study represents the Deciphering Developmental Disorders Study. (H) The maximum variant allele frequencies (VAF) reported in previously published SV calls for each discovery category. (I) Comparisons between SV calls from different long-read sequencing studies.

**
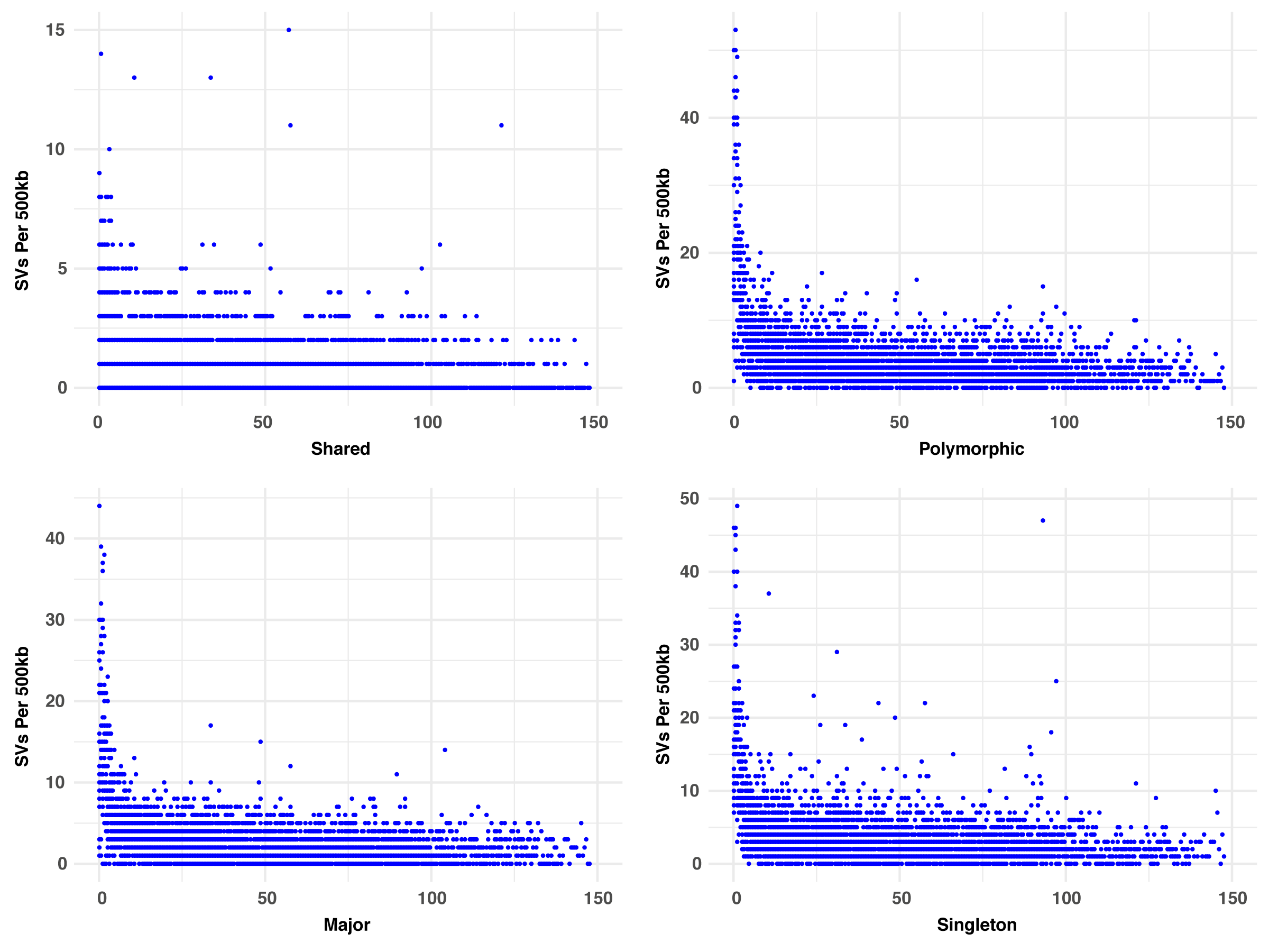
**

**Fig. S2 Distribution of structural variations.** Each chromosome was divided into continuous 500 kilobase (kb) windows and the number of structural variations (SVs) overlapped with the window was summed for each discovery category, including the shared (identified in all samples), major (identified in ≥ 50% of samples), polymorphic (identified in > 1 sample), and singleton (identified in only one sample) SVs.

**
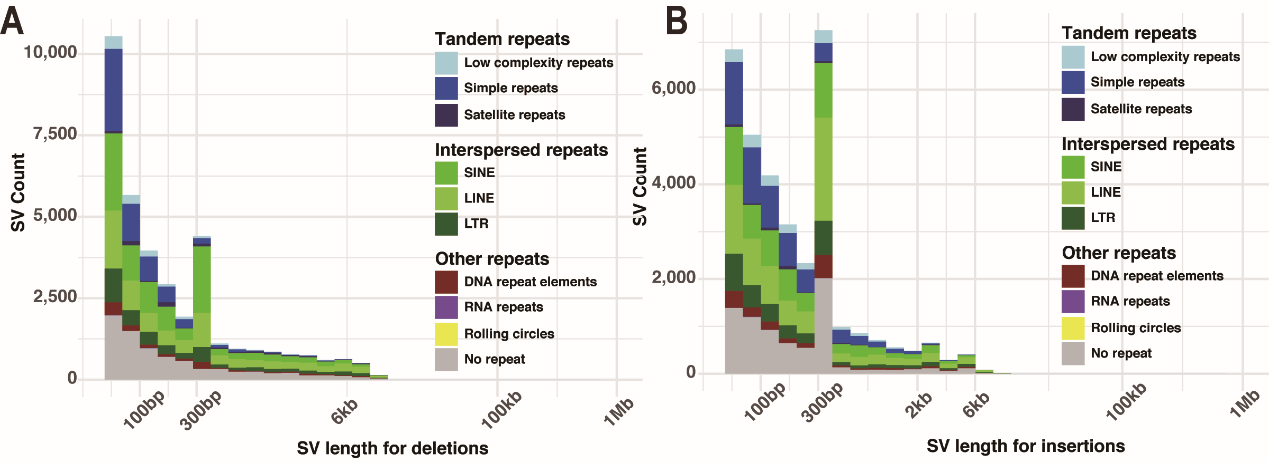
**

**Fig. S3 Distribution of deletions and insertions classified by intersected repeat elements.** Repetitive elements are summarized into nine different classes, including the low complexity repeats, simple repeats, satellite repeats, short (SINE) and long interspersed nuclear elements (LINE), long terminal repeat elements (LTR), DNA repeat elements, RNA repeats, and Rolling circles.

**
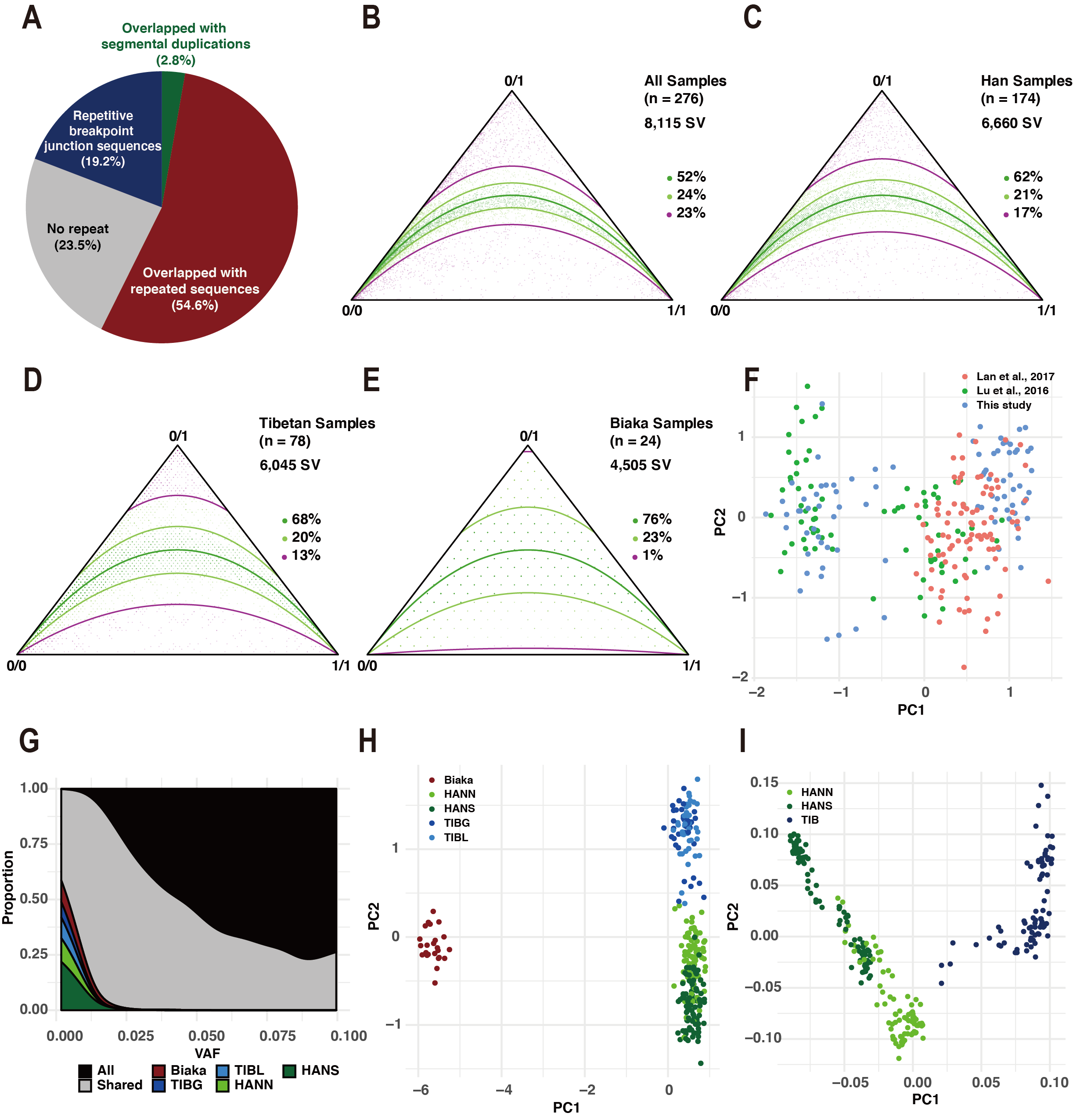
**

**Fig. S4 Genotyping results and population structure of short-read sequencing genomes.** (A) Proportions for repeat elements intersected with structural variations (SVs) which were not supported by any next-generation sequencing (NGS) genomes. (B), (C), (D), and (E) are Hardy–Weinberg equilibrium ﬁlterings for Han, Tibetan, and Biaka samples. (F) Principle component analysis (PCA) of SV genotypes for Tibetans and Hans. Different Colours represent NGS collections used for genotyping. (G) Profiles of variant allele frequencies (VAF) for each population, including Hans in North China (HANN) or South China (HANS), Tibetans living above (TIBG) or below (TIBL) 4,000 meters, and Biaka populations. (H) PCA of SV genotypes for Biaka, Tibetans, and Hans. (I) PCA of whole-genome SNV genotypes for Tibetans and Hans.

**
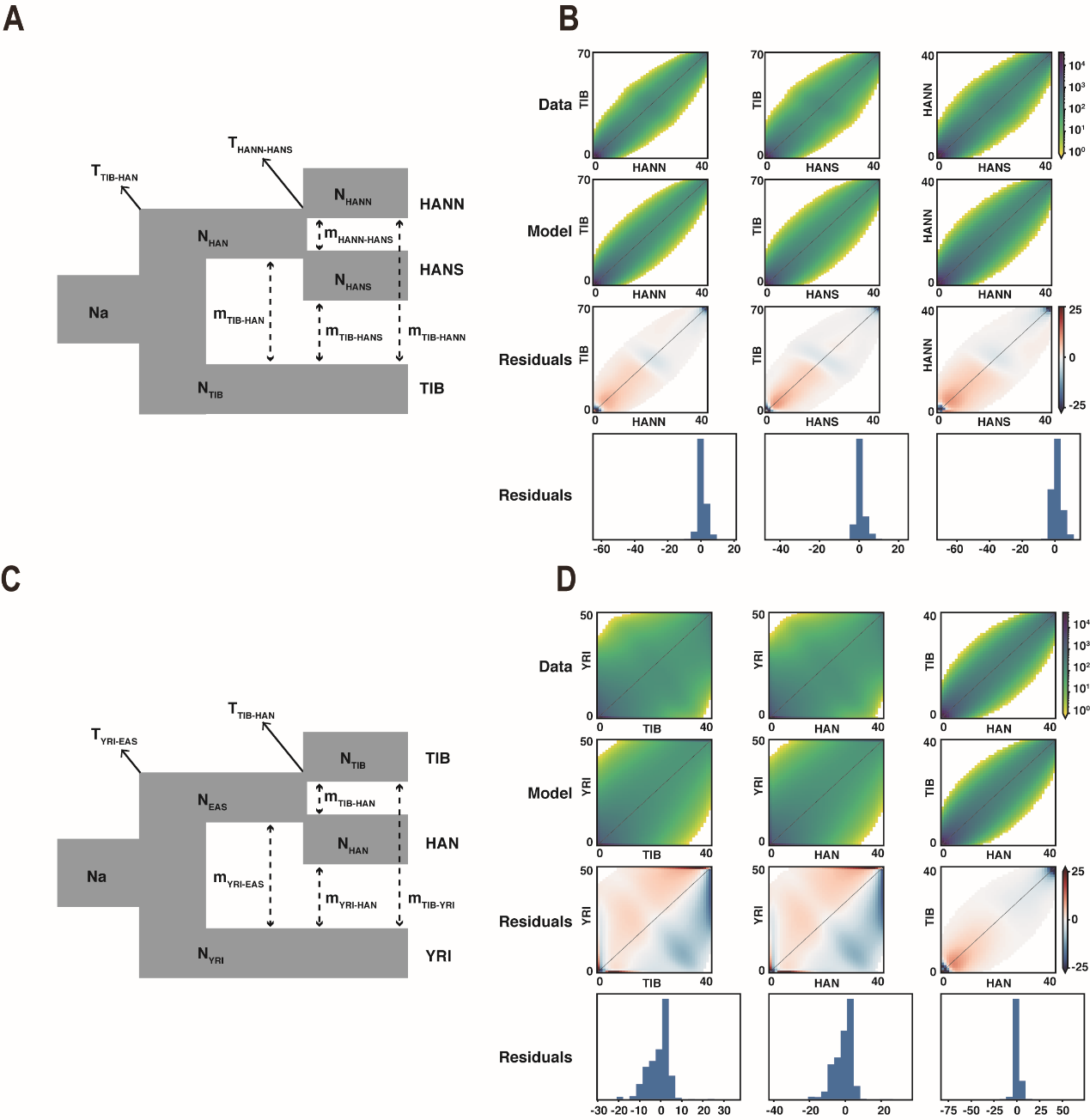
**

**Fig. S5 Demographic inferences for the best-fit demographic models. (**A) The best-fit demographic model for Tibetans (TIB), Hans in North China (HANN), and South China (HANS). (C) The best-fit demographic model for Tibetans (TIB), Hans (HAN), and Yoruba samples in Ibadan (YRI). The corresponding maximum likelihood estimates and descriptions of parameters can be found in Table S7. (B) and (D) The observed and predicted frequency spectra for the two population models, respectively. The first row is the real data, and the second row is the best-fit model. The third and the fourth row are the residuals of the model minus data.

**
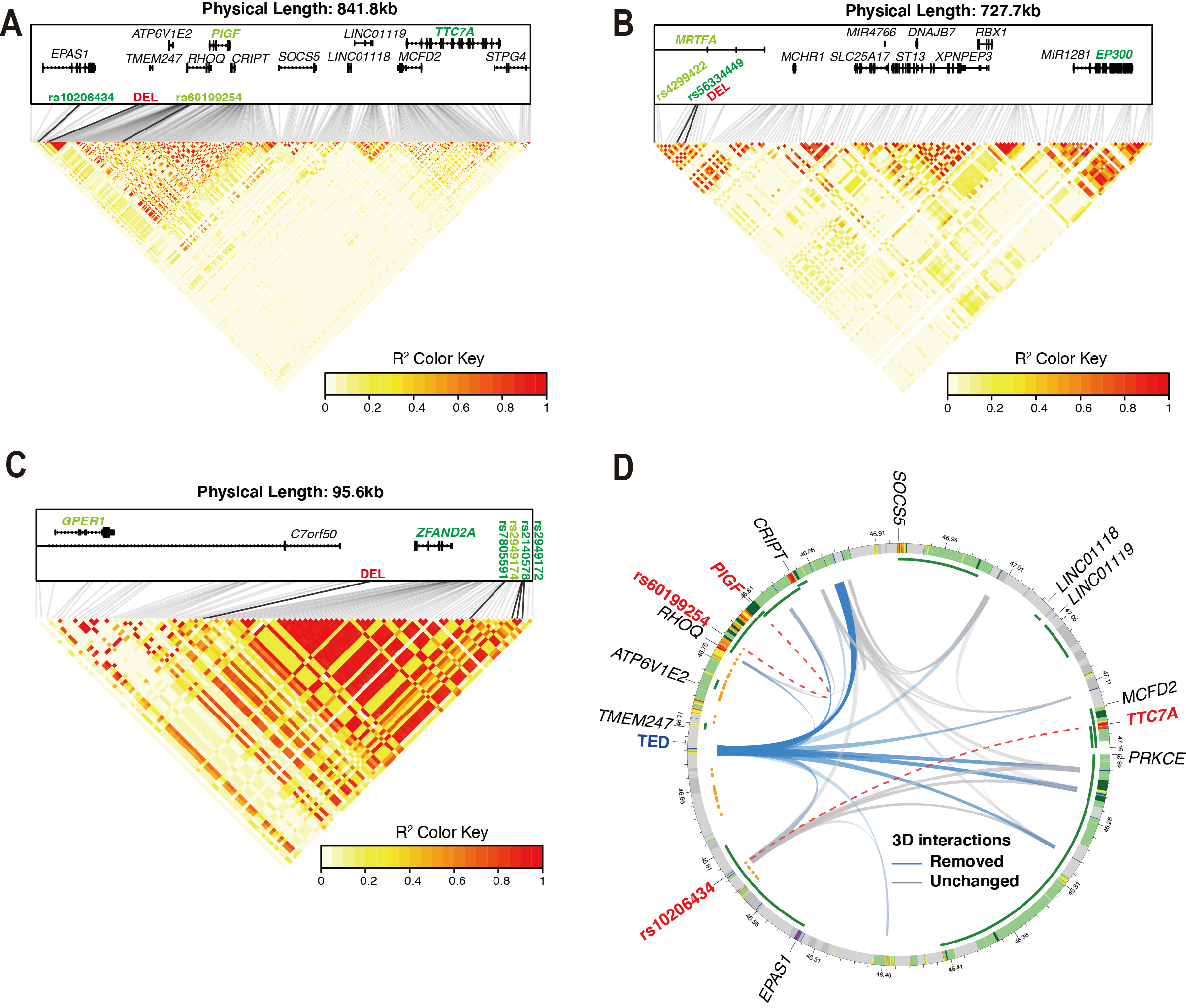
**

**Fig. S6 Heatmaps for the expression quantitative trait locus (eQTLs) surrounding three deletions, including** (A) the 3.4 kilobase (kb) Tibetan enriched deletion (TED), (B) the 163 bp deletion near *MRTFA*, and (C) the 2,590 bp deletion between *ZFAND*2A and GPER1. The linkage disequilibrium (LD)-linked eQTLs and their associated genes are marked as green. (D) Profile of all CTCF interactions near the 3.4 kilobase (kb) Tibetan-enriched deletion (TED). The outermost layer shows the 15-core chromatin states from RoadMap. The inner green layer represents the transcription regions of affected genes. Among single nucleotide variants (SNVs) highly linked with TED (orange points), rs60199254 and rs10206434 act as expression quantitative trait locus (eQTL) exhibiting relationships with expression levels of PIGF and TTC7A, respectively (red dashed lines).

**
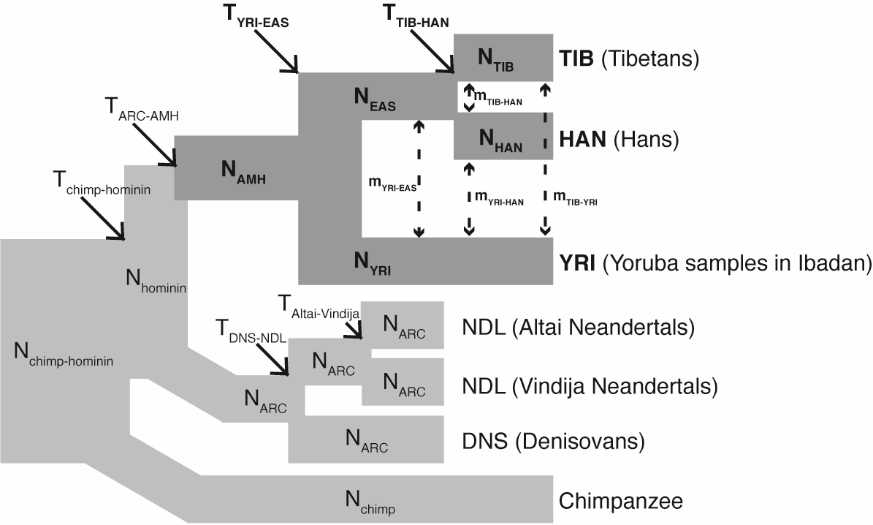
**

**Fig. S7 Demographic model for the whole-genome coalescent simulations for Tibetans, Hans, and Yoruba samples in Ibadan.** Dark branches and bold parameters indicate the best-fit demographic model inferred in this study (Table S7). Parameter estimates of the light branches were uniformly drawn from the 95% confidence interval (CI) reported in a previous study (Table S8).

**
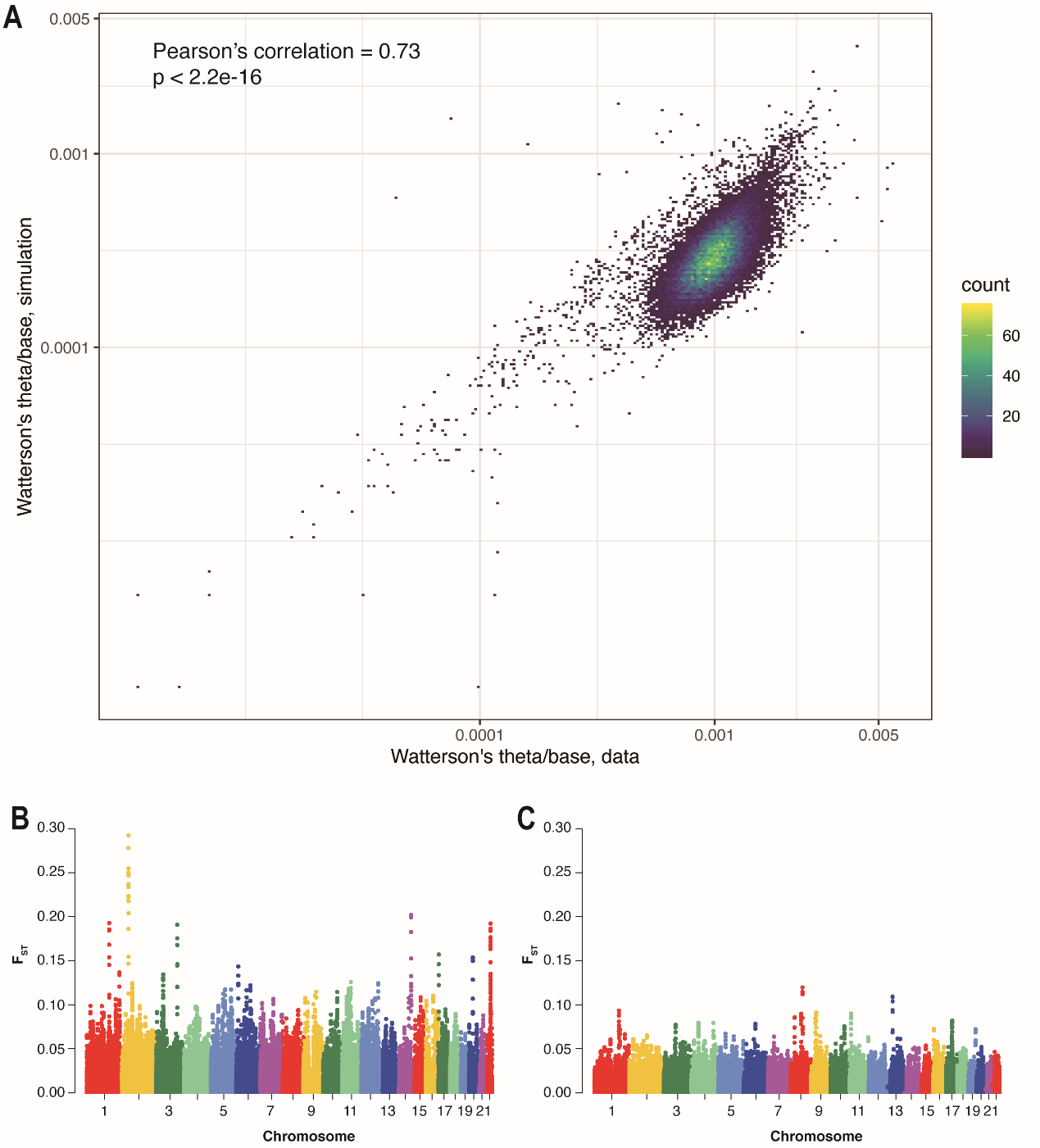
**

**Fig. S8 Whole-genome simulations for Tibetans, Hans in North China and South China.** (A) Correlation of per-base Watterson Estimator between the simulated and real data. (B) and (C) The Manhattan plots for the window-based F_ST_ statistics (Tibetans *vs.* Hans) using the real and simulated data based on one of 1,000 replicates.

**
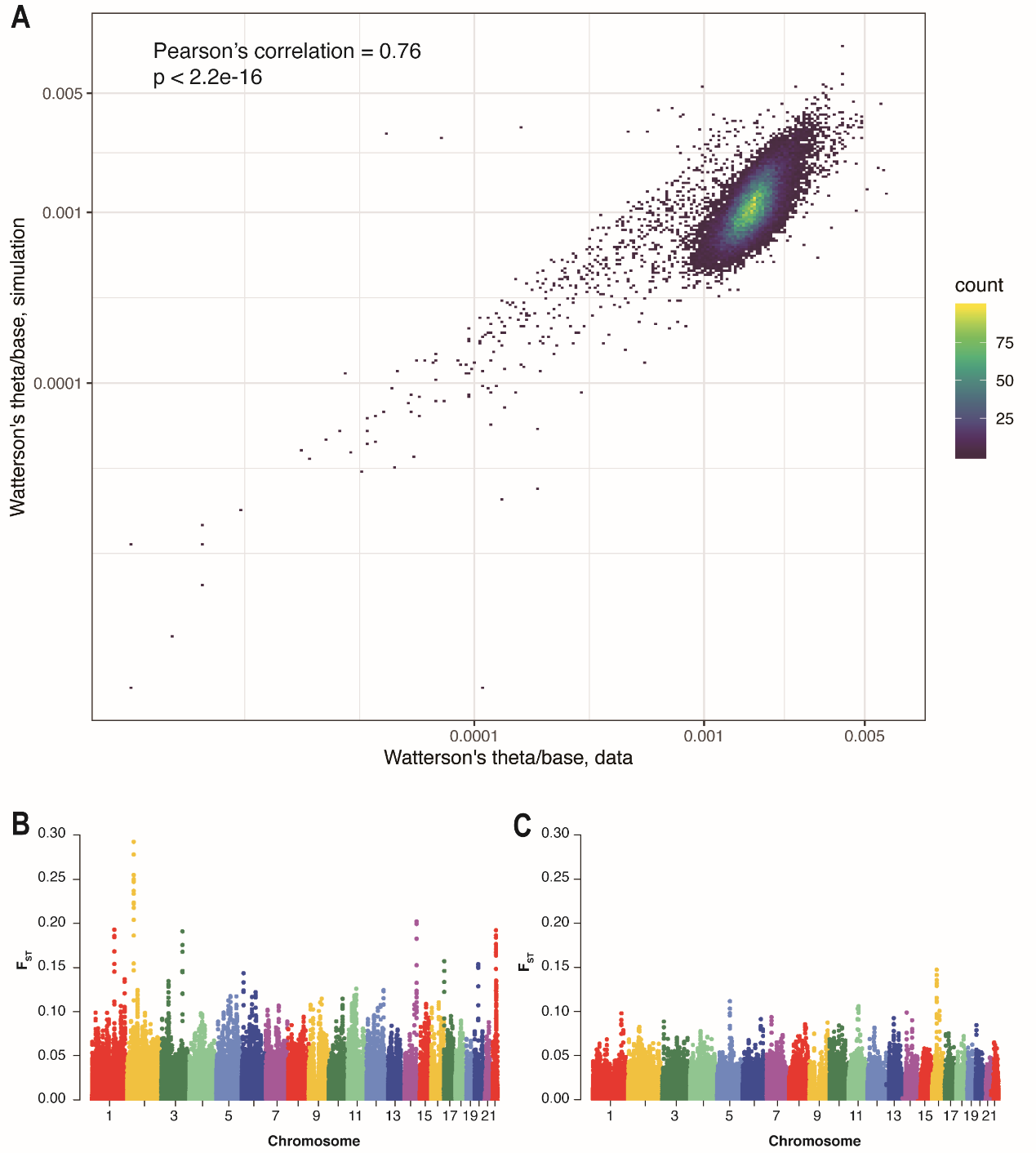
**

**Fig. S9 Whole-genome simulations for Tibetans, Hans, and Yoruba samples in Ibadan.** (A) Correlation of per-base Watterson Estimator between the simulated and real data. (B) and (C) The Manhattan plots for the window-based F_ST_ statistics (Tibetans *vs.* Hans) using the real and simulated data based on one of 1,000 replicates.

**
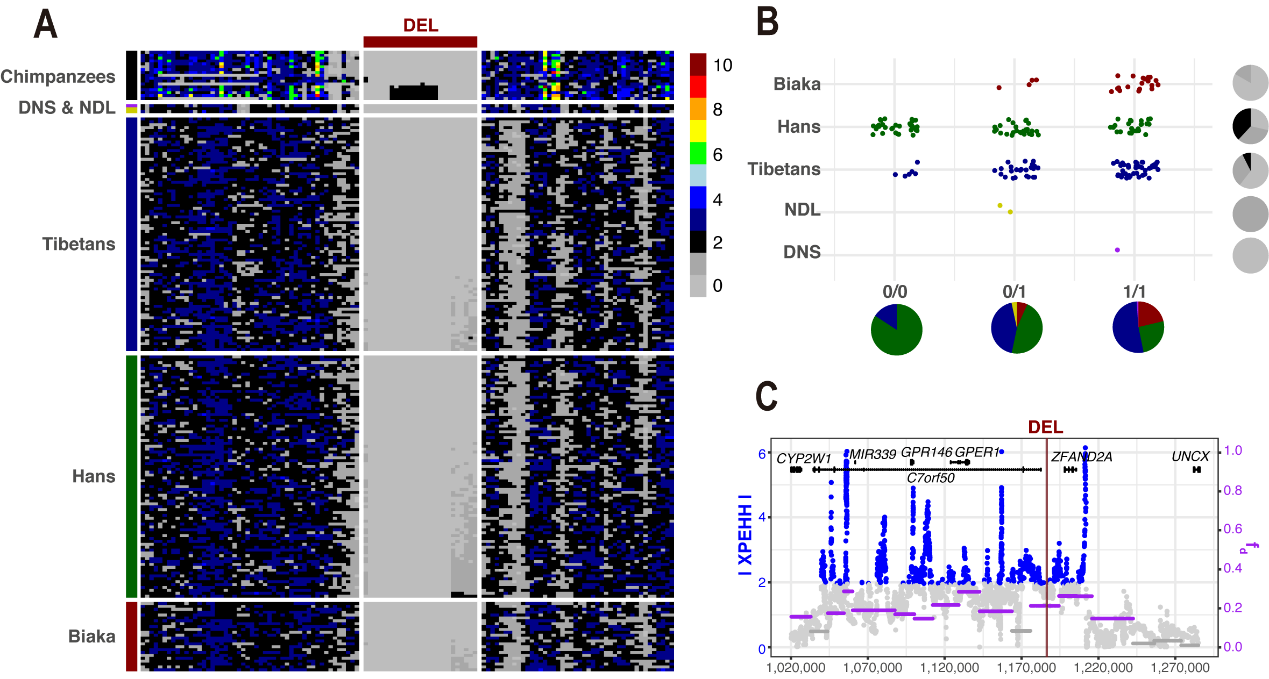
**

**Fig. S10 Signatures of selection and archaic introgression from Neandertals (NDL) and Denisovans (DNS) for the deletion at 7p22.3 (chr7:1,185,070-1,187,063).** (A) Absolute integer copy numbers for a sliding window of 100 base pairs (bp) and a step size of 50 bp around the deletion. Each row represents the copy numbers of a sample over the region. (B) Genotyping results. Pie charts along the x-axis indicate the population distribution for different structural variation (SV) genotypes (colors are the same as populations), and pie charts along the y-axis illustrate the frequency distribution for a given population (colors are the same as copy number of 2/1/0). (C) Distributions of single nucleotide polymorphisms (SNPs) with significant f_d_-statistic (purple dots) and cross-population Extended Haplotype Homozygosity (XP-EHH, blue dots).

**
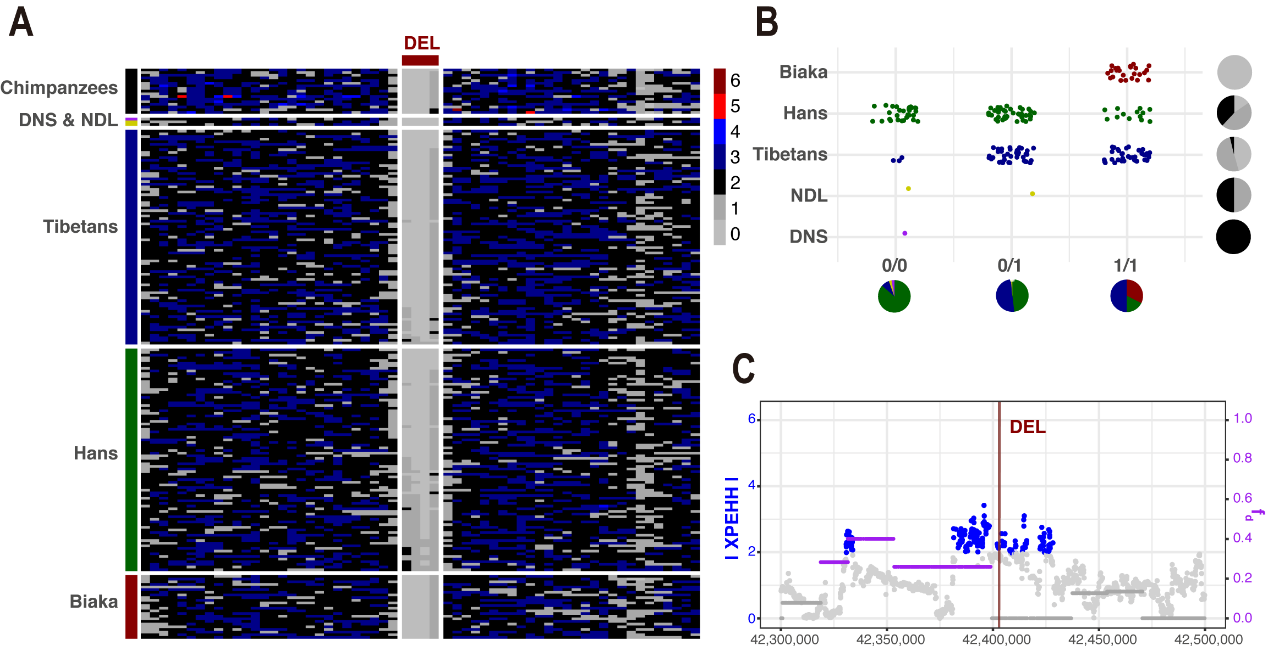
**

**Fig. S11 Signatures of selection and archaic introgression from Neandertals (NDL) and Denisovans (DNS) for the deletion at 21q22.2 (chr21:42,402,890-42,403,212).** (A) Absolute integer copy numbers for a sliding window of 100 base pairs (bp) and a step size of 50 bp around the deletion. Each row represents the copy numbers of a sample over the region. (B) Genotyping results. Pie charts along the x-axis indicate the population distribution for different structural variation (SV) genotypes (colors are the same as populations), and pie charts along the y-axis illustrate the frequency distribution for a given population (colors are the same as copy number of 2/1/0). (C) Distributions of single nucleotide polymorphisms (SNPs) with significant f_d_-statistic (purple dots) and cross-population Extended Haplotype Homozygosity (XP-EHH, blue dots).


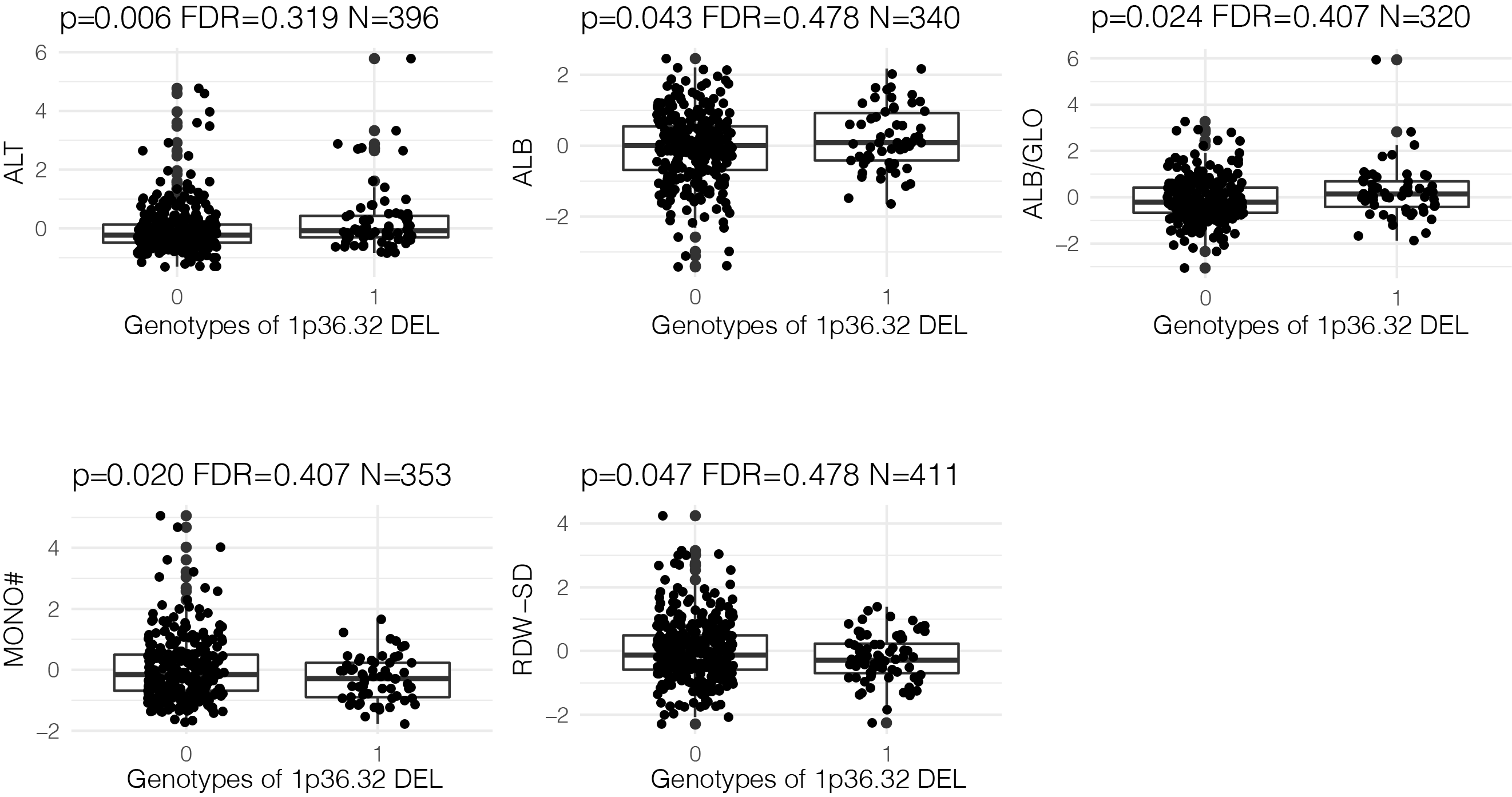


**Fig. S12 Phenotype associations for the deletion at 1p36.32 with 51 quantitative tracts from 418 Tibetans (LD > 0.8).** Significant associations were found with ALT (Alanine Aminotransferase), ALB (Albumin), ALB/GLO (Albumin/Globulin), MONO# (Mononuclear), and RDW-SD (standard variations of Platelet distribution width).


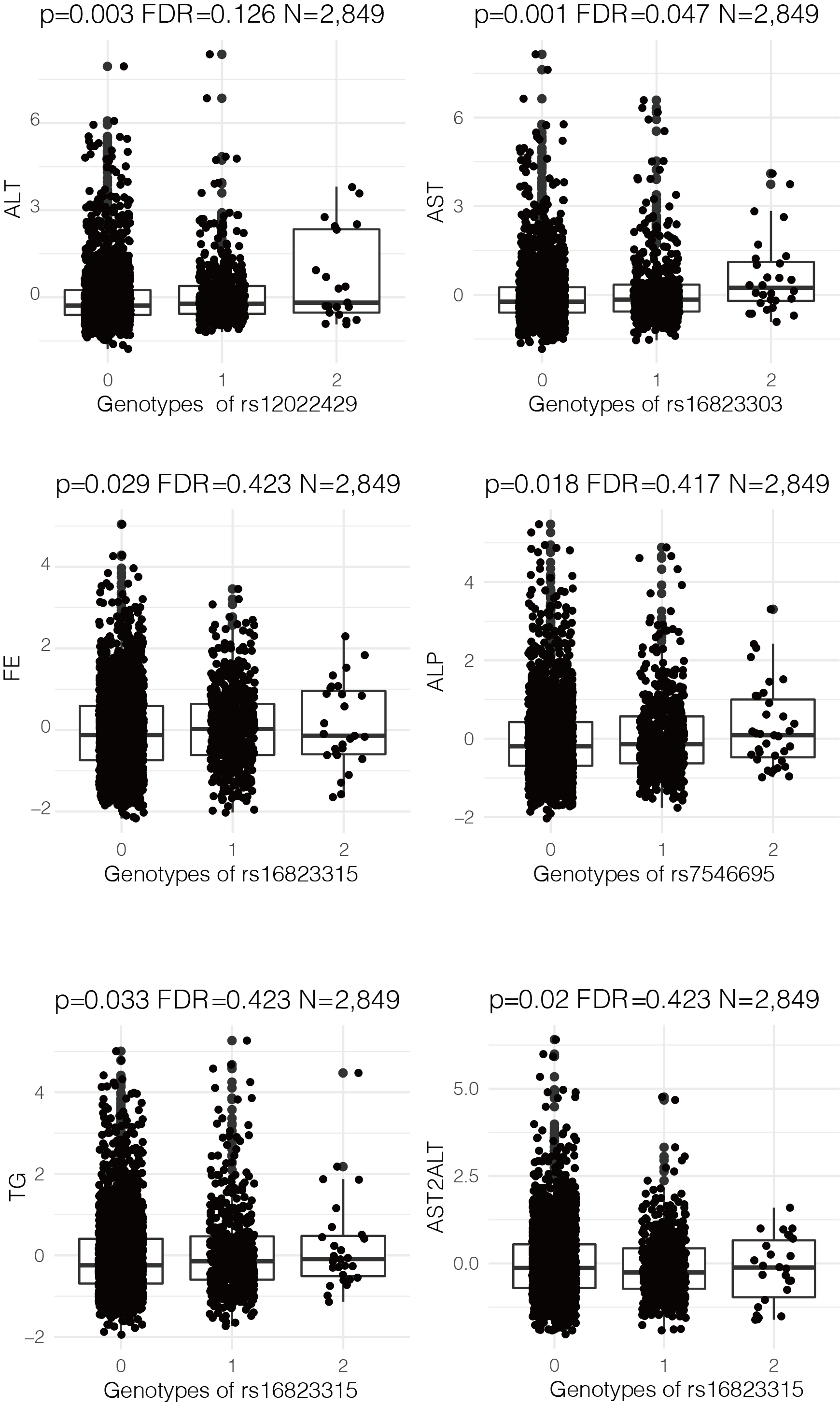


**Fig. S13 Phenotype associations for the SNPs in LD with the deletion at 1p36.32 with 91 quantitative tracts from 2,849 Tibetans.** Significant associations were found with ALT (Alanine Aminotransferase), AST (Aspartate aminotransferase), FE (Ferrum), ALP (Alkaline phosphatase), TG (Triglyceride), and AST2ALT (AST/ALT ratio).


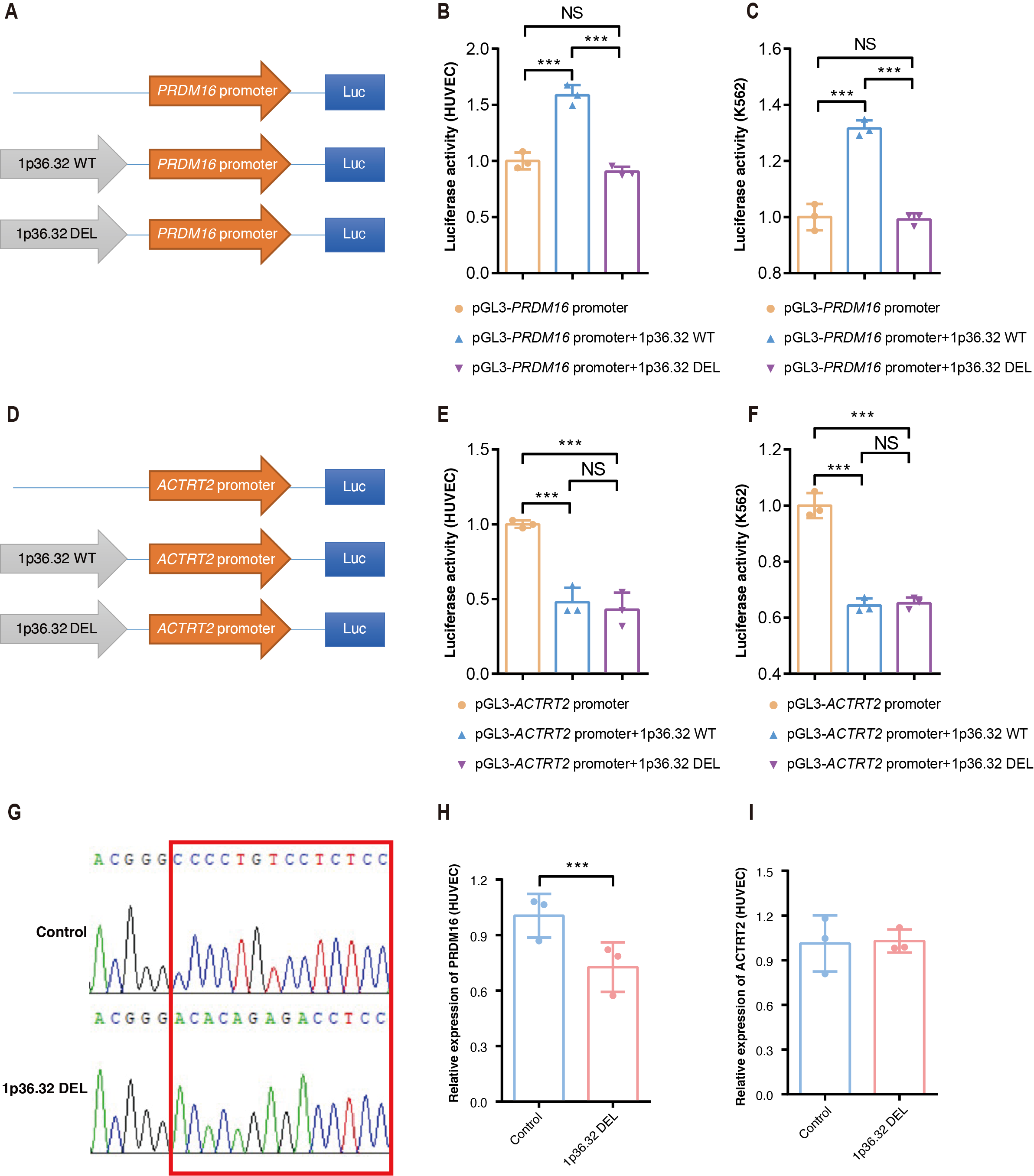


**Fig. S14 Luciferase reporter gene and knockout assays for the deletion at 1p36.32 (chr1:2,919,030-2,919,365).** (A) and (D) shows three different conditions for luciferase gene assays for *PRDM16* and *ACTRT2* respectively. (B) and (C) are the results of luciferase reporter gene assays for *PRDM16* in HUVEC and K562 cells. (E) and (F) are the results of Luciferase reporter gene assays for *ACTRT2* in HUVEC and K562 cells. (G) Sanger sequencing of CRISPR/Cas9-modified and control cells. (H) and (I) Quantitative real-time PCR analysis of the mRNA levels of *PRDM16* and *ACTRT2* in CRISPR/Cas9-modified and control HUVEC cells.

# Supplementary Tables

**Table S1. Sample information for SV discovery.**

| **SampleID** | **Platform** | **Populations** | **Locations** | **Altitude (meters)** | **Depth of coverage** | **Discovery (Supp=1)** | **Discovery (Supp=2)** | **Discovery (Supp=3)** |
| --- | --- | --- | --- | --- | --- | --- | --- | --- |
| CQ082 | ONT | Tibetan | Hainanzhou | 3,000 | 21.83 | 21,659 | 18,218 | 13,837 |
| CQ085 | ONT | Tibetan | Shigatse | 4,000 | 16.76 | 21,286 | 16,807 | 12,452 |
| CQ091 | ONT | Tibetan | Chamdo | 3,200 | 26.97 | 21,612 | 18,546 | 14,706 |
| CQ095 | ONT | Tibetan | Shigatse | 4,000 | 18.13 | 21,065 | 17,478 | 13,367 |
| CQ104 | ONT | Tibetan | Shigatse | 4,000 | 19.22 | 21,047 | 17,677 | 13,717 |
| CQ115 | ONT | Tibetan | Shigatse | 4,000 | 19.65 | 20,974 | 17,732 | 13,785 |
| CQ121 | ONT | Tibetan | Nyingchi | 3,000 | 13.91 | 19,723 | 15,445 | 11,493 |
| CQ123 | ONT | Tibetan | Hainanzhou | 3,000 | 19.35 | 21,562 | 17,664 | 13,274 |
| CQ132 | ONT | Tibetan | Chamdo | 3,200 | 19.47 | 20,931 | 17,508 | 13,362 |
| CQ136 | ONT | Tibetan | Shigatse | 4,000 | 16.39 | 20,537 | 16,833 | 12,789 |
| CQ143 | ONT | Tibetan | Hainanzhou | 3,000 | 22.97 | 21,604 | 18,284 | 14,201 |
| CQ144 | ONT | Tibetan | Hainanzhou | 3,000 | 20.29 | 21,134 | 17,915 | 13,984 |
| CQ152 | ONT | Tibetan | Hainanzhou | 3,000 | 24.75 | 21,601 | 18,465 | 14,541 |
| CQ188 | ONT | Tibetan | Shigatse | 4,000 | 21.55 | 21,151 | 17,938 | 14,163 |
| CQ200 | ONT | Tibetan | Shigatse | 4,000 | 14.45 | 20,310 | 15,631 | 11,473 |
| CQ232 | ONT | Han | Bijie | 1,500 | 29.30 | 20,945 | 18,602 | 15,372 |
| CQ233 | ONT | Han | Kunming | 1,500 | 11.61 | 18,495 | 14,017 | 10,578 |
| CQ234 | ONT | Han | Zigong | 300 | 27.13 | 20,828 | 18,535 | 15,148 |
| CQ235 | ONT | Han | Lishui | 300 | 24.64 | 20,710 | 18,388 | 15,057 |
| CQ236 | ONT | Han | Ganzhou | 100 | 21.93 | 20,200 | 17,866 | 14,426 |
| CQ292 | ONT | Han | Fuzhou (Jiangxi) | 100 | 15.43 | 19,597 | 16,295 | 12,147 |
| CQ294 | ONT | Han | Panzhihua | 1,000 | 16.03 | 19,897 | 16,787 | 12,923 |
| CQ295 | ONT | Han | Wuyuan | 100 | 13.24 | 19,227 | 15,596 | 11,939 |
| CQ296 | ONT | Han | Ya'an | 500 | 18.51 | 20,320 | 17,333 | 13,475 |
| CQ297 | ONT | Han | Zhengzhou | 100 | 25.96 | 20,628 | 18,402 | 15,110 |

Abbreviations: ONT, Oxford nanopore technologies; SV, structural variation;

The last three columns are the discovery of SVs with different supporting number of SV callers, including NanoSV, SVIM, and Sniffles

**Table S3. Enrichment analysis results.**

| **Characteristics** | **Description** | **LFC** | **p-value** | **sd** |
| --- | --- | --- | --- | --- |
| SD | Segmental duplications | 0.168 | <0.001 | 0.053 |
| CpG | CpG islands | 1.861 | <0.001 | 0.156 |
| TAD | Topologically associating domains | -0.041 | <0.001 | 0.017 |
| CTCF | CTCF binding clusters | -0.551 | <0.001 | 0.090 |
| RepClass: Low complexity repeats | Tandem repeats | 1.176 | <0.001 | 0.055 |
| RepClass: Simple repeats | Tandem repeats | 3.410 | <0.001 | 0.119 |
| RepClass: Satellite repeats | Tandem repeats | 2.306 | <0.001 | 0.128 |
| RepClass: SINE | Short interspersed nuclear element | 0.265 | <0.001 | 0.016 |
| RepClass: LINE | Long interspersed nuclear element | -0.356 | <0.001 | 0.014 |
| RepClass: LTR | Long terminal repeats | -0.314 | <0.001 | 0.017 |
| RepClass: DNA | DNA repeat elements | -0.572 | <0.001 | 0.029 |
| RepClass: RNA | RNA repeat elements | -0.139 | 1 | 0.167 |
| RepClass: RC | Rolling circles | -1.460 | <0.001 | 0.348 |
| RepClass: No repeats |  | -0.583 | <0.001 | 0.020 |
| SVTK: UTR | Untranslated regions | -1.766 | <0.001 | 0.117 |
| SVTK: DUP_PARTIAL | Patial-gene duplications | -0.018 | 1 | 0.138 |
| SVTK: LOF | Loss-of-function variants | -1.887 | <0.001 | 0.092 |
| SVTK: INV_SPAN | Whole-gene inversions | 0.546 | 0.630 | 0.509 |
| SVTK: INTRONIC | Intronic SVs | -0.011 | 1 | 0.013 |
| SVTK: INTERGENIC | Intergenic SVs | 0.079 | <0.001 | 0.010 |
| ExAC: synZ | Intolerance to synonymous variations | -0.015 | 0.435 | 0.012 |
| ExAC: misZ | Intolerance to missense variations | -0.016 | 0.295 | 0.010 |
| ExAC: pLI | Intolerance to loss of function variations | -0.173 | <0.001 | 0.021 |
| Dosage-sensitive: HI_CGscore | haploinsufficiency | -0.113 | 0.075 | 0.059 |
| Dosage-sensitive: HI_DDDpercent | dosage pathogenicity | -0.432 | <0.001 | 0.025 |
| ChromHMM: 1_TssA | Active TSS | -0.278 | <0.001 | 0.106 |
| ChromHMM: 2_TssAFlnk | Flanking Active TSS | -1.356 | <0.001 | 0.088 |
| ChromHMM: 3_TxFlnk | Transcr. at gene 5' and 3' | -1.287 | <0.001 | 0.207 |
| ChromHMM: 4_Tx | Strong transcription | -0.552 | <0.001 | 0.046 |
| ChromHMM: 5_TxWk | Weak transcription | 0.145 | <0.001 | 0.017 |
| ChromHMM: 6_EnhG | Genic enhancers | -0.927 | <0.001 | 0.071 |
| ChromHMM: 7_Enh | Enhancers | -0.890 | <0.001 | 0.041 |
| ChromHMM: 8_ZNF | ZNF genes & repeats | 0.882 | <0.001 | 0.134 |
| ChromHMM: 9_Het | Heterochromatin | 0.564 | <0.001 | 0.043 |
| ChromHMM: 10_TssBiv | Bivalent/Poised TSS | 0.031 | 1 | 0.144 |
| ChromHMM: 11_BivFlnk | Flanking Bivalent TSS/Enh | -0.279 | 1 | 0.164 |
| ChromHMM: 12_EnhBiv | Bivalent Enhancer | -0.500 | <0.001 | 0.110 |
| ChromHMM: 13_ReprPC | Repressed PolyComb | 0.018 | 1 | 0.067 |
| ChromHMM: 14_ReprPCWk | Weak Repressed PolyComb | 0.403 | <0.001 | 0.023 |
| ChromHMM: 15_Quies | Quiescent/Low | -0.054 | <0.001 | 0.005 |

**Table S5. Comparison of three demographic models for TIB-HANN-HANS.**

| **Model ID** | **Model** | **Description** | **Parameter: estimate** | | | **LL** | **AIC** |
| --- | --- | --- | --- | --- | --- | --- | --- |
|  |  |  | **Effective population size** | **Divergence time (years)** | **Migration rate** |  |  |
| Model 1 (7 parameters) | 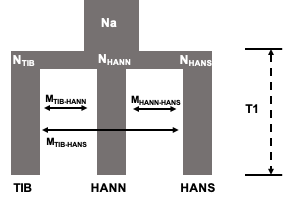 | All three populations differentiated at the same time, including Tibetans (TIB), Hans in the North (HANN), and Hans in the South (HANS). | Na: 14,582 N_TIB_: 33,815 N_HANN_: 72,561 N_HANS_: 31,949 | T1: 14,382 | M_TIB-HANN_: 0.0606 M_HANN-HANS_: 0.0013 M_TIB-HANS_: 0.0089 | -162,142 | 324,298 |
| Model 2 (10 parameters) | 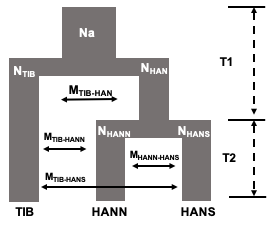 | TIB branched out first | Na: 10,206 N_TIB_: 36,260 N_HAN_: 50,870 N_HANN_: 50,667 N_HANS_: 1,632 | T1: 16,871 T2: 769 | M_TIB-HAN_: 0.0016  M_TIB-HANN_: 0.0087 M_HANN-HANS_: 0.0537 M_TIB-HANS_: 0.003 | -152,792 | 305,605 |
| Model 3 (17 parameters) | 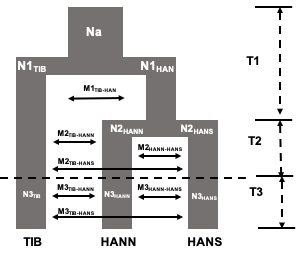 | TIB branched out first and population size changed | Na: 10,853 N1_TIB_: 43,328 N1_HAN_: 14,389 N2_HANN_: 50,667 N2_HANS_: 6,318 N3_TIB_: 21,323 N3_HANN_: 54,195 N3_HANS_: 32,581 | T1: 3,147 T2: 1,133 T3: 9,442 | M1_TIB-HAN_: 0.0018  M2_TIB-HANN_: 0.0085 M2_HANN-HANS_: 0.081 M2_TIB-HANS_: 0.0058 M3_TIB-HANN_: 0.0988 M3_HANN-HANS_: 0.001 M3_TIB-HANS_: 0.0722 | -160,563 | 321,161 |

LL, log-likelihood; AIC, Akaike information criterion.

**Table S6. Comparison of three demographic models for YRI-TIB-HAN.**

| **Model ID** | **Model** | **Description** | **Parameter: estimate** | | | **LL** | **AIC** |
| --- | --- | --- | --- | --- | --- | --- | --- |
|  |  |  | **Effective population size** | **Divergence time (years)** | **Migration rate** |  |  |
| Model 1 (7 parameters) | 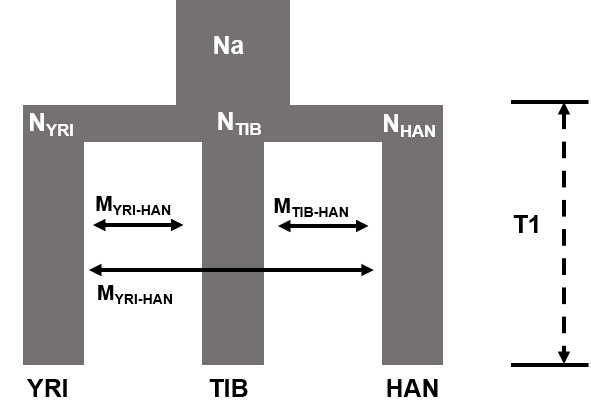 | All three populations differentiated at the same time, including Yoruba samples in Ibadan (YRI), Tibetans (TIB), Hans (HAN). | Na: 12,452 N_YRI_: 11,187 N_TIB_: 29,549 N_HAN_: 31,190 | T1: 63,700 | M_YRI-TIB_: 0.0879 M_TIB-HAN_: 0.0018 M_YRI-HAN_: 0.0882 | -453,165 | 906,345 |
| Model 2 (10 parameters) | 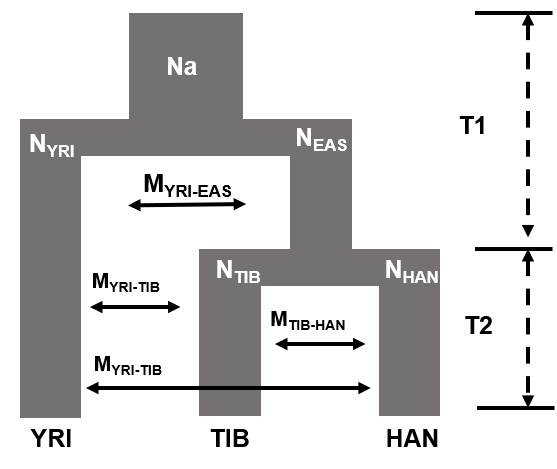 | YRI branched out first | Na: 12,495 N_YRI_: 28,885 N_EAS_: 8,906 N_TIB_: 34,657 N_HAN_: 43,446 | T1: 65,662 T2: 22,974 | M_YRI-EAS_: 0.0166  M_YRI-TIB_: 0.0983 M_TIB-HAN_: 0.0072 M_YRI-HAN_: 0.001 | -223,444 | 446,909 |
| Model 3 (17 parameters) | 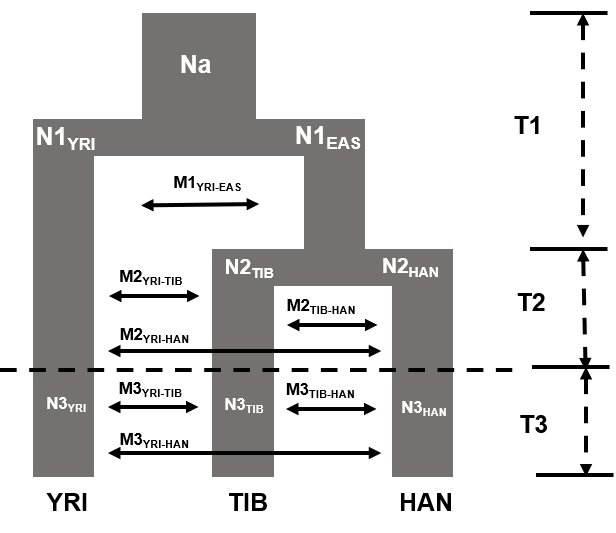 | YRI branched out first and population size changed | Na: 12,924 N1_YRI_: 35,442 N1_EAS_: 8,264 N2_TIB_: 48,819 N2_HAN_: 38,663 N3_YRI_: 2,807 N3_TIB_: 62,909 N3_HAN_: 64,086 | T1: 62,144 T2: 22,414 T3: 1,274 | M1_YRI-EAS_: 0.0063  M2_YRI-TIB_: 0.0984 M2_TIB-HAN_: 0.0013 M2_YRI-HAN_: 0.001 M3_YRI-TIB_: 0.0113 M3_TIB-HAN_: 0.0035 M3_YRI-HAN_: 0.0107 | -224,443 | 448,921 |

LL, log-likelihood; AIC, Akaike information criterion.

**Table S7. Maximum likelihood parameter estimates and confidence intervals of best-fit demographic models.**

| **Best-fit model for TIB-HANN-HANS** | | | | **Best-fit model for YRI-TIB-HAN** | | | |
| --- | --- | --- | --- | --- | --- | --- | --- |
| **Parameters** | **Descriptions** | **Estimates** | **95% CIs** | **Parameters** | **Descriptions** | **Estimates** | **95% CIs** |
| N_a_ | The size of the ancestral population to Tibetans and Hans | 10,206 | 9,987 – 10,425 | N_a_ | The size of the ancestral population to Yoruba samples in Ibadan (YRI) and East Asians (EAS) | 12,495 | 11,931 – 13,059 |
| theta | Mutation rates per 4N_a_ generations | 98,384 | 96,273 – 100,494 | theta | Mutation rates per 4N_a_ generations | 130,826 | 124,917 – 136,734 |
| N_TIB_ | The ancestral population size of Tibetans | 36,260 | 36,234 – 36,285 | N_YRI_ | The ancestral population size of YRI | 28,885 | 28,863 – 28,906 |
| N_HAN_ | The ancestral population size of Hans | 50,870 | 50,647 – 51,092 | N_EAS_ | The ancestral population size of EAS | 8,906 | 8,855 – 9,435 |
| N_HANN_ | The ancestral population size of Han in the North (HANN) | 50,667 | 50,485 – 50,848 | N_TIB_ | The ancestral population size of Tibetans | 34657 | 34,605 – 34,776 |
| N_HANS_ | The ancestral population size of Han in the South (HANS) | 1,632 | 1,626 – 1,637 | N_HAN_ | The ancestral population size of Hans | 43,446 | 43,435 – 43,453 |
| T1 | The divergence time between Tibetans and Hans | 16,871 | 14,049 – 19,692 | T1 | The divergence time between YRI and EAS | 65,662 | 63,109 – 68,214 |
| T2 | The divergence time between HANN and HANS | 769 | 727 – 810 | T2 | The divergence time between Tibetans and Hans | 22,974 | 19,763 – 26,184 |
| M_TIB-HAN_ | Migration rate between Tibetans and Hans per 2N_a_ generations | 0.0016 | 0 – 0.0032 | M_YRI-EAS_ | Migration rate between YRI and EAS per 2N_a_ generations | 0.0166 | 0.0138 – 0.0193 |
| M_TIB-HANN_ | Migration rate between Tibetans and HANN per 2N_a_ generations | 0.0087 | 0.0082 – 0.0091 | M_YRI-TIB_ | Migration rate between YRI and Tibetans per 2N_a_ generations | 0.0983 | 0.0955 – 0.1010 |
| M_HANN-HANS_ | Migration rate between HANN and HANS per 2N_a_ generations | 0.0537 | 0.0432 – 0.0641 | M_TIB-HAN_ | Migration rate between Tibetans and Hans per 2N_a_ generations | 0.0072 | 0.0054 – 0.0089 |
| M_TIB-HANS_ | Migration rate between Tibetans and HANS per 2N_a_ generations | 0.003 | 0 – 0.0175 | M_YRI-HAN_ | Migration rate between YRI and Hans per 2N_a_ generations | 0.001 | 0.0002 – 0.0017 |

All scaled parameter estimates were transformed into real values using a mutation rate of 1.5 × 10^-8^ per site per generation and a generation time of 29 years

**Table S9. Primers used for PCR-Sanger sequencing.**

| **SVID** | **Types** | **Sequences (5'→3')** | **Validation** |
| --- | --- | --- | --- |
| chr1_158488405_158488672_DEL | Forward | GTTGGATACTTTCAGACTGGTTC | PASS |
|  | Reverse | AGAGCACATCCAGAAATCCTAG |  |
| chr22_40935457_40935620_DEL | Forward | CTCCCAGGTTCAAGTGATTC | FAIL |
|  | Reverse | TGTAATCCCAGCACTTTGG |  |
| chr9_81725770_81726090_DEL | Forward | ATAACTCTCTGCCACTCCC | PASS |
|  | Reverse | TGCCTACGACTTGGAGTT |  |
| chr7_127766419_127766732_DEL | Forward | CCTCTTTCACTTCCTGGA | PASS |
|  | Reverse | AAACTACAGCCACTTCCACT |  |
| chr18_45099900_45100212_DEL | Forward | ATCACCTTCCACCATCCA | PASS |
|  | Reverse | TCCTTCACCTCCAGTTACAGA |  |
| chr21_42402890_42403212_DEL | Forward | CCTTGCCAGCATCTGTTAT | PASS |
|  | Reverse | CCAGTTTAGAATGAGAGGTGG |  |
| chr12_52354758_52354891_DEL | Forward | AGCAGTGGGTCCAAAGTCT | PASS |
|  | Reverse | GCAACTGGGACAATGGAA |  |
| chr5_52231784_52232117_DEL | Forward | AGAAGGCACATTTTGGAGTAAG | PASS |
|  | Reverse | GGGCAGGATAAAGTTCAACA |  |
| chr20_15435298_15435464_DEL | Forward | TGCCTTACATCCTGCCCTA | PASS |
|  | Reverse | ATTCTCGCTGTTGGCTACG |  |
| chr14_22881662_22882256_DEL | Forward | CACAATAGCCTTCACTTGGA | PASS |
|  | Reverse | AGAACCCAGGGAAAGAGAAT |  |
| chr1_225272502_225272503_INS | Forward | TGGAGGATTGACCCAGAGGT | FAIL |
|  | Reverse | TGCCCTAATGCCAACTGCT |  |
| chr11_48928944_48929254_DEL | Forward | ATCACTGGACTGAGGGACA | PASS |
|  | Reverse | AACTGCTGTTGATGACTGGA |  |
| chr17_693876_694043_DEL | Forward | CCAATGGGTCTTGATGAAAC | PASS |
|  | Reverse | GCACCCACACAGTCATAGAA |  |
| chr6_100066805_100066874_DEL | Forward | CGGAGTTACGGGAGTAGTGA | PASS |
|  | Reverse | CCTCCTCTTCACAACTCACAC |  |
| chr2_28661655_28661709_DEL | Forward | ATCCTCGCATTCCAGATG | PASS |
|  | Reverse | CAGAACAGGGAAGGTAGGATT |  |
| chr10_97992045_97992148_DEL | Forward | TGCCCAAGGTCAGATAGTG | PASS |
|  | Reverse | CCATTGTGCCAGGTAATCA |  |

**Table S15. Uniform priors for demographic parameters relevant to events prior to anatomically modern humans.**

| **Parameters** | **Range** |
| --- | --- |
| The size of the ancestral population to chimpanzee and human (N_chimp-hominin_) | unif(50,000, 100,000) |
| The contemporary population size of chimpanzee (N_chimp_) | unif(10,000, 50,000) |
| The size of ancestral population to hominin (N_hominin_) | unif(10,000, 20,000) |
| The ancestral population size of Denisovans, Neanderthals, and their common ancestors (N_ARC_) | unif(2,000, 4,000) |
| The divergence time between chimpanzee and human (T_chimp-hominin_) | unif(5,000,000, 10,000,000) |
| The divergence time between archaic hominins and humans (T_ARC-AMH_) | unif(522,000, 634,000) |
| The divergence time between Denisovan and Neanderthal (T_DNS-NDL_) | unif(392,000, 438,000) |
| The divergence time between Siberian and European Neanderthal (T_Altai-Vindija_) | unif(130,000, 145,000) |

This table was extracted from Hsieh, et al. 2019. Time in years and population sizes are the numbers of individuals

**Table S16. Summary of structural variations (SVs) with signatures of both selection (p-value < 0.05 for iHS) and introgression (p-value < 0.05 for both f_d_-statistic and S^*^-like statistic).**

| **No.** | **SV Location (GRCh37)** | **Type** | **Genotyped allele frequency** | | | | | **iHS**  **(p-value)** | **f_d_ (p-value)** | | **S^*^-like statistic**  **(p-value)** |
| --- | --- | --- | --- | --- | --- | --- | --- | --- | --- | --- | --- |
|  |  |  | **Tibetans** | **Hans** | **Biaka** | **DNS** | **NDL** |  | **DNS** | **NDL** |  |
| 1 | chr7:1,185,068-1,187,658 | Deletion | 0.78 | 0.45 | 0.92 | **1.00** | **0.50** | 0.028 | 0.025 | 0.001 | <0.001 |
| 2 | chr18:45,099,900-45,100,212 | Deletion | 0.24 | 0.02 | 0.48 | 0.00 | 0.00 | <0.001 | NS | 0.049 | <0.001 |
| 3 | chr21:42,402,890-42,403,212 | Deletion | 0.71 | 0.39 | 1.00 | 0.00 | **0.25** | 0.045 | 0.046 | 0.001 | <0.001 |
| 4 | chr12:52,354,758-52,354,891 | Deletion | 0.61 | 0.28 | 0.04 | 0.00 | 0.00 | 0.049 | 0.019 | 0.039 | <0.001 |
| 5 | chr1:2,919,030-2,919,365 | Deletion | 0.06 | 0.23 | 0.00 | **0.50** | **0.50** | 0.026 | NS | 0.016 | <0.001 |
| 6 | chr6:95,400,827-95,400,920 | Deletion | 0.93 | 0.74 | 0.19 | 0.00 | 0.00 | 0.006 | NS | 0.010 | <0.001 |

DNS, Denisovans; NDL, Neandertals; iHS, integrated haplotype homozygosity score; f_d_, f_d_-statistic which is designed to distinguish excess genetic drift from ancient introgression based on allele frequencies; NS, not significant.

**Table S21. Primers used for luciferase reporter gene assay.**

| **Names** | **Types** | **Sequences (5'→3')** | **Targets** | **Restriction sites** | **Vectors** |
| --- | --- | --- | --- | --- | --- |
| **For gene cloning assay** | | | | | |
| PRDM16-promoter | Forward | GGTACCAGAGCCGGGCCTGTAAACT | *PRDM16* promoter | *Kpn* I and *Nhe* I | pGL3 basic |
|  | Reverse | GCTAGCTTTGGACACCTTCGCACATG |  |  |  |
| ACTRT2-promoter | Forward | GGTACCGCAGGTCAGCAGGGATGG | *ACTRT2* promoter | *Kpn* I and *Nhe* I | pGL3 basic |
|  | Reverse | ATAATAGCTAGCGGGGGCCAGGGGGTTGT |  |  |  |
| 1p36.32 WT | Forward | ATAATAGAGCTCGCCCACATTCTCGGACGC | 1p36.32 DEL-centered region | *Sac* I and *Xho* I | pGL3 promoter |
|  | Reverse | ATAATACTCGAGCCCAGCGAGAGCTGTGCA |  |  |  |
| **For site-directed mutagenesis** | | | | | |
| 1p36.32 DEL | Forward | CCGATCCCAGTCCCGATGGGACACAGAGACCTCCCCG | Region surrounding 1p36.32 DEL | NA | pGL3 promoter |
|  | Reverse | AGGTCTCTGTGTCCCATCGGGACTGGGATCGGTGCCTG |  |  |  |

**Table S22. Primers used for quantitative real-time PCR assays.**

| **Genes** | **Types** | **Sequences (5'→3')** |
| --- | --- | --- |
| *PRDM16* | Forward | CGAGGCCCCTGTCTACATTC |
|  | Reverse | GCTCCCATCCGAAGTCTGTC |
| *ACTRT2* | Forward | GACTCCCCGGCTGTGATTTTT |
|  | Reverse | ATCAGGCCACGCTCGAAAG |
| *CEP104* | Forward | TGCAGTTACTTGCTCACCAGT |
|  | Reverse | TCTGCTTGATAGGGTGCAAAAT |
| *LRRC47* | Forward | GCAGCGGTGTCAGAGTCTT |
|  | Reverse | CACTTCCAAGTAGTGCAGCAG |
| *ACTB* | Forward | AGAGCCTCGCCTTTGCCGAT |
|  | Reverse | CCATCACGCCCTGGTGCCT |

**Table S23. Description of 51 quantitative traits used for phenotype association analysis with the 335 bp deletion at 1p36.32.**

| **Quantitative traits** | **Descriptions** | **Association coefficient** | **p-value** | **Sample size** | **FDR** |
| --- | --- | --- | --- | --- | --- |
| ALT | Alanine Aminotransferase | 0.137 | 0.006 | 396 | 0.319 |
| APOA1 | Apolipoprotein A1 | -0.148 | 0.124 | 110 | 0.626 |
| APOB | Apolipoprotein B | -0.121 | 0.207 | 110 | 0.641 |
| AST | Aspartate aminotransferase | 0.072 | 0.152 | 396 | 0.626 |
| CHO | Carbohydrate | -0.162 | 0.092 | 110 | 0.626 |
| CK | Creatine Kinase | -0.045 | 0.644 | 110 | 0.959 |
| CKMB | Creatine kinase-MB | -0.070 | 0.472 | 109 | 0.925 |
| HBDH | hydroxybutyrate dehydrogenase | -0.015 | 0.876 | 110 | 0.959 |
| HDL | High-density lipoprotein | -0.157 | 0.100 | 110 | 0.626 |
| LDH | Lactate dehydrogenase | -0.058 | 0.550 | 110 | 0.959 |
| LDL | Low-density lipoprotein | -0.131 | 0.171 | 110 | 0.626 |
| TG | Triglyceride | 0.064 | 0.506 | 110 | 0.957 |
| WBC | White blood cell | -0.012 | 0.810 | 411 | 0.959 |
| HGB | Hemoglobin | 0.010 | 0.839 | 411 | 0.959 |
| RBC | Red blood cells | 0.023 | 0.646 | 411 | 0.959 |
| HCT | Hematocrit | 0.007 | 0.895 | 411 | 0.959 |
| MCV | Mean corpuscular volume | -0.015 | 0.767 | 411 | 0.959 |
| MCH | Mean corpuscular hemoglobin | -0.005 | 0.919 | 411 | 0.959 |
| MCHC | Mean corpuscular hemoglobin concentration | 0.006 | 0.910 | 411 | 0.959 |
| RDW-CV | Red Blood Cell Distribution Width | -0.030 | 0.540 | 411 | 0.959 |
| RDW-SD | Red Blood Cell Distribution Width (sd) | -0.098 | 0.047 | 411 | 0.478 |
| PLT | Platelet count | 0.008 | 0.871 | 411 | 0.959 |
| MPV | Mean platelet volume | 0.063 | 0.219 | 380 | 0.641 |
| PDW | Platelet distribution width | 0.027 | 0.605 | 380 | 0.959 |
| PCT | Thrombocytocrit | 0.070 | 0.172 | 380 | 0.626 |
| P-LCR | Platelet large cell ratio | 0.069 | 0.189 | 366 | 0.641 |
| EO% | Eosinophils% | -0.041 | 0.455 | 339 | 0.925 |
| EO# | Eosinophils# | -0.066 | 0.227 | 338 | 0.641 |
| MONO% | Mononuclear% | -0.056 | 0.297 | 353 | 0.722 |
| MONO# | Mononuclear# | -0.124 | 0.020 | 353 | 0.408 |
| LYMPH% | Lymphocyte% | 0.062 | 0.251 | 339 | 0.641 |
| LYMPH# | Lymphocyte# | -0.023 | 0.676 | 339 | 0.959 |
| NEUT% | neutrophil% | -0.017 | 0.752 | 353 | 0.959 |
| NEUT# | neutrophil# | -0.078 | 0.145 | 353 | 0.626 |
| BASO% | Basophil% | 0.026 | 0.631 | 351 | 0.959 |
| BASO# | Basophil# | -0.001 | 0.982 | 350 | 0.988 |
| GLO | Globulin | -0.081 | 0.150 | 320 | 0.626 |
| IBIL | Indirect bilirubin | -0.006 | 0.922 | 320 | 0.959 |
| DBIL | Direct bilirubin | -0.023 | 0.667 | 340 | 0.959 |
| ADA | Adenosine deaminase | -0.048 | 0.378 | 340 | 0.876 |
| BUN | Blood Urea Nitrogen | 0.007 | 0.899 | 340 | 0.959 |
| TP | Total protein | 0.006 | 0.912 | 340 | 0.959 |
| ALB | Albumin | 0.110 | 0.043 | 340 | 0.478 |
| UA | Uric acid | -0.009 | 0.867 | 340 | 0.959 |
| TBIL | Total bilirubin | -0.010 | 0.848 | 340 | 0.959 |
| GGT | Gamma-glutamyl transpeptidase | 0.064 | 0.242 | 340 | 0.641 |
| ALP | Alkaline phosphatase | -0.043 | 0.426 | 340 | 0.925 |
| MAO | monoamine oxidase | -0.042 | 0.444 | 340 | 0.925 |
| CREA | Creatinine | 0.001 | 0.988 | 340 | 0.988 |
| ALB/GLO | Albumin / Globulin | 0.126 | 0.024 | 320 | 0.408 |
| ALT/AST | Alanine Aminotransferase / Aspartate aminotransferase | 0.090 | 0.098 | 340 | 0.626 |

**Table S24. Correlation of genotypes for structural variations in 15 Tibetan samples between next-generation sequencing and nanopore sequencing**

| **SampleID** | **Correlation Coefficient** | **95 percent confidence interval** | **df** | **p-value** |
| --- | --- | --- | --- | --- |
| CQ082 | 0.385 | [0.375 0.394] | 28,610 | < 2.2e-16 |
| CQ085 | 0.384 | [0.374 0.394] | 28,599 | < 2.2e-16 |
| CQ091 | 0.393 | [0.383 0.403] | 29,301 | < 2.2e-16 |
| CQ095 | 0.381 | [0.372 0.391] | 28,488 | < 2.2e-16 |
| CQ104 | 0.39 | [0.380 0.400] | 28,571 | < 2.2e-16 |
| CQ115 | 0.384 | [0.374 0.394] | 28,521 | < 2.2e-16 |
| CQ121 | 0.393 | [0.383 0.403] | 27,722 | < 2.2e-16 |
| CQ123 | 0.379 | [0.369 0.389] | 28,872 | < 2.2e-16 |
| CQ132 | 0.404 | [0.394 0.413] | 28,782 | < 2.2e-16 |
| CQ136 | 0.388 | [0.378 0.398] | 28,076 | < 2.2e-16 |
| CQ143 | 0.39 | [0.380 0.399] | 29,287 | < 2.2e-16 |
| CQ144 | 0.391 | [0.381 0.401] | 28,836 | < 2.2e-16 |
| CQ152 | 0.384 | [0.375 0.394] | 29,450 | < 2.2e-16 |
| CQ188 | 0.381 | [0.371 0.391] | 28,786 | < 2.2e-16 |
| CQ200 | 0.368 | [0.358 0.378] | 27,630 | < 2.2e-16 |

# References

1. Lu D, Lou H, Yuan K, Wang X, Wang Y, Zhang C, Lu Y, Yang X, Deng L, Zhou Y, et al. Ancestral Origins and Genetic History of Tibetan Highlanders. Am J Hum Genet*.* 2016;99**:**580-94. <https://doi.org/10.1016/j.ajhg.2016.07.002>.

2. Lan T, Lin H, Zhu W, Laurent TCAM, Yang M, Liu X, Wang J, Wang J, Yang H, Xu X, Guo X. Deep whole-genome sequencing of 90 Han Chinese genomes. Gigascience*.* 2017;6**:**gix067. <https://doi.org/10.1093/gigascience/gix067>.

3. Meyer M, Kircher M, Gansauge M-T, Li H, Racimo F, Mallick S, Schraiber JG, Jay F, Prüfer K, Filippo Cd, et al. A High-Coverage Genome Sequence from an Archaic Denisovan Individual. Science*.* 2012;338**:**222-6. <https://doi.org/10.1126/science.1224344>.

4. Prüfer K, Racimo F, Patterson N, Jay F, Sankararaman S, Sawyer S, Heinze A, Renaud G, Sudmant PH, Filippo Cd, et al. The complete genome sequence of a Neanderthal from the Altai Mountains. Nature*.* 2014;505**:**43-9. <https://doi.org/10.1038/nature12886>.

5. Prüfer K, Filippo Cd, Grote S, Mafessoni F, Korlević P, Hajdinjak M, Vernot B, Skov L, Hsieh P, Peyrégne S, et al. A high-coverage Neandertal genome from Vindija Cave in Croatia. Science*.* 2017;358**:**655-8. <https://doi.org/10.1126/science.aao1887>.

6. Prado-Martinez J, Sudmant PH, Kidd JM, Li H, Kelley JL, Lorente-Galdos B, Veeramah KR, Woerner AE, O’Connor TD, Santpere G, et al. Great ape genetic diversity and population history. Nature*.* 2013;499**:**471-5. <https://doi.org/10.1038/nature12228>.

7. Sedlazeck FJ, Rescheneder P, Smolka M, Fang H, Nattestad M, Haeseler Av, Schatz MC. Accurate detection of complex structural variations using single-molecule sequencing. Nat Methods*.* 2018;15**:**461-8. <https://doi.org/10.1038/s41592-018-0001-7>.

8. Stancu M, Roosmalen MJv, Renkens I, Nieboer MM, Middelkamp S, Ligt Jd, Pregno G, Giachino D, Mandrile G, Valle-Inclan J, et al. Mapping and phasing of structural variation in patient genomes using nanopore sequencing. Nat Commun*.* 2017;8**:**1326. <https://doi.org/10.1038/s41467-017-01343-4>.

9. Heller D, Vingron M. SVIM: Structural Variant Identification using Mapped Long Reads. Bioinformatics*.* 2019;35**:**2907–15. <https://doi.org/10.1093/bioinformatics/btz041>.

10. Coster W, Rijk P, Roeck A, Pooter T, D'Hert S, Strazisar M, Sleegers K, Broeckhoven C. Structural variants identified by Oxford Nanopore PromethION sequencing of the human genome. Genome Res*.* 2019;29**:**1178-87. <https://doi.org/10.1101/gr.244939.118>.

11. Jeffares DC, Jolly C, Hoti M, Speed D, Shaw L, Rallis C, Balloux F, Dessimoz C, Bähler J, Sedlazeck FJ. Transient structural variations have strong effects on quantitative traits and reproductive isolation in fission yeast. Nat Commun*.* 2017;8**:**14061. <https://doi.org/10.1038/ncomms14061>.

12. Geoffroy V, Herenger Y, Kress A, Stoetzel C, Piton A, Dollfus H, Muller J. AnnotSV: an integrated tool for structural variations annotation. Bioinformatics*.* 2018;34**:**3572-4. <https://doi.org/10.1093/bioinformatics/bty304>.

13. Collins RL, Brand H, Karczewski KJ, Zhao X, Alföldi J, Francioli LC, Khera AV, Lowther C, Gauthier LD, Wang H, et al. A structural variation reference for medical and population genetics. Nature*.* 2020;581**:**444-51. <https://doi.org/10.1038/s41586-020-2287-8>.

14. Sudmant PH, Rausch T, Gardner EJ, Handsaker RE, Abyzov A, Huddleston J, Zhang Y, Ye K, Jun G, Fritz M, et al. An integrated map of structural variation in 2,504 human genomes. Nature*.* 2015;526**:**75. <https://doi.org/10.1038/nature15394>.

15. Abel HJ, Larson DE, Regier AA, Chiang C, Das I, Kanchi KL, Layer RM, Neale BM, Salerno WJ, Reeves C, et al. Mapping and characterization of structural variation in 17,795 human genomes. Nature*.* 2020;583**:**83-9. <https://doi.org/10.1038/s41586-020-2371-0>.

16. Ouzhuluobu, He Y, Lou H, Cui C, Deng L, Gao Y, Zheng W, Guo Y, Wang X, Ning Z, et al. De novo assembly of a Tibetan genome and identification of novel structural variants associated with high altitude adaptation. bioRxiv*.* 2019**:**753186. <https://doi.org/10.1101/753186>.

17. Shi L, Guo Y, Dong C, Huddleston J, Yang H, Han X, Fu A, Li Q, Li N, Gong S, et al. Long-read sequencing and de novo assembly of a Chinese genome. Nat Commun*.* 2016;7**:**12065. <https://doi.org/10.1038/ncomms12065>.

18. Audano PA, Sulovari A, Graves-Lindsay TA, Cantsilieris S, Sorensen M, Welch AE, Dougherty ML, Nelson BJ, Shah A, Dutcher SK, et al. Characterizing the Major Structural Variant Alleles of the Human Genome. Cell*.* 2019;176**:**663-75.e19. <https://doi.org/10.1016/j.cell.2018.12.019>.

19. Quinlan AR, Hall IM. BEDTools: a flexible suite of utilities for comparing genomic features. Bioinformatics*.* 2010;26**:**841-2. <https://doi.org/10.1093/bioinformatics/btq033>.

20. Jurka J. Repbase Update: a database and an electronic journal of repetitive elements. Trends Genet*.* 2000;16**:**418-20. <https://doi.org/10.1016/s0168-9525(00)02093-x>.

21. Lam HYK, Mu XJ, Stütz AM, Tanzer A, Cayting PD, Snyder M, Kim PM, Korbel JO, Gerstein MB. Nucleotide-resolution analysis of structural variants using BreakSeq and a breakpoint library. Nat Biotechnol*.* 2009;28**:**47-55. <https://doi.org/10.1038/nbt.1600>.

22. Hubbard TJP, Aken BL, Ayling S, Ballester B, Beal K, Bragin E, Brent S, Chen Y, Clapham P, Clarke L, et al. Ensembl 2009. Nucleic Acids Res*.* 2008;37**:**D690-7. <https://doi.org/10.1093/nar/gkn828>.

23. Werling DM, Brand H, An J-Y, Stone MR, Zhu L, Glessner JT, Collins RL, Dong S, Layer RM, Markenscoff-Papadimitriou E, et al. An analytical framework for whole-genome sequence association studies and its implications for autism spectrum disorder. Nat Genet*.* 2018;50**:**727-36. <https://doi.org/10.1038/s41588-018-0107-y>.

24. Lek M, Karczewski KJ, Minikel EV, Samocha KE, Banks E, Fennell T, O'Donnell-Luria AH, Ware JS, Hill AJ, Cummings BB, et al. Analysis of protein-coding genetic variation in 60,706 humans. Nature*.* 2016;536**:**285-91. <https://doi.org/10.1038/nature19057>.

25. Firth HV, Richards SM, Bevan AP, Clayton S, Corpas M, Rajan D, Vooren SV, Moreau Y, Pettett RM, Carter NP. DECIPHER: Database of Chromosomal Imbalance and Phenotype in Humans Using Ensembl Resources. Am J Hum Genet*.* 2009;84**:**524-33. <https://doi.org/10.1016/j.ajhg.2009.03.010>.

26. Fudenberg G, Pollard KS. Chromatin features constrain structural variation across evolutionary timescales. Proc Natl Acad Sci*.* 2019;116**:**201808631. <https://doi.org/10.1073/pnas.1808631116>

27. Dunham I, Kundaje A, Aldred SF, Collins PJ, Davis CA, Doyle F, Epstein CB, Frietze S, Harrow J, Kaul R, et al. An integrated encyclopedia of DNA elements in the human genome. Nature*.* 2012;489**:**57-74. <https://doi.org/10.1038/nature11247>.

28. Kundaje A, Meuleman W, Ernst J, Bilenky M, Yen A, Heravi-Moussavi A, Kheradpour P, Zhang Z, Wang J, Ziller MJ, et al. Integrative analysis of 111 reference human epigenomes. Nature*.* 2015;518**:**317-30. <https://doi.org/10.1038/nature14248>.

29. Coster W, Broeckhoven C. Newest Methods for Detecting Structural Variations. Trends Biotechnol*.* 2019;37**:**973-82. <https://doi.org/10.1016/j.tibtech.2019.02.003>.

30. Chen S, Krusche P, Dolzhenko E, Sherman RM, Petrovski R, Schlesinger F, Kirsche M, Bentley DR, Schatz MC, Sedlazeck FJ, Eberle MA. Paragraph: a graph-based structural variant genotyper for short-read sequence data. Genome Biol*.* 2019;20**:**291. <https://doi.org/10.1186/s13059-019-1909-7>.

31. Pedersen BS, Quinlan AR. Mosdepth: quick coverage calculation for genomes and exomes. Bioinformatics*.* 2017;34**:**867-8. <https://doi.org/10.1093/bioinformatics/btx699>.

32. Talevich E, Shain AH, Botton T, Bastian BC. CNVkit: Genome-Wide Copy Number Detection and Visualization from Targeted DNA Sequencing. Plos Comput Biol*.* 2016;12**:**e1004873. <https://doi.org/10.1371/journal.pcbi.1004873>.

33. Delaneau O, Zagury J-F, Robinson MR, Marchini JL, Dermitzakis ET. Accurate, scalable and integrative haplotype estimation. Nat Commun*.* 2019;10**:**5436. <https://doi.org/10.1038/s41467-019-13225-y>.

34. Graffelman J. Exploring diallelic genetic markers: the hardy weinberg package. J Stat Softw*.* 2015;64**:**1-23. <https://doi.org/10.18637/jss.v064.i03>.

35. Jakubosky D, D’Antonio M, Bonder M, Smail C, Donovan M, Greenwald WW, D’Antonio-Chronowska A, Matsui H, Consortium iQTL, Stegle O, et al. Genomic properties of structural variants and short tandem repeats that impact gene expression and complex traits in humans. bioRxiv*.* 2019**:**714477. <https://doi.org/10.1101/714477>.

36. Weir BS, Cockerham CC. Estimating F-Statistics for the Analysis of Population Structure. Evol Int J Org Evol*.* 1984;38**:**1358-70. <https://doi.org/10.1111/j.1558-5646.1984.tb05657.x>.

37. GTEx Consortium. The GTEx Consortium atlas of genetic regulatory effects across human tissues. Science*.* 2020;369**:**1318-30. <https://doi.org/10.1126/science.aaz1776>.

38. Hormozdiari F, Kostem E, Kang EY, Pasaniuc B, Eskin E. Identifying causal variants at loci with multiple signals of association. Genetics*.* 2014;198**:**497-508. <https://doi.org/10.1534/genetics.114.167908>.

39. Chiang C, Scott AJ, Davis JR, Tsang EK, Li X, Kim Y, Hadzic T, Damani FN, Ganel L, Consortium G, et al. The impact of structural variation on human gene expression. Nat Genet*.* 2017;49**:**692-9. <https://doi.org/10.1038/ng.3834>.

40. Gokhman D, Kelman G, Amartely A, Gershon G, Tsur S, Carmel L. Gene ORGANizer: linking genes to the organs they affect. Nucleic Acids Res*.* 2017;45**:**W138-W45. <https://doi.org/10.1093/nar/gkx302>.

41. Shin J-H, Blay S, Graham J, McNeney B. LDheatmap : An R Function for Graphical Display of Pairwise Linkage Disequilibria Between Single Nucleotide Polymorphisms. J Stat Softw*.* 2006;16. <https://doi.org/10.18637/jss.v016.c03>.

42. Wlasnowolski M, Sadowski M, Czarnota T, Jodkowska K, Szalaj P, Tang Z, Ruan Y, Plewczynski D. 3D-GNOME 2.0: a three-dimensional genome modeling engine for predicting structural variation-driven alterations of chromatin spatial structure in the human genome. Nucleic Acids Res*.* 2020;48**:**W170-W6. <https://doi.org/10.1093/nar/gkaa388>.

43. Sadowski M, Kraft A, Szalaj P, Wlasnowolski M, Tang Z, Ruan Y, Plewczynski D. Spatial chromatin architecture alteration by structural variations in human genomes at the population scale. Genome Biol*.* 2019;20**:**148. <https://doi.org/10.1186/s13059-019-1728-x>.

44. Rao Suhas SP, Huntley Miriam H, Durand Neva C, Stamenova Elena K, Bochkov Ivan D, Robinson James T, Sanborn Adrian L, Machol I, Omer Arina D, Lander Eric S, Aiden Erez L. A 3D Map of the Human Genome at Kilobase Resolution Reveals Principles of Chromatin Looping. Cell*.* 2015;162**:**687-8. <https://doi.org/10.1016/j.cell.2015.07.024>.

45. Gu Z, Gu L, Eils R, Schlesner M, Brors B. circlize implements and enhances circular visualization in R. Bioinformatics*.* 2014;30**:**2811-2. <https://doi.org/10.1093/bioinformatics/btu393>.

46. McLaren W, Gil L, Hunt SE, Riat H, Ritchie GRS, Thormann A, Flicek P, Cunningham F. The Ensembl Variant Effect Predictor. Genome Biol*.* 2016;17**:**122. <https://doi.org/10.1186/s13059-016-0974-4>.

47. Zhou Y, Zhou B, Pache L, Chang M, Khodabakhshi AH, Tanaseichuk O, Benner C, Chanda SK. Metascape provides a biologist-oriented resource for the analysis of systems-level datasets. Nat Commun*.* 2019;10**:**1523. <https://doi.org/10.1038/s41467-019-09234-6>.

48. Deng L, Zhang C, Yuan K, Gao Y, Pan Y, Ge X, He Y, Yuan Y, Lu Y, Zhang X, et al. Prioritizing natural selection signals from the deep-sequencing genomic data suggests multi-variant adaptation in Tibetan highlanders. Natl Sci Rev*.* 2019;6**:**1201–22. <https://doi.org/10.1093/nsr/nwz108>.

49. Hsieh P, Vollger MR, Dang V, Porubsky D, Baker C, Cantsilieris S, Hoekzema K, Lewis AP, Munson KM, Sorensen M, et al. Adaptive archaic introgression of copy number variants and the discovery of previously unknown human genes. Science*.* 2019;366**:**eaax2083. <https://doi.org/10.1126/science.aax2083>.

50. Hsieh P, Veeramah KR, Lachance J, Tishkoff SA, Wall JD, Hammer MF, Gutenkunst RN. Whole-genome sequence analyses of Western Central African Pygmy hunter-gatherers reveal a complex demographic history and identify candidate genes under positive natural selection. Genome Res*.* 2016;26**:**279-90. <https://doi.org/10.1101/gr.192971.115>.

51. Liu X, Zhang Y, Li Y, Pan J, Wang D, Chen W, Zheng Z, He X, Zhao Q, Pu Y, et al. EPAS1 gain-of-function mutation contributes to high-altitude adaptation in Tibetan horses. Mol Biol Evol*.* 2019. <https://doi.org/10.1093/molbev/msz158>.

52. Gutenkunst RN, Hernandez RD, Williamson SH, Bustamante CD. Inferring the joint demographic history of multiple populations from multidimensional SNP frequency data. Plos Genet*.* 2009;5**:**e1000695. <https://doi.org/10.1371/journal.pgen.1000695>.

53. Portik DM, Leaché AD, Rivera D, Barej MF, Burger M, Hirschfeld M, Rödel M-O, Blackburn DC, Fujita MK. Evaluating mechanisms of diversification in a Guineo-Congolian tropical forest frog using demographic model selection. Mol Ecol*.* 2017;26**:**5245-63. <https://10.1111/mec.14266>.

54. Coffman AJ, Hsieh P, Gravel S, Gutenkunst RN. Computationally Efficient Composite Likelihood Statistics for Demographic Inference. Mol Biol Evol*.* 2015;33**:**591-3. <https://doi.org/10.1093/molbev/msv255>.

55. Scally A. The mutation rate in human evolution and demographic inference. Curr Opin Genet Dev*.* 2016;41**:**36-43. <https://doi.org/10.1016/j.gde.2016.07.008>.

56. Kelleher J, Etheridge AM, McVean G. Efficient Coalescent Simulation and Genealogical Analysis for Large Sample Sizes. Plos Comput Biol*.* 2016;35**:**2907–15. <https://doi.org/10.1371/journal.pcbi.1004842>.

57. Frazer KA, Ballinger DG, Cox DR, Hinds DA, Stuve LL, Gibbs RA, Belmont JW, Boudreau A, Hardenbol P, Leal SM, et al. A second generation human haplotype map of over 3.1 million SNPs. Nature*.* 2007;449**:**851-61. <https://doi.org/10.1038/nature06258>.

58. Watterson GA. On the number of segregating sites in genetical models without recombination. Theor Popul Biol*.* 1975;7**:**256-76. <https://doi.org/10.1016/0040-5809(75)90020-9>.

59. Voight BF, Kudaravalli S, Wen X, Pritchard JK. A Map of Recent Positive Selection in the Human Genome. Plos Biol*.* 2006;4**:**e72. <https://doi.org/10.1371/journal.pbio.0040072>.

60. Sabeti PC, Varilly P, Fry B, Lohmueller J, Hostetter E, Cotsapas C, Xie X, Byrne EH, McCarroll SA, Gaudet R, et al. Genome-wide detection and characterization of positive selection in human populations. Nature*.* 2007;449**:**913-8. <https://doi.org/10.1038/nature06250>.

61. Gautier M, Vitalis R. rehh: an R package to detect footprints of selection in genome-wide SNP data from haplotype structure. Bioinformatics*.* 2012;28**:**1176-7. <https://doi.org/10.1093/bioinformatics/bts115>.

62. Petr M, Vernot B, Kelso J. admixr —R package for reproducible analyses using ADMIXTOOLS. Bioinformatics*.* 2019. <https://doi.org/10.1093/bioinformatics/btz030>.

63. Browning SR, Browning BL, Zhou Y, Tucci S, Akey JM. Analysis of Human Sequence Data Reveals Two Pulses of Archaic Denisovan Admixture. Cell*.* 2018;173**:**53-61.e9. <https://doi.org/10.1016/j.cell.2018.02.031>.

64. Plagnol V, Wall JD. Possible ancestral structure in human populations. PLoS Genet*.* 2006;2**:**e105. <https://doi.org/10.1371/journal.pgen.0020105>.

65. Leigh JW, Bryant D. PopART: Full-feature software for haplotype network construction. Methods Ecol Evol*.* 2015;6**:**1110-6. <https://doi.org/10.1111/2041-210X.12410>.

66. Yang J, Jin Z-B, Chen J, Huang X-F, Li X-M, Liang Y-B, Mao J-Y, Chen X, Zheng Z, Bakshi A, et al. Genetic signatures of high-altitude adaptation in Tibetans. Proc Natl Acad Sci*.* 2017;114**:**4189-94. <https://doi.org/10.1073/pnas.1617042114>.
