# Supplementary material for: Characterization of structural variation in Tibetans reveals new evidence of high-altitude adaptation and introgression
Source: Genome Biol. 2021 May 25;22:159. doi: 10.1186/s13059-021-02382-3 (PMC8146648; doi:10.1186/s13059-021-02382-3)
Supplement: Supplementary file 14 — Additional file 14. Review history. [file 13059_2021_2382_MOESM14_ESM.docx]

Review History

**First round of review**

**Reviewer 1**

**Are you able to assess all statistics in the manuscript, including the appropriateness of statistical tests used?**

Yes, and I have assessed the statistics in my report.

**Comments to author:**

The authors employed long read sequencing technology to characterize the SVs in Tibetan and Han Chinese populations. They further combined the short read data to genotype and search for population-stratified SVs. Finally, they attempted to identify the SVs under natural selection and the ones that were introgressed from archaic hominid. The data of this study would be a good resource for future studies, but the findings in terms of novelty are relatively limited compared with the already known. In particular, I find most conclusions on the evolutionary processes are mostly concordance with previous studies based on SNVs. On the other hand, despite that massive analyses were performed, some of the results are not sufficient or solid to support the major conclusions. I have the quite a few concerns which I hope the authors are able to address in the revised version.

Major issues

(1)	Ouzhuluobu et al. 2020 has already used PacBio long-read sequencing (LRS) technology to report a comprehensive SV map in Tibetan (ZF1), and other studies have used PacBio LRS in Han Chinese such as HX1 (Shi et al. 2016) and NH1 (Du et al. 2019), as well as those discovered in 15 worldwide samples (Audano et al. 2019), but the authors didn't compare their findings with any of the LRS studies. How many of the SVs in this study are novel if including those LRS SV studies in the comparison? Besides, Ouzhuluobu et al. 2020 provided a candidate list of adaptive SVs, it is unclear about which adaptive signals are novel.

(2)	The authors didn't mention which sequencing technology was used in generating NGS data. Also, the NGS samples in this study were merged from different sources, which may cause batch effect. As shown in the SV PCA plot (Figure 3B and Figure S4D), the distance/difference between North and South Han Chinese is comparable or even larger than that between Han Chinese and Tibetan, which is unexpected, and inconsistent with both the population structure inferred from Lu et al. 2016 or with the demographic model constructed by SNV data in this study. indicating underlying batch effects in the SV genotyping results. The authors should perform comprehensive evaluation and remove those SVs that might be subject to batch effect.

(3)	We know that the Nanopore contained lots of sequencing errors, some of them may affect the resolution of SV breakpoints, which might greatly affect the genotyping accuracy of Paragraph. In addition, according to the authors (Line 193-194), more than 75% (56.7% + 19.5%) of the SVs are associated with repeats, how the authors guarantee the genotyping accuracy of these SVs? Are there any specific quality controls performed to address these issues in the NGS genotyping step? Are the genotyping results concordant with those in the LRS samples?

(4)	There are no experimental validations for the SVs, especially for the adaptive and archaic introgressed variants. Experiment validation could not only verify the variant but also check the accuracy of the genotyping results.

(5)	In population stratified SV section (line 265-284), the evidence of the functional roles of the two known SVs is indirect and weak. Unless functional experiment is provided, this part is more appropriate in the discussion. Likewise, in Line 316-318 'these results demonstrated that a considerable number of genes affected by population-stratified SVs...', as many SVs are intergenic variants including the 2,590-bp deletion near ZFAND2A highlighted in Line 285-302, whether SVs would affect the functions of these genes is still unknown, such conclusion would be misleading to the readers.

(6)	Although the authors are able to detect introgressed SVs, it is not sufficient to conclude the non-introgressed population-stratified SVs are de-novo mutations. Therefore the statement 'To distinguish these two hypotheses...' (Line 324-327) is incorrect. Even the SVs are not introgressed from archaic hominid, it is still possible that the variant is derived from modern human gene flow.

(7)	The sliding window of detecting natural selection is only 100 SNVs (approximately 100kb), but the window size for determining the natural selection and archaic introgression is 500kb. Such a large window may cause overestimate natural selection or archaic introgressed SVs.

(8)	The archaic introgressed SVs were characterized as the SVs with significant D-statistic/fd-statistic, however, such condition is not sufficient to define introgressed SVs. Only those co-segregating with archaic informative alleles are likely to be introgressed from archaic hominid. Especially for those SVs highlighted in Fig. 5, further analysis should be done to clarify whether the SV allele is on the archaic haplotype, such as the authors did in Fig. 6. Besides, incomplete lineage sorting (ILS) should be considered to formally distinguish an introgression event from a common ancestral event.

(9)	Again, no functional experiment or phenotype association was provided for the 335-bp deletion at 1p36.32, which was identified as a candidate archaic introgressed SV. It is unknown about how this SV would contribute to high altitude adaptation, or which adaptive trait/molecular function it would affect.

(10)	Besides, in the section on the population-stratified SVs and candidate adaptive genes, I wonder if the authors could discuss whether there is a difference in the effect of gene regulatory or gene expression level between the novel adaptive genes and previous reported adaptive genes? It will be great if the authors could evaluate the proportion of the novel adaptive SVs/genes account for the Tibetan HAA, which can help us better comprehend the importance of SVs in Tibetan HAA.

Minor issues:

Introduction

(11)	Reference falsely cited. In line 54-55, 'As an essential component of deleterious polymorphisms in humans in humans[2], SVs positively impact the formation of population diversity and adaptation[3]'. Reference [3] is about plaice not human. And I could not understand the logic of this sentence, why 'deleterious polymorphisms' would have a positive impact?

(12)	Line 64, EPAS1 does not belong to HIF-1 pathway.

(13)	Line 72, '... indicated shared genetic drift' is confusing, it should be 'signature of introgression'.

Results

(14)	Figure 1. Please add population label in Figure 1 A and B. Similarly, the Fig 1C and E would be more informative if the Tibetan and the Han Chinese were plotted separately. What does the height of the bar plot in Fig 1D represent?

(15)	Figure 2. P-values in B and D need to be adjusted considering of multiple testing.

(16)	Is the proportion of mutational mechanism as well as the functional enrichment pattern consistent with the previous studies?

(17)	Where does the number '14,928' come from in Line 190?

(18)	In the demographic model inference, the authors assumed symmetric migration between Tibetan and Han Chinese, what would be the results of non-symmetric migrations? The authors used YRI as African reference in the demographic inference, while used Biaka in other SV analyses, why didn't they consistently use Biaka in all the analyses?

(19)	How did the authors determine 0.1 as Fst cut-off to identify the population-stratified SVs? And why Figure 3D only highlight the SVs with Fst>0.15?

(20)	In Figure 4, have the P-values in the enrichment analyses been adjusted for multiple testing?

(21)	What does the 'ancient genomes' refer to in Line 341? Are they just chimpanzee genomes or also including Neanderthal and Denisova genomes?

(22)	How did the authors perform short read alignment for the chimpanzee and the archaic human data?

(23)	In line 360, which allele does the 'ancestral allele' refer to, the deletion or the non-deletion allele?

(24)	In line 362, do the proportions (100% and 88%) refer to the heterozygous percentage among the deletion carriers?

**Reviewer 2**

**Are you able to assess all statistics in the manuscript, including the appropriateness of statistical tests used?**

Yes, and I have assessed the statistics in my report.

**Comments to author:**

In this study, Quan et al. characterized the role of structural variation (SV) on high-altitude adaptation (HAA) in Tibetans. HAA has been well studied in Tibetans, and a role of adaptive introgression in this process has recently been demonstrated, but the impact of SVs on both HAA and adaptive introgression has yet to be studied. The paper should be of broad interest to others in the field. The authors characterized SVs using long-read nanopore technology on a relatively small set of samples (15 Tibetan and 10 Han) followed by NGS characterization of SVs on a larger sample for population genetic inferences. This study underscores the ubiquity of SVs in human populations, the difficulties of thorough SV detection using short-read NGS methods, along with their potential role in population differentiation and adaptation. The study is very interesting and the methods appear to be robust. I have a number of questions, which are primarily related to clarification of the methods and results. I suggest that the article be accepted for publication once these questions are addressed.

1. SV detection. Three different methods were used for SV detection. It would be interesting to show how many SVs were characterized by one, two, or three methods. Are SVs that are characterized by multiple methods more likely to be reliable based on the other metrics used, e.g. the number of supporting reads.

2. SV loss of function (LoF) variants. How were novel, previously undiscovered SVs characterized as loss of function variants?

3. Discovery of SVs in a Tibetan and Han population by long-read sequencing. 74% of reliable SVs have breakpoints within and interval of 100bp. Is this the threshold used to consider SVs shared across samples? If so, what criterion is this threshold based on?

4. Distribution of SVs across repeat sequences and functional genomic regions. The authors show that SINEs and LINEs generate numerous SVs via transposition and/or non-allelic homologous recombination (NAHR). It would be interesting to know what percentage of SINE and LINE associated SVs are associated with transposition versus NAHR, ie which is more prominent.

5. Population structure and Demographic inference: Why where the Chinese samples "artificially divided" into four subgroups based on geography? The NGS data could be used to more accurately assay population structure among these samples. Have the authors checked whether their geography based groups are in fact

genetically (genomically) distinct subgroups? (This does not appear to be the case for the Tibetan groups based on PCA analysis with SVs).

6. Population structure and Demographic inference: Related to the previous question, it would be very interesting to know whether population structure based on SNVs is similar to what is seen for SVs. If so, this would support the SV genotyping approach used here.

7. Population structure and Demographic inference: Give the issues with NGS based genotyping of SVs, it would be important to assess deviations from Hardy-Weinberg equilibrium prior to making conclusions about differences in homozygosity/heterozygosity.

8. Population-stratified SVs and candidate adaptive genes: Deletions are far more common among population-stratified and putatively adaptive variants than insertions. Is this enrichment greater than expected given the overall excess of deletion SVs? If so, what do the authors attribute this to?

9. Population-stratified SVs and candidate adaptive genes: Most of the high FST SVs shown in Table 1 have higher frequency in the Tibetan population compared to the Han population. Why is this? Could this be an artifact of the detection method?

10. Population-stratified SVs and candidate adaptive genes: Is the overall distribution of FST for SVs different than what is seen for SNVs, ie are SVs more population-specific than SNVs?

11. Signatures of positive selection and archaic introgression: It is not clear why the authors used physical distance of SVs (<550 kb) to a significant signature rather than linkage disequilibrium (LD) given the availability of SV genotypes. LD would be a more rigorous way to link SVs to genomic signatures of adaptation.

**Authors Response**

**Point-by-point responses to the reviewers’ comments:**

*Response to Comments from Reviewer #1*

Major issue 1:

(1) Ouzhuluobu et al. 2020 has already used PacBio long-read sequencing (LRS) technology to report a comprehensive SV map in Tibetan (ZF1), and other studies have used PacBio LRS in Han Chinese such as HX1 (Shi et al. 2016) and NH1 (Du et al. 2019), as well as those discovered in 15 worldwide samples (Audano et al. 2019), but the authors didn't compare their findings with any of the LRS studies. How many of the SVs in this study are novel if including those LRS SV studies in the comparison? Besides, Ouzhuluobu et al. 2020 provided a candidate list of adaptive SVs, it is unclear about which adaptive signals are novel.

*Response:*

*Thank you for your suggestion. In the revised version (Lines 137-144), we compared the SVs in our study with the SVs identified from the single assembly of Tibetan (ZF1 [2]) and Han (HX1 [3]), and another multi-population SV collection (Audano et al. 2019 [1]). We did not choose NH1 because this dataset is currently not available in public. When compared to the multi-population SV study, we found that 33.5% (n = 12,797) of our calls is novel, which is much more than those identified from ZF1 (n = 4,005) or HX1 (n = 3,908) (Fig. S1I). Meanwhile, 29.4% (n = 11,220) of our calls is novel compared to the merged set of all these long-read findings (Fig. S1I). If we take all these long-read and short-read findings as a reference, 10% (n = 3,669) of our SVs remains unresolved before (Fig. 1E). Among 3,669 novel SVs, 278 SVs are defined as shared (identified in all samples) or major variants (identified in ≥ 50% of samples) in our data. These results suggest that it is necessary to generate a comprehensive catalog of SVs through population-based methods which have the advantage in detecting novel SV events of singletons or even shared ones.*

*In the revised version, we identified a total of 69 Tibetan-Han stratified SVs (Table S9). Among these 69 SVs, five were in the candidate list of adaptive SVs from ZF1 (Ouzhuluobu et al. 2019). Therefore, 64 Tibetan-Han stratified SVs in this study are novel compared to their reports. Notably, the top four candidates in our study are enlisted in their study (Table S9), suggesting that the top stratified SVs are consistent across different studies. Please see the revised version (Line 416-421).*

*Besides, we found that 94 of 124 candidates reported from ZF1 were also detected in our LRS calls but exhibit no significant difference between Tibetans (n = 81) and Hans (n = 84) in our NGS collection. The candidates (30/124) which are significantly stratified between Tibetans (n = 38) and Hans (n = 39) in their study while not included in our LRS calls are almost duplications (n = 24). Supplemental Table 9 in their reports showed that 45.8% of duplications (11/24) are marked as imprecise, in contrast to 8.3% of deletions (6/72) and 0% of insertions. We obtained SVs supported by at least two methods, which could filter out some imprecise SV events. Among 69 adaptive SVs in our study, 30 ones haven’t been detected in their LRS calls, suggesting that the population-based method is also necessary to study population-stratified SVs.*

Major issue 2:

(2) The authors didn't mention which sequencing technology was used in generating NGS data. Also, the NGS samples in this study were merged from different sources, which may cause batch effect. As shown in the SV PCA plot (Figure 3B and Figure S4D), the distance/difference between North and South Han Chinese is comparable or even larger than that between Han Chinese and Tibetan, which is unexpected, and inconsistent with both the population structure inferred from Lu et al. 2016 or with the demographic model constructed by SNV data in this study. indicating underlying batch effects in the SV genotyping results. The authors should perform comprehensive evaluation and remove those SVs that might be subject to batch effect.

*Response:*

*Thank you for your suggestion. The short-read whole-genome sequencing (WGS) dataset generated by our lab was sequenced by the Illumina HiSeq X Ten. We have added the information of the sequencing platform for all NGS datasets to the Supplementary Methods (Short-read data aggregation).*

*We focused on the underlying batch effect for the genotyping results (Lines 208-213). We added another published NGS collection including 45 Northern Han Chinese and 45 Southern Han Chinese samples, helping us to assess the concordance of SV calls between all pairs of the three short-read WGS collections used for genotyping. These collections are marked as C1: 43 Tibetans and 46 Hans; C2: 38 Tibetans and 39 Hans (Lu et al., 2016); C3: 90 Hans (Lan et al., 2017).*

*As suggested by previous studies [4, 5], we firstly exclude SVs with a missing rate > 0.05 for any collections. Then we counted the number of homozygous (1/1), heterozygous (1/0), reference (0/0) and missing sites (./.) for each SV, and performed chi-squared tests between all pairs of the three collections for Tibetans (C1 vs C2) and Hans (C1 vs C2, C2 vs C3, C1 vs C3) respectively. Because only two collections contain Tibetan samples, we could assess the potential batch effect by four pair comparisons. P-value was corrected by the Benjamini-Hochberg (BH) method. If SVs failed in only one comparison (p-value < 0.05), we marked the variant with a ‘VARIABLE_ACROSS_BATCHES’ tag in the VCF filter and detailed results for the Tibetan-Han stratified SVs were recorded in Table S9. All SVs that failed in more than one comparison (p-value < 0.05) were defined as batch effect and were excluded from the following analysis.*

*After ﬁltering of batch effects and HWE, we obtained 10,387 successfully recalled SVs (16,953 in the original manuscript). Principle component analysis (PCA) revealed two major clusters in Chinese populations (Fig. 3B), including Tibetans and Hans, which is consistent with the results for whole-genome SNVs (Fig. S4I). This result is also confirmed by the best-fitting admixture model (Fig. 3C) with the minimum cross-validation error (K = 3). Notably, we still observed that HANS exhibited considerable genetic distance from HANN, which is consistent with the main Chinese population clusters reported by a recent study [6].*

Major issue 3:

(3) We know that the Nanopore contained lots of sequencing errors, some of them may affect the resolution of SV breakpoints, which might greatly affect the genotyping accuracy of Paragraph. In addition, according to the authors (Line 193-194), more than 75% (56.7% + 19.5%) of the SVs are associated with repeats, how the authors guarantee the genotyping accuracy of these SVs? Are there any specific quality controls performed to address these issues in the NGS genotyping step? Are the genotyping results concordant with those in the LRS samples?

*Response:*

*Given the impact from relatively high sequencing errors of Nanopore sequencing technology, we used a multi-platform calling method and re-genotyping pipeline as suggested by Sniffle. SV calling was performed per each sample using Sniffles, NanoSV, and SVIM, five minimum supporting reads are required per each method. Then we keep SVs supported by at least two methods. Using this method, we filtered out some imprecise SV events and improve the resolution of SV breakpoints, which could benefit genotyping in NGS data.*

*In the genotyping pipeline, we took Paragraph [7] to genotype each genome and set the maximum allowed read count for SVs to 20 times the mean genome coverage for each collection we used. Besides, we replaced all genotypes which failed to pass any filters by Paragraph with missing genotypes (./.). This method excludes variants having genotype conflicts or missing genotypes in one or more breakpoints.*

*Besides, as you suggest, we performed the validation of 16 adaptive SVs using PCR and Sanger sequencing, 87.5% of the candidates (14/16) successfully passed the validation. We described the method in the response to Major issue 4.*

*To assess the concordance between the genotyping results for NGS and the genotypes for the LRS samples, we performed short-read whole-genome sequencing to 15 Tibetans used for SV calling by LRS. Again, we took Paragraph to genotype each NGS genome and calculated Pearson’s correlation coefficient for genotypes per sample between NGS and LRS. We found that genotypes of all 15 samples were significantly correlated (Table S24).*

Major issue 4:

(4) There are no experimental validations for the SVs, especially for the adaptive and archaic introgressed variants. Experiment validation could not only verify the variant but also check the accuracy of the genotyping results.

*Response:*

*Thank you for your suggestion. In the revised version (Lines 259-264), we performed the validation of SVs using PCR and Sanger sequencing. Among 69 adaptive SVs, eight SVs have no interactions with any repeat regions. To see whether it is more difficult to validate SVs in repetitive regions, we chose eight non-repeat SVs and the top 8 adaptive SVs which have interactions with repeat elements. Finally, we found that 87.5% of the candidates (14/16) successfully passed the validation, including 7 non-repeat SVs and 7 top adaptive SVs (Table S10).*

Major issue 5:

(5) In population stratified SV section (line 265-284), the evidence of the functional roles of the two known SVs is indirect and weak. Unless functional experiment is provided, this part is more appropriate in the discussion. Likewise, in Line 316-318 'these results demonstrated that a considerable number of genes affected by population-stratified SVs...', as many SVs are intergenic variants including the 2,590-bp deletion near ZFAND2A highlighted in Line 285-302, whether SVs would affect the functions of these genes is still unknown, such conclusion would be misleading to the readers.

*Response:*

*Thank you for your suggestion. We have moved these descriptions to the Discussion section in the revised version (Lines 425-440, 451-467), and only the results for enrichment analysis of candidate adaptive genes were retained.*

Major issue 6:

(6) Although the authors are able to detect introgressed SVs, it is not sufficient to conclude the non-introgressed population-stratified SVs are de-novo mutations. Therefore the statement 'To distinguish these two hypotheses...' (Line 324-327) is incorrect. Even the SVs are not introgressed from archaic hominid, it is still possible that the variant is derived from modern human gene flow.

*Response:*

*We apologize for the inaccuracy of the statement. In the revised version (Lines 304-306), we have changed the corresponding description to 'To determine whether there are introgressed SVs contribute to the local adaptation of Tibetan highlanders, we searched for ….'*

Major issue 7:

(7) The sliding window of detecting natural selection is only 100 SNVs (approximately 100kb), but the window size for determining the natural selection and archaic introgression is 500kb. Such a large window may cause overestimate natural selection or archaic introgressed SVs.

*Response:*

*As suggested by a recent study [8], we detected selection signals for a sliding window of 100 SNVs and a step size of 50 SNVs, and then determined selection candidates if they were flanked by a significant window (p-value < 0.05, ±500 kb). Although this large window may lead to an overestimate of signatures of natural selection or archaic introgression around Tibetan-Han stratified SVs, this method can quickly screen out all adaptive introgressed candidates. The results in Fig. 5 summarize the evidence for adaptive introgression around the gene closed to these population-stratified SVs, providing a valuable resource for future studies on introgressed haplotypes or variants in local populations. However, these SVs are not necessarily adaptive introgressed SVs. So we further validate these SVs in ancient genomes (Neandertals and Denisovans), and three deletions were supported by at least one ancient genome with short-read sequencing data. When we focused on the three key candidates, we can see that the window exhibiting significant selection signals is no more than 100 kb from the breakpoint of SVs. Besides, it should be noted that SVs which were not successfully recalled in any ancient genomes were not necessarily absent from ancient humans, especially considering the short-read of ancient genomes. We believe that the results in Fig. 5 (±500 kb) are suggestive for future research, but the current method cannot determine whether these SVs that cannot be genotyped in short-read data of hominins are introgressed SVs.*

Major issue 8:

(8) The archaic introgressed SVs were characterized as the SVs with significant D-statistic/fd-statistic, however, such condition is not sufficient to define introgressed SVs. Only those co-segregating with archaic informative alleles are likely to be introgressed from archaic hominid. Especially for those SVs highlighted in Fig. 5, further analysis should be done to clarify whether the SV allele is on the archaic haplotype, such as the authors did in Fig. 6. Besides, incomplete lineage sorting (ILS) should be considered to formally distinguish an introgression event from a common ancestral event.

*Response:*

*Thank you for the suggestion. We apologize for the ambiguous description. Indeed, it is not sufficient to define introgressed SVs merely by significant D-statistic/fd-statistic. To validate these SVs in ancient genomes, we applied the same graph-based genotyping approach to archaic hominins and chimpanzees. The reason why haplotype clustering analysis was not performed for all candidate SVs in Fig. 5 is that only three deletions were supported by at least one ancient genome with short-read sequencing data. As we mentioned in the response to Major issue 7, the current method cannot determine whether these SVs that cannot be genotyped in hominins are introgressed SVs, considering the short-read of existing ancient genomes.*

*It is challenging to distinguish incomplete lineage sorting (ILS) and introgression because they are not mutually exclusive processes [9] and generate similar patterns of shared genetic diversity [10]. As suggested by a recent study [8], we calculated the Patterson's D-statistics as described in Martin et al. 2015, which can detect gene flow despite the existence of ILS [11]. Given three populations and an outgroup with the relationship (((YRI, TIB), ANC), CHIMP), a significant excess of ABBAs over BABAs is indicative either of gene flow between TIB and ANC, or some form of nonrandom mating or structure in the population ancestral to YRI, TIB, and ANC. Under the null hypothesis of no gene flow and random mating in the ancestral population, D-statics will approach zero, regardless of differences in effective population sizes. A D-statics significantly greater than zero is indicative of a significant excess of shared derived alleles between TIB and ANC. If a formal distinguish is needed for some specific introgressed candidates, as you suggest, we need to perform further analysis.*

Major issue 9:

(9) Again, no functional experiment or phenotype association was provided for the 335-bp deletion at 1p36.32, which was identified as a candidate archaic introgressed SV. It is unknown about how this SV would contribute to high altitude adaptation, or which adaptive trait/molecular function it would affect.

*Response:*

*Thank you for the suggestion. In the revised version (Lines 355-388), we performed a preliminary functional experiment for the 335bp deletion and analyzed phenotype associations in a larger population.*

*To explore the associations between the 335 bp deletion at 1p36.32 and phenotypes, we used blood samples from 418 Tibetan highlanders previously collected by our lab and measured 51 quantitative traits. Using PCR and sanger sequencing, we genotyped the 335 bp deletion in these Tibetans as well as 72 Hans. The genotyping frequency of the deletion is similar to the estimates from the NGS data (AF = 0.102 in Tibetans and AF = 0.236 in Hans). Consistent with the findings in the NGS data, deletion carriers exhibited high rates of heterozygosity in both Tibetans (94%) and Hans (87%). We then performed association analyses on these samples and found that the deletion tends to associate with some blood indices, including ALT (Alanine aminotransferase; p = 0.006, FDR = 0.319) and ALB (Albumin; p = 0.043, FDR = 0.478), which are biomarkers of liver function, but not significant after multi testing correction (Fig. S12). Consistent with this finding, the association tests for 91 quantitative traits collected from 2,849 Tibetan samples [12] with 55 SNVs in strong LD (r2 > 0.8) with this deletion, confirmed the connection with liver functions, as indicated by ALT (p = 0.003, FDR = 0.126), AST (Aspartate transaminase; p = 0.001, FDR = 0.047), and ALP (Alkaline phosphatase; p = 0.018, FDR = 0.417) (Additional file 1: Fig. S13).*

*To explore the function of the 335 bp deletion at 1p36.32, we further inspected LD-linked eQTLs for this deletion using QTLbase [13]. Among all the significant eQTLs (Additional file 2: Table S19), rs7546695 and rs6681331 (r2 = 0.82 with this deletion) are related to the expression of the two closest genes to this deletion respectively, ACTRT2 (actin related protein T2) and PRDM16 (PR/SET domain 16). Through luciferase reporter gene assays in HUVEC and K562 cell lines, we found that compared with the wild-type sequence, the 335 bp deletion could significantly reduce the luciferase activity of the reporter with the promoter region of PRDM16 but not with ACTRT2 (Fig. S14). Furthermore, we designed guide RNAs using the CRISPR design tool to knockout (KO) the sequence of this deletion in the HUVEC cell line. After genotyping by Sanger sequencing, we acquired candidate KO clones and found that these KO clones exhibit a significantly lower expression of PRDM16 compared to the control (Fig. S14H). These results suggest that the 335 bp deletion at 1p36.32 could down-regulate the expression of PRDM16. PRDM16 which is located at 66 kb downstream of this deletion, is a key regulator of the thermogenic program that controls the endogenous transformation of white to brown adipose tissues in response to cold [14, 15], and has been reported to exhibit up-regulated expression in the Tibetan pig [16]. Therefore, the relatively low frequency of this deletion could contribute to a quick response to the cold environment in High altitude regions.*

*In summary, the 335 bp deletion at 1p36.32, which is located in a region with weak positive selection and likely to be introgressed from the ancient humans, might benefits cold environment adaptation through regulation of PRDM16, accompanied by potential protective effect for liver functions.*

Major issue 10:

(10) Besides, in the section on the population-stratified SVs and candidate adaptive genes, I wonder if the authors could discuss whether there is a difference in the effect of gene regulatory or gene expression level between the novel adaptive genes and previous reported adaptive genes? It will be great if the authors could evaluate the proportion of the novel adaptive SVs/genes account for the Tibetan HAA, which can help us better comprehend the importance of SVs in Tibetan HAA.

*Response:*

*In the revised version (Lines 264-269, 275-278), we made a comparison between SVs and SNVs.*

*First of all, not all Tibetan-Han stratified SVs can be tagged with SNVs in linkage disequilibrium (LD, r2 > 0.9). Among 69 Tibetan-Han stratified SVs, eight candidates have no linked SNVs in LD with r2 > 0.8, while eighteen ones have no linked SNVs in LD with r2 > 0.9 (Table S9), suggesting that these SVs can represent some independent variations related to adaptation.*

*Secondly, the effect of SVs on the three-dimensional genome is more direct, which could be used to predict the creation or deletion of high-order genomic loops. These results not only identified multiple adaptive genes potentially regulated by population-stratified SVs far from their breakpoints, but also provide new insights into the regulatory mechanism of known adaptive genes (Fig. 4C).*

*Thirdly, we want to assess whether significant differences exist for the effect size on the eGene identified from SVs and SNVs. Using all significant single-tissue eQTLs for each eGene identified from our study, we calculated the rank order for the effect size of SV-associated SNVs linked by LD (r2 > 0.8) (Table S11). We found that 80.7% (96/119) of SV-associated eQTLs exhibited the top 10% effect size to the corresponding eGene (Fig. 4B). These results suggest that SVs could have an important regulatory effect on adaptive genes.*

Minor issue 1:

(11) Reference falsely cited. In line 54-55, 'As an essential component of deleterious polymorphisms in humans in humans[2], SVs positively impact the formation of population diversity and adaptation[3]'. Reference [3] is about plaice not human. And I could not understand the logic of this sentence, why 'deleterious polymorphisms' would have a positive impact?

*Response:*

*We apologize for the ambiguous citation. In the study of Abel et al. 2020, deleterious effects were predicted as equivalent to loss-of-function coding alleles. So we want to describe the negative effect from SVs could help local adaptation when facing a new environment. For brevity, this sentence has been removed from the revised version.*

Minor issue 2:

(12) Line 64, EPAS1 does not belong to HIF-1 pathway.

*Response:*

*We apologize for the incorrect description. In the revised version (Lines 63-65), we have changed the corresponding description to '… and successfully identified two essential genes related to the HIF (hypoxia-inducible transcription factor) pathway, EPAS1 (endothelial PAS domain protein 1) and EGLN1 (egl-9 family hypoxia inducible factor 1).'*

Minor issue 3:

(13) Line 72, '... indicated shared genetic drift' is confusing, it should be 'signature of introgression'.

*Response:*

*Thank you for the suggestion. In the revised version (Lines 72-73), we have changed the corresponding description to '… by derived alleles from five SNVs in the EPAS1 region indicated the signature of introgression from extinct non-AMH populations to Tibetans. '*

Minor issue 4:

(14) Figure 1. Please add population label in Figure 1 A and B. Similarly, the Fig 1C and E would be more informative if the Tibetan and the Han Chinese were plotted separately. What does the height of the bar plot in Fig 1D represent?

*Response:*

*Thank you for the suggestion. We have added population labels in Fig. 1A and 1B (Red are Tibetans and Blue are Hans). The separated plots of Fig 1C and 1E have been added in Fig. S1. We didn’t observe a significant difference in the proportion of discovery category between Tibetans and Hans. Compared to both short-read and long-read sequencing data, novel SVs for Tibetans are more than Hans, partly because we used more Tibetan samples than Hans (15 vs 10).*

*The bar plot in Fig 1D is a histogram for each chromosome, which shows the SV count for each 500 kb window (Lines 785). This plot could show us the increase in SV density within the last five Mb of chromosome arms (p-value < 0.001, permutation test), with the largest increase for major variants (fold change [FC] = 5.48) and the lowest for shared variants (FC = 4.18).*

Minor issue 5:

(15) Figure 2. P-values in B and D need to be adjusted considering of multiple testing.

*Response:*

*Thank you for the suggestion. In the revised version (Methods for Genomic background model), we expanded randomly shuffled background to 1,000 SV sets using BEDTools to perform overlap enrichment analysis of SVs versus genomic elements (Fig. 2). P-value was calculated by permutation test. After Bonferroni correction, only one active chromatin region (Flanking Bivalent TSS/Enh) which was significant depleted before multiple testing changed to no significant (Table S4).*

Minor issue 6:

(16) Is the proportion of mutational mechanism as well as the functional enrichment pattern consistent with the previous studies?

*Response:*

*The result of the functional enrichment pattern is consistent with many large-scale population studies on SV, such as Abel et al. 2020, Sudmant et al. 2015, Collins et al. 2020, etc. At first, SVs rarely locate near genes that are intolerant to LoF variations. Secondly, SVs are strongly depleted in active chromatin regions and enriched in inactive regions.*

*In our study, we used a simplified process to infer the formation mechanism of SVs without considering the nonhomologous recombination (NHR), because we didn’t estimate the sequence identity between the two breakpoints. We inferred the formation mechanism of SVs to explore the contribution of repeat elements. If not considering NHR, NAHR is reported as the major formation mechanism of deletions, while transposable elements introduce the most insertions (Mills et al., 2011, Sudmant et al., 2015, Carvahlho et al., 2016), which is consistent with our study.*

Minor issue 7:

(17) Where does the number '14,928' come from in Line 190?

*Response:*

*We apologize for the ambiguous description. It is the number of SVs that failed to be recalled by NGS (31,881 - 16,953 = 14,928). In the revised version (Lines 195-198), the number changed to 12,075 (26,279 - 14,204 = 12,075) due to additional variant and sample quality control.*

Minor issue 8:

(18) In the demographic model inference, the authors assumed symmetric migration between Tibetan and Han Chinese, what would be the results of non-symmetric migrations? The authors used YRI as African reference in the demographic inference, while used Biaka in other SV analyses, why didn't they consistently use Biaka in all the analyses?

*Response:*

*We have calculated the asymmetric model for TIB-HANN-HANS, and the likelihood (-149,756 vs -152,792) and AIC (299,540 vs 305,605) are slightly better than the symmetric models we described in the demographic inference. The divergence time between Tibetans and Hans (16,278 vs 17,640 years) is also similar to our reports in the manuscript. However, it took too much time to reach convergence due to the increased number of parameters. So we chose a simplified model.*

*The reason why we used YRI in the demographic inference and following analysis is that we couldn’t get the complete whole-genome SNV data of all 24 Biaka samples from SGDP. While YRI has a large number of samples in the 1000 Genomes Project which is comparable to the number of combined groups of Tibetans and Hans for the demographic inference. Besides, we found that the frequency of the 335 bp deletion at 1p36.32 in YRI was equivalent to that found in Tibetan, so we wanted to see whether there was any difference in the haplotypes.*

Minor issue 9:

(19) How did the authors determine 0.1 as Fst cut-off to identify the population-stratified SVs? And why Figure 3D only highlight the SVs with Fst>0.15?

*Response:*

*We apologize for the ambiguous description. This is an empirical estimate (FST cut-off = 0.1). In the revised version, we obtained 10,387 successfully recalled SVs after batch effects and HWE ﬁltering. If sorted by FST, the minimum estimate of the top 1% SVs (n = 100) was 0.099. So this threshold is appropriate for screening the most significant population-stratified SVs.*

*In Fig 3D, we only mark the points with FST > 0.15 just to keep the text from getting too crowded. Only the top 10 candidates were included in the revised version, and that’s not to say that only these SVs are significantly stratified.*

Minor issue 10:

(20) In Figure 4, have the P-values in the enrichment analyses been adjusted for multiple testing?

*Response:*

*Thank you for the suggestion. In the revised version, we made Bonferroni correction to the enrichment analyses in Fig. 4D and 4E. There are no significant biological functions in Fig. 4D, so we changed all bars to gray.*

Minor issue 11:

(21) What does the 'ancient genomes' refer to in Line 341? Are they just chimpanzee genomes or also including Neanderthal and Denisova genomes?

*Response:*

*We apologize for the ambiguous description. By 'ancient genomes', we mean Neanderthal and Denisovan genomes. We genotyped Tibetan-Han stratified SVs in the existing short-read sequencing data of hominins to see if they carry these variants. We did the same thing with the chimpanzee genomes to see if there were any SVs in the hominin genome that were not present in the chimpanzee genomes. While it is unnecessary to mention the chimpanzee genomes here so we change the description to 'After genotyping of 10 adaptive introgression candidates in archaic hominins, three deletions were supported by at least one ancient genome with short-read sequencing data' in the revised version (Lines 321-323).*

Minor issue 12:

(22) How did the authors perform short read alignment for the chimpanzee and the archaic human data?

*Response:*

*Chimpanzee WGS data were obtained from Great Ape Genome Project [17], including 17 individuals which were sequenced to 50 bp paired-end reads with an average coverage of 25x. Then we mapped short-read data to GRCh37 human reference using BWA-MEM (v0.7.19) with default parameters. As with the report from the project [17], no special treatment is necessary.*

*Three publicly available archaic hominin genomes were collected from the Department of Genetics in Max Planck Institute for Evolutionary Anthropology (https://www.eva.mpg.de/genetics). They have already provided alignments mapped to the human genome (hg19) for these sequences.*

Minor issue 13:

(23) In line 360, which allele does the 'ancestral allele' refer to, the deletion or the non-deletion allele?

*Response:*

*We mean the non-deletion allele which existed in chimpanzee genomes (Lines 338-340).*

Minor issue 14:

(24) In line 362, do the proportions (100% and 88%) refer to the heterozygous percentage among the deletion carriers?

*Response:*

*Yes. We apologize for the ambiguous description. In the revised version (Lines 341-343), we changed the description to 'this deletion exhibited high rates of heterozygosity for carriers in both Tibetans (100%) and Hans (88%)'*

Response to Comments from Reviewer #2

Major issue 1:

1. SV detection. Three different methods were used for SV detection. It would be interesting to show how many SVs were characterized by one, two, or three methods. Are SVs that are characterized by multiple methods more likely to be reliable based on the other metrics used, e.g. the number of supporting reads.

*Response:*

*Thank you for the suggestion. We have added the information for discoveries characterized by the different number of methods into Table S1. Five minimum supporting reads were also required in both the breakpoint discovery and re-genotyping process in our methods. In our opinion, SVs discovered through multiple methods should at least be more reliable than those simply screened by a limited number of supporting reads. Because all kinds of detection methods have shortcomings and vary greatly with different alignment algorithms. We performed SV calling using Sniffles, NanoSV, and SVIM, which have been reported to be compatible with NGMLR and show better accuracy and sensitivity than others [18].*

Major issue 2:

2. SV loss of function (LoF) variants. How were novel, previously undiscovered SVs characterized as loss of function variants?

*Response:*

*We annotated SVs for a range of potential effects on coding sequences using svtk [19]. As described in this method, we predicted an SV to cause genic LoF on an SV class-specific basis, as follows:*

*Deletions: any overlap with at least one exon.*

*Duplications: both breakpoints wholly contained within exons of the same gene, as we reasoned this would result in an altered amino acid sequence in all cases, and likely also result in a frameshift in most cases.*

*Insertions: insertion of any sequence directly into an exon.*

*Inversions: any inversion where one breakpoint is contained within a gene (exon or intron) and the other breakpoint is outside of the same gene, or any inversion where both breakpoints are contained within the same gene and the inversion overlaps at least one exon from that gene.*

*Translocations: any translocation breakpoint that overlaps an exon or intron.*

Major issue 3:

3. Discovery of SVs in a Tibetan and Han population by long-read sequencing. 74% of reliable SVs have breakpoints within and interval of 100bp. Is this the threshold used to consider SVs shared across samples? If so, what criterion is this threshold based on?

*Response:*

*We apologize for the ambiguous description. We used SURVIVOR to merge the SVs supported by at least two methods with a maximum allowed pairwise distance of 1,000 bp between breakpoints which is suggested by the software [20]. The reason why we calculate the proportion of SVs within breakpoints less than 100 bp is to explain that most SVs have consistent breakpoints resolutions (< 100 bp) compared to the maximum threshold of our calling method (< 1 kb). This is also an advantage of long-read sequencing for SV calling.*

Major issue 4:

4. Distribution of SVs across repeat sequences and functional genomic regions. The authors show that SINEs and LINEs generate numerous SVs via transposition and/or non-allelic homologous recombination (NAHR). It would be interesting to know what percentage of SINE and LINE associated SVs are associated with transposition versus NAHR, ie which is more prominent.

*Response:*

*For deletions in our data, 5,063 LINEs/SINEs are related to NAHR, while 9,444 of them contribute to transposable elements (TE). This result suggests that more LINEs /SINEs are associated with TE, but it is worth noting that this can be the result of our simplified definition of TE.*

Major issue 5:

5. Population structure and Demographic inference: Why where the Chinese samples "artificially divided" into four subgroups based on geography? The NGS data could be used to more accurately assay population structure among these samples. Have the authors checked whether their geography based groups are in fact genetically (genomically) distinct subgroups? (This does not appear to be the case for the Tibetan groups based on PCA analysis with SVs).

*Response:*

*We apologize for the inaccurate descriptions. We divided Chinese Han populations into two groups because Hans in North China (HANN) or South China (HANS) were divided into individual groups in the 1000 Genome Project. HANS with unique dialects and culture exhibited significant genetic distance from HANN, which is consistent with the main Chinese population clusters reported by a recent study [6]. After ﬁltering of batch effects and Hardy–Weinberg equilibrium (HWE), we can still observe the distance from PCA or admixture analysis (Fig. 3).*

*We divided Chinese Tibetans into Tibetans living above (TIBG) or below (TIBL) 4,000 meters mainly because it has been reported that adaptive variant frequency can be associated with altitudes, such as SNVs within EPAS1. Therefore, we want to explore whether population differences exist among regions of different altitudes.*

*Therefore, we changed the descriptions to 'we divided Chinese samples into four subgroups according to genetic distance reported in previous reports' in the revised version (Lines 213-216).*

Major issue 6:

6. Population structure and Demographic inference: Related to the previous question, it would be very interesting to know whether population structure based on SNVs is similar to what is seen for SVs. If so, this would support the SV genotyping approach used here.

*Response:*

*Thank you for the suggestion. In the revised version (Lines 220-222), we performed the PCA analysis for whole-genome SNVs which were used for demographic inference. Restricting the analysis to Chinese populations unveiled two major clusters (Fig. S4I), including Tibetans and Hans, which is consistent with the results for SVs after batch effects and Hardy–Weinberg equilibrium filtering (Fig. 3B).*

Major issue 7:

7. Population structure and Demographic inference: Give the issues with NGS based genotyping of SVs, it would be important to assess deviations from Hardy-Weinberg equilibrium prior to making conclusions about differences in homozygosity/heterozygosity.

*Response:*

*Thank you for the suggestion. In the revised version (Lines 208-210), we performed Hardy–Weinberg equilibrium filtering before population structural analysis. We observed that only 1,308 biallelic SVs localized to autosomes violates Hardy-Weinberg's assumptions for Tibetan, Hans, or Africans (Fig. S4).*

Major issue 8:

8. Population-stratified SVs and candidate adaptive genes: Deletions are far more common among population-stratified and putatively adaptive variants than insertions. Is this enrichment greater than expected given the overall excess of deletion SVs? If so, what do the authors attribute this to?

*Response:*

*In the revised version (Table S9), deletions are indeed enriched in population-stratified SVs (del = 63, ins = 6) compared to the whole SV calls (del = 6,138, ins = 4,205) after HWE and batch effect filtering using the chi-squared test. We speculate that this phenomenon should be related to the difficulty in genotyping insertions by NGS data. We found that the mean frequency of insertions in our NGS collections is 0.072 while the value of deletions is 0.227. However, the mean frequency of these population-stratified insertions (n = 6) is 0.321 while the value of deletions is 0.385. That is to say, only a small part of insertions with high frequency can be detected with Tibetan-Han differences because the overall frequency of insertions is low in our dataset. Besides, this may also be caused by the bias of the genotyping method. However, Paragraph didn’t report such errors.*

Major issue 9:

9. Population-stratified SVs and candidate adaptive genes: Most of the high FST SVs shown in Table 1 have higher frequency in the Tibetan population compared to the Han population. Why is this? Could this be an artifact of the detection method?

*Response:*

*In the revised version (Lines 250-253), we obtained 69 Tibetan-Han stratified SVs while 40 SVs have a higher frequency in Tibetans compared to Hans (Table S9). Among the top 10 stratified SVs, there is only one SV that has a higher frequency in Han (Table 1). In a recent study (Ouzhuluobu et al. 2019), there are 32 SVs that have a higher frequency in Tibetans out of 56 SVs. We think there are two reasons for this phenomenon. The most important point is that, compared to the Tibetan genome, the difference between the Chinese Han genome and the GRCh37 reference genome is much closer, leading to relatively fewer and less frequent variants including SVs and SNVs identified from the alignment to the human reference genome. Besides, FST estimates the population differentiation by comparing the subpopulation heterozygosity to the total expected heterozygosity. It is easier to detect the trend for a relatively low-frequency variant to a much higher one, while it is difficult to detect the trend for a relatively low-frequency variant to a much lower one.*

Major issue 10:

10. Population-stratified SVs and candidate adaptive genes: Is the overall distribution of FST for SVs different than what is seen for SNVs, ie are SVs more population-specific than SNVs?

*Response:*

*In Fig. S7B, we showed the results for FST of SNVs between Tibetans and Hans. We didn’t find significant differences between the overall distribution of FST for SVs and SNVs. Firstly, the mean of Weir and Cockerham FST estimate is 6.99×10-3 for SNVs and 6.23×10-3 for SVs. Secondly, the mean of the top 1% FST estimate is 0.11 for SNVs and 0.10 for SVs. The reason why Fig. 3C seems more population-stratified than Fig. S7B is that different lengths of windows were used for calculating FST. We used 100kb window-based FST for whole-genome SNVs and 20kb for SVs, which results in relatively low mean values in Fig. S7B. The overall distribution of FST for SVs is similar to SNVs.*

Major issue 11:

Signatures of positive selection and archaic introgression: It is not clear why the authors used physical distance of SVs (<550 kb) to a significant signature rather than linkage disequilibrium (LD) given the availability of SV genotypes. LD would be a more rigorous way to link SVs to genomic signatures of adaptation.

*Response:*

*As suggested by a recent study [8], we detected selection signals for a sliding window of 100 SNVs and a step size of 50 SNVs, and then determined selection candidates if they were flanked by a significant window (p-value < 0.05, ±500 kb). We have calculated the surrounding LD for three important adaptive introgressed SVs, but the signatures are not as obvious as the selection signal of SNVs in Fig. 6. The main problem is the difficulty in phasing SVs. We use shapeit4 to phase SNV, which is not suggested for SV. If phasing is not accurate, the method of searching for extend haplotype around SV will not get the expected result.*

*Reference*

*1. Audano PA, Sulovari A, Graves-Lindsay TA, Cantsilieris S, Sorensen M, Welch AE, Dougherty ML, Nelson BJ, Shah A, Dutcher SK, et al: Characterizing the Major Structural Variant Alleles of the Human Genome. Cell 2019, 176:663-675.e619.*

*2. Ouzhuluobu, He Y, Lou H, Cui C, Deng L, Gao Y, Zheng W, Guo Y, Wang X, Ning Z, et al: De novo assembly of a Tibetan genome and identification of novel structural variants associated with high altitude adaptation. bioRxiv 2019:753186.*

*3. Shi L, Guo Y, Dong C, Huddleston J, Yang H, Han X, Fu A, Li Q, Li N, Gong S, et al: Long-read sequencing and de novo assembly of a Chinese genome. Nature Communications 2016, 7:12065.*

*4. Collins RL, Brand H, Karczewski KJ, Zhao X, Alföldi J, Francioli LC, Khera AV, Lowther C, Gauthier LD, Wang H, et al: A structural variation reference for medical and population genetics. Nature 2020, 581:444-451.*

*5. Jakubosky D, D’Antonio M, Bonder M, Smail C, Donovan M, Greenwald WW, D’Antonio-Chronowska A, Matsui H, Consortium iQTL, Stegle O, et al: Genomic properties of structural variants and short tandem repeats that impact gene expression and complex traits in humans. bioRxiv 2019:714477.*

*6. Cao Y, Li L, Xu M, Feng Z, Sun X, Lu J, Xu Y, Du P, Wang T, Hu R, et al: The ChinaMAP analytics of deep whole genome sequences in 10,588 individuals. Cell research 2020:1-15.*

*7. Chen S, Krusche P, Dolzhenko E, Sherman RM, Petrovski R, Schlesinger F, Kirsche M, Bentley DR, Schatz MC, Sedlazeck FJ, Eberle MA: Paragraph: A graph-based structural variant genotyper for short-read sequence data. bioRxiv 2019:635011.*

*8. Hsieh P, Vollger MR, Dang V, Porubsky D, Baker C, Cantsilieris S, Hoekzema K, Lewis AP, Munson KM, Sorensen M, et al: Adaptive archaic introgression of copy number variants and the discovery of previously unknown human genes. Science 2019, 366:eaax2083.*

*9. Forsythe ES, Sloan DB, Beilstein MA: Divergence-Based Introgression Polarization. Genome Biology and Evolution 2020, 12:463-478.*

*10. Zhou Y, Duvaux L, Ren G, Zhang L, Savolainen O, Liu J: Importance of incomplete lineage sorting and introgression in the origin of shared genetic variation between two closely related pines with overlapping distributions. Heredity 2017, 118:211-220.*

*11. Zheng Y, Janke A: Gene flow analysis method, the D-statistic, is robust in a wide parameter space. BMC Bioinformatics 2018, 19:10.*

*12. Yang J, Jin Z-B, Chen J, Huang X-F, Li X-M, Liang Y-B, Mao J-Y, Chen X, Zheng Z, Bakshi A, et al: Genetic signatures of high-altitude adaptation in Tibetans. Proceedings of the National Academy of Sciences 2017, 114:4189-4194.*

*13. Zheng Z, Huang D, Wang J, Zhao K, Zhou Y, Guo Z, Zhai S, Xu H, Cui H, Yao H, et al: QTLbase: an integrative resource for quantitative trait loci across multiple human molecular phenotypes. Nucleic Acids Research 2019, 48:D983-D991.*

*14. Cohen P, Levy JD, Zhang Y, Frontini A, Kolodin DP, Svensson KJ, Lo JC, Zeng X, Ye L, Khandekar MJ, et al: Ablation of PRDM16 and Beige Adipose Causes Metabolic Dysfunction and a Subcutaneous to Visceral Fat Switch. Cell 2014, 156:304-316.*

*15. Seale P, Conroe HM, Estall J, Kajimura S, Frontini A, Ishibashi J, Cohen P, Cinti S, Spiegelman BM: Prdm16 determines the thermogenic program of subcutaneous white adipose tissue in mice. Journal of Clinical Investigation 2011, 121:96-105.*

*16. Lin J, Cao C, Tao C, Ye R, Dong M, Zheng Q, Wang C, Jiang X, Qin G, Yan C, et al: Cold adaptation in pigs depends on UCP3 in beige adipocytes. Journal of Molecular Cell Biology 2017, 9:364-375.*

*17. Prado-Martinez J, Sudmant PH, Kidd JM, Li H, Kelley JL, Lorente-Galdos B, Veeramah KR, Woerner AE, O’Connor TD, Santpere G, et al: Great ape genetic diversity and population history. Nature 2013, 499:471-475.*

*18. Coster W, Rijk P, Roeck A, Pooter T, D'Hert S, Strazisar M, Sleegers K, Broeckhoven C: Structural variants identified by Oxford Nanopore PromethION sequencing of the human genome. Genome Research 2019, 29:1178-1187.*

*19. Werling DM, Brand H, An J-Y, Stone MR, Zhu L, Glessner JT, Collins RL, Dong S, Layer RM, Markenscoff-Papadimitriou E, et al: An analytical framework for whole-genome sequence association studies and its implications for autism spectrum disorder. Nature Genetics 2018, 50:727-736.*

*20. Jeffares DC, Jolly C, Hoti M, Speed D, Shaw L, Rallis C, Balloux F, Dessimoz C, Bähler J, Sedlazeck FJ: Transient structural variations have strong effects on quantitative traits and reproductive isolation in fission yeast. Nature Communications 2017, 8:14061.*

**Second round of review**

**Reviewer 1**

The authors have addressed most of the issues, and the quality of the manuscript has been greatly improved. However, one issue remains, i.e., the identification of the introgressed SVs. D-statistic is not sufficient to determine whether a segment is from archaic introgression or not as it is an allele-frequency-based method. Ancestry inference at the haplotype level is required to rule out the possibility of false positives. The authors may apply haplotype-based archaic inference methods such as Sprime (Browning et al. 2018 Cell) to further distinguish whether the SV is on the archaic ancestry haplotype. ‘Two-thirds’ of the adaptive SVs were labeled as introgressed variants, which might mislead the readers if some of them were not truly introgressed from an archaic hominid. Therefore, I would suggest not to mark the SVs as archaic introgression candidates (Figure 5 and Table S9), unless they could be confidently identified from the signatures of the haplotype as the example of 335-bp deletion at 1p36.32 (Figure 6).

Also, there are two minor problems:

Line 251, ‘Fig. 3D; Error! Reference source not found.

Line 260, ‘...no interaction with repeat’ should be ‘...no intersection with’.

**Reviewer 2**

I am satisfied with the authors' responses to my comments and the revisions that they have made to the manuscript.

**Authors Response**

**Point-by-point responses to the reviewers’ comments:**

Response to Comments from Reviewer #1

Comment:

The authors have addressed most of the issues, and the quality of the manuscript has been greatly improved. However, one issue remains, i.e., the identification of the introgressed SVs. D-statistic is not sufficient to determine whether a segment is from archaic introgression or not as it is an allele-frequency-based method. Ancestry inference at the haplotype level is required to rule out the possibility of false positives. The authors may apply haplotype-based archaic inference methods such as Sprime (Browning et al. 2018 Cell) to further distinguish whether the SV is on the archaic ancestry haplotype. 'Two-thirds' of the adaptive SVs were labeled as introgressed variants, which might mislead the readers if some of them were not truly introgressed from an archaic hominid. Therefore, I would suggest not to mark the SVs as archaic introgression candidates (Figure 5 and Table S9), unless they could be confidently identified from the signatures of the haplotype as the example of 335-bp deletion at 1p36.32 (Figure 6).

*Response:*

*Thank you for your suggestion. We performed the S*-like statistic (Browning SR, et al. 2018) as an orthogonal method to rule out the possibility of false positives for introgression signals. Similar to the original S* statistic (Plagnol V, et al. 2016), this method utilizes linkage information and calculates a pairwise S*-like score T between the target population and the outgroup. We calculated the maximum score T for each sliding window defined by fd-statistic with a mutation rate of 1.5e-8 per site per generation. We found significant differences of scores between the simulation (mean = 2,369) and the real data (mean = 11,176). As suggested by a recent study (Hsieh P, et al. 2019), the p-value of each window was defined as the proportion of simulations (YRI-TIB-HAN) with scores greater than those in the real data. We determined the candidate introgressed SVs if they were flanked by a significant window (p-value < 0.05 for D-statistic, fd-statistic, and S*-like statistic). We also required that the score T of an introgressed segment in the real data is larger than 15,000 as a global control of false positives across all population-stratified SVs (the 95% quantile of score T in simulation data is 7,289). Using these criteria, we found that 25 introgression candidates remain significant in the revised version (there are 43 candidates in the original version), including 23 and 14 loci using Neanderthal and Denisovan genomes as the archaic reference respectively (Fig. 5; Table S8). Meanwhile, six adaptive introgression candidates remain significant in the revised version (there are 10 candidates in the original version). Notably, the three deletions which were supported by at least one ancient genome with short-read sequencing data remain significant in the revised version.*

Minor problems:

Also, there are two minor problems:

Line 251, ‘Fig. 3D; Error! Reference source not found.

Line 260, ‘...no interaction with repeat’ should be ‘...no intersection with’.

*Response:*

*We apologize for these mistakes. The problems have been corrected in the revised version.*
